# Implicating genes, pleiotropy, and sexual dimorphism at blood lipid loci through multi-ancestry meta-analysis

**DOI:** 10.1186/s13059-022-02837-1

**Published:** 2022-12-27

**Authors:** Stavroula Kanoni, Sarah E. Graham, Yuxuan Wang, Ida Surakka, Shweta Ramdas, Xiang Zhu, Shoa L. Clarke, Konain Fatima Bhatti, Sailaja Vedantam, Thomas W. Winkler, Adam E. Locke, Eirini Marouli, Greg J. M. Zajac, Kuan-Han H. Wu, Ioanna Ntalla, Qin Hui, Derek Klarin, Austin T. Hilliard, Zeyuan Wang, Chao Xue, Gudmar Thorleifsson, Anna Helgadottir, Daniel F. Gudbjartsson, Hilma Holm, Isleifur Olafsson, Mi Yeong Hwang, Sohee Han, Masato Akiyama, Saori Sakaue, Chikashi Terao, Masahiro Kanai, Wei Zhou, Ben M. Brumpton, Humaira Rasheed, Aki S. Havulinna, Yogasudha Veturi, Jennifer Allen Pacheco, Elisabeth A. Rosenthal, Todd Lingren, QiPing Feng, Iftikhar J. Kullo, Akira Narita, Jun Takayama, Hilary C. Martin, Karen A. Hunt, Bhavi Trivedi, Jeffrey Haessler, Franco Giulianini, Yuki Bradford, Jason E. Miller, Archie Campbell, Kuang Lin, Iona Y. Millwood, Asif Rasheed, George Hindy, Jessica D. Faul, Wei Zhao, David R. Weir, Constance Turman, Hongyan Huang, Mariaelisa Graff, Ananyo Choudhury, Dhriti Sengupta, Anubha Mahajan, Michael R. Brown, Weihua Zhang, Ketian Yu, Ellen M. Schmidt, Anita Pandit, Stefan Gustafsson, Xianyong Yin, Jian’an Luan, Jing-Hua Zhao, Fumihiko Matsuda, Hye-Mi Jang, Kyungheon Yoon, Carolina Medina-Gomez, Achilleas Pitsillides, Jouke Jan Hottenga, Andrew R. Wood, Yingji Ji, Zishan Gao, Simon Haworth, Noha A. Yousri, Ruth E. Mitchell, Jin Fang Chai, Mette Aadahl, Anne A. Bjerregaard, Jie Yao, Ani Manichaikul, Chii-Min Hwu, Yi-Jen Hung, Helen R. Warren, Julia Ramirez, Jette Bork-Jensen, Line L. Kårhus, Anuj Goel, Maria Sabater-Lleal, Raymond Noordam, Pala Mauro, Floris Matteo, Aaron F. McDaid, Pedro Marques-Vidal, Matthias Wielscher, Stella Trompet, Naveed Sattar, Line T. Møllehave, Matthias Munz, Lingyao Zeng, Jianfeng Huang, Bin Yang, Alaitz Poveda, Azra Kurbasic, Claudia Lamina, Lukas Forer, Markus Scholz, Tessel E. Galesloot, Jonathan P. Bradfield, Sanni E. Ruotsalainen, EWarwick Daw, Joseph M. Zmuda, Jonathan S. Mitchell, Christian Fuchsberger, Henry Christensen, Jennifer A. Brody, Miguel Vazquez-Moreno, Mary F. Feitosa, Mary K. Wojczynski, Zhe Wang, Michael H. Preuss, Massimo Mangino, Paraskevi Christofidou, Niek Verweij, Jan W. Benjamins, Jorgen Engmann, Noah L. Tsao, Anurag Verma, Roderick C. Slieker, Ken Sin Lo, Nuno R. Zilhao, Phuong Le, Marcus E. Kleber, Graciela E. Delgado, Shaofeng Huo, Daisuke D. Ikeda, Hiroyuki Iha, Jian Yang, Jun Liu, Ayşe Demirkan, Hampton L. Leonard, Jonathan Marten, Mirjam Frank, Börge Schmidt, Laura J. Smyth, Marisa Cañadas-Garre, Chaolong Wang, Masahiro Nakatochi, Andrew Wong, Nina Hutri-Kähönen, Xueling Sim, Rui Xia, Alicia Huerta-Chagoya, Juan Carlos Fernandez-Lopez, Valeriya Lyssenko, Suraj S. Nongmaithem, Swati Bayyana, Heather M. Stringham, Marguerite R. Irvin, Christopher Oldmeadow, Han-Na Kim, Seungho Ryu, Paul R. H. J. Timmers, Liubov Arbeeva, Rajkumar Dorajoo, Leslie A. Lange, Gauri Prasad, Laura Lorés-Motta, Marc Pauper, Jirong Long, Xiaohui Li, Elizabeth Theusch, Fumihiko Takeuchi, Cassandra N. Spracklen, Anu Loukola, Sailalitha Bollepalli, Sophie C. Warner, Ya Xing Wang, Wen B. Wei, Teresa Nutile, Daniela Ruggiero, Yun Ju Sung, Shufeng Chen, Fangchao Liu, Jingyun Yang, Katherine A. Kentistou, Bernhard Banas, Giuseppe Giovanni Nardone, Karina Meidtner, Lawrence F. Bielak, Jennifer A. Smith, Prashantha Hebbar, Aliki-Eleni Farmaki, Edith Hofer, Maoxuan Lin, Maria Pina Concas, Simona Vaccargiu, Peter J. van der Most, Niina Pitkänen, Brian E. Cade, Sander W. van der Laan, Kumaraswamy Naidu Chitrala, Stefan Weiss, Amy R. Bentley, Ayo P. Doumatey, Adebowale A. Adeyemo, Jong Young Lee, Eva R. B. Petersen, Aneta A. Nielsen, Hyeok Sun Choi, Maria Nethander, Sandra Freitag-Wolf, Lorraine Southam, Nigel W. Rayner, Carol A. Wang, Shih-Yi Lin, Jun-Sing Wang, Christian Couture, Leo-Pekka Lyytikäinen, Kjell Nikus, Gabriel Cuellar-Partida, Henrik Vestergaard, Bertha Hidalgo, Olga Giannakopoulou, Qiuyin Cai, Morgan O. Obura, Jessica van Setten, Xiaoyin Li, Jingjing Liang, Hua Tang, Natalie Terzikhan, Jae Hun Shin, Rebecca D. Jackson, Alexander P. Reiner, Lisa Warsinger Martin, Zhengming Chen, Liming Li, Takahisa Kawaguchi, Joachim Thiery, Joshua C. Bis, Lenore J. Launer, Huaixing Li, Mike A. Nalls, Olli T. Raitakari, Sahoko Ichihara, Sarah H. Wild, Christopher P. Nelson, Harry Campbell, Susanne Jäger, Toru Nabika, Fahd Al-Mulla, Harri Niinikoski, Peter S. Braund, Ivana Kolcic, Peter Kovacs, Tota Giardoglou, Tomohiro Katsuya, Dominique de Kleijn, Gert J. de Borst, Eung Kweon Kim, Hieab H. H. Adams, M. Arfan Ikram, Xiaofeng Zhu, Folkert W. Asselbergs, Adriaan O. Kraaijeveld, Joline W. J. Beulens, Xiao-Ou Shu, Loukianos S. Rallidis, Oluf Pedersen, Torben Hansen, Paul Mitchell, Alex W. Hewitt, Mika Kähönen, Louis Pérusse, Claude Bouchard, Anke Tönjes, Yii-Der Ida Chen, Craig E. Pennell, Trevor A. Mori, Wolfgang Lieb, Andre Franke, Claes Ohlsson, Dan Mellström, Yoon Shin Cho, Hyejin Lee, Jian-Min Yuan, Woon-Puay Koh, Sang Youl Rhee, Jeong-Taek Woo, Iris M. Heid, Klaus J. Stark, Martina E. Zimmermann, Henry Völzke, Georg Homuth, Michele K. Evans, Alan B. Zonderman, Ozren Polasek, Gerard Pasterkamp, Imo E. Hoefer, Susan Redline, Katja Pahkala, Albertine J. Oldehinkel, Harold Snieder, Ginevra Biino, Reinhold Schmidt, Helena Schmidt, Stefania Bandinelli, George Dedoussis, Thangavel Alphonse Thanaraj, Sharon L. R. Kardia, Patricia A. Peyser, Norihiro Kato, Matthias B. Schulze, Giorgia Girotto, Carsten A. Böger, Bettina Jung, Peter K. Joshi, David A. Bennett, Philip L. De Jager, Xiangfeng Lu, Vasiliki Mamakou, Morris Brown, Mark J. Caulfield, Patricia B. Munroe, Xiuqing Guo, Marina Ciullo, Jost B. Jonas, Nilesh J. Samani, Jaakko Kaprio, Päivi Pajukanta, Teresa Tusié-Luna, Carlos A. Aguilar-Salinas, Linda S. Adair, Sonny Augustin Bechayda, H. Janaka de Silva, Ananda R. Wickremasinghe, Ronald M. Krauss, Jer-Yuarn Wu, Wei Zheng, Anneke Iden Hollander, Dwaipayan Bharadwaj, Adolfo Correa, James G. Wilson, Lars Lind, Chew-Kiat Heng, Amanda E. Nelson, Yvonne M. Golightly, James F. Wilson, Brenda Penninx, Hyung-Lae Kim, John Attia, Rodney J. Scott, D. C. Rao, Donna K. Arnett, Steven C. Hunt, Mark Walker, Heikki A. Koistinen, Giriraj R. Chandak, Josep M. Mercader, Maria C. Costanzo, Dongkeun Jang, Noël P. Burtt, Clicerio Gonzalez Villalpando, Lorena Orozco, Myriam Fornage, EShyong Tai, Rob M. van Dam, Terho Lehtimäki, Nish Chaturvedi, Mitsuhiro Yokota, Jianjun Liu, Dermot F. Reilly, Amy Jayne McKnight, Frank Kee, Karl-Heinz Jöckel, Mark I. McCarthy, Colin N. A. Palmer, Veronique Vitart, Caroline Hayward, Eleanor Simonsick, Cornelia M. van Duijn, Zi-Bing Jin, Jia Qu, Haretsugu Hishigaki, Xu Lin, Winfried März, Vilmundur Gudnason, Jean-Claude Tardif, Guillaume Lettre, Leen M.‘t Hart, Petra J. M. Elders, Scott M. Damrauer, Meena Kumari, Mika Kivimaki, Pim van der Harst, Tim D. Spector, Ruth J. F. Loos, Michael A. Province, Esteban J. Parra, Miguel Cruz, Bruce M. Psaty, Ivan Brandslund, Peter P. Pramstaller, Charles N. Rotimi, Kaare Christensen, Samuli Ripatti, Elisabeth Widén, Hakon Hakonarson, Struan F. A. Grant, Lambertus A. L. M. Kiemeney, Jacqueline de Graaf, Markus Loeffler, Florian Kronenberg, Dongfeng Gu, Jeanette Erdmann, Heribert Schunkert, Paul W. Franks, Allan Linneberg, J. Wouter Jukema, Amit V. Khera, Minna Männikkö, Marjo-Riitta Jarvelin, Zoltan Kutalik, Cucca Francesco, Dennis O. Mook-Kanamori, Ko Willems van Dijk, Hugh Watkins, David P. Strachan, Niels Grarup, Peter Sever, Neil Poulter, Lee-Ming Chuang, Jerome I. Rotter, Thomas M. Dantoft, Fredrik Karpe, Matt J. Neville, Nicholas J. Timpson, Ching-Yu Cheng, Tien-Yin Wong, Chiea Chuen Khor, Hengtong Li, Charumathi Sabanayagam, Annette Peters, Christian Gieger, Andrew T. Hattersley, Nancy L. Pedersen, Patrik K. E. Magnusson, Dorret I. Boomsma, Allegonda H. M. Willemsen, LAdrienne Cupples, Joyce B. J. van Meurs, Mohsen Ghanbari, Penny Gordon-Larsen, Wei Huang, Young Jin Kim, Yasuharu Tabara, Nicholas J. Wareham, Claudia Langenberg, Eleftheria Zeggini, Johanna Kuusisto, Markku Laakso, Erik Ingelsson, Goncalo Abecasis, John C. Chambers, Jaspal S. Kooner, Paul S. de Vries, Alanna C. Morrison, Scott Hazelhurst, Michèle Ramsay, Kari E. North, Martha Daviglus, Peter Kraft, Nicholas G. Martin, John B. Whitfield, Shahid Abbas, Danish Saleheen, Robin G. Walters, Michael V. Holmes, Corri Black, Blair H. Smith, Aris Baras, Anne E. Justice, Julie E. Buring, Paul M. Ridker, Daniel I. Chasman, Charles Kooperberg, Gen Tamiya, Masayuki Yamamoto, David A. van Heel, Richard C. Trembath, Wei-Qi Wei, Gail P. Jarvik, Bahram Namjou, M. Geoffrey Hayes, Marylyn D. Ritchie, Pekka Jousilahti, Veikko Salomaa, Kristian Hveem, Bjørn Olav Åsvold, Michiaki Kubo, Yoichiro Kamatani, Yukinori Okada, Yoshinori Murakami, Bong-Jo Kim, Unnur Thorsteinsdottir, Kari Stefansson, Jifeng Zhang, YEugene Chen, Yuk-Lam Ho, Julie A. Lynch, Daniel J. Rader, Philip S. Tsao, Kyong-Mi Chang, Kelly Cho, Christopher J. O’Donnell, John M. Gaziano, Peter W. F. Wilson, Timothy M. Frayling, Joel N. Hirschhorn, Sekar Kathiresan, Karen L. Mohlke, Yan V. Sun, Andrew P. Morris, Michael Boehnke, Christopher D. Brown, Pradeep Natarajan, Panos Deloukas, Cristen J. Willer, Themistocles L. Assimes, Gina M. Peloso

**Affiliations:** 1 William Harvey Research Institute, Barts and the London School of Medicine and Dentistry, Queen Mary University of London, Charterhouse Square, London, EC1M 6BQ UK; 2 Department of Internal Medicine, Division of Cardiology, University of Michigan, Ann Arbor, MI 48109 USA; 3 Department of Biostatistics, Boston University School of Public Health, 801 Massachusetts Ave, Boston, MA 02118 USA; 4 Department of Genetics, Perelman School of Medicine, University of Pennsylvania, Philadelphia, PA 19104 USA; 5 Department of Statistics, The Pennsylvania State University, University Park, PA USA; 6 Huck Institutes of the Life Sciences, The Pennsylvania State University, University Park, PA USA; 7 VA Palo Alto Health Care Systems, Palo Alto, CA USA; 8 Department of Statistics, Stanford University, Stanford, CA USA; 9 Department of Medicine, Division of Cardiovascular Medicine, Stanford University School of Medicine, Stanford, CA 94305 USA; 10 Boston Children’s Hospital, EndocrinologyBoston, MA 02115 USA; 11 Program in Medical and Population Genetics, Broad Institute of Harvard and MIT, Cambridge, MA USA; 12 Department of Genetic Epidemiology, University of Regensburg, Regensburg, Germany; 13 McDonnell Genome Institute and Department of Medicine, Washington University, St. Louis, MO 63108 USA; 14 Department of Biostatistics, Center for Statistical Genetics, University of Michigan, Ann Arbor, MI USA; 15 Department of Computational Medicine and Bioinformatics, University of Michigan, Ann Arbor, MI 48109 USA; 16 Clinical Pharmacology, William Harvey Research Institute, Barts and The London School of Medicine and Dentistry, Queen Mary University of London, London, EC1M 6BQ UK; 17 Department of Epidemiology, Emory University Rollins School of Public Health, Atlanta, GA USA; 18 Atlanta VA Health Care System, Decatur, GA USA; 19 Department of Surgery, Stanford University School of Medicine, Stanford, CA USA; 20 deCODE Genetics/Amgen, Inc. Sturlugata 8, Reykjavik, 102 Iceland; 21 School of Engineering and Natural Sciences, University of Iceland, Sæmundargötu 2, Reykjavik, 102 Iceland; 22 Department of Clinical Biochemistry, Landspitali - National University Hospital of Iceland, Hringbraut, Reykjavik, 101 Iceland; 23 Division of Genome Science, Department of Precision Medicine, National Institute of Health, Chungcheongbuk-Do, South Korea; 24 Laboratory for Statistical Analysis, RIKEN Center for Integrative Medical Sciences, Yokohama, Japan; 25 Department of Ophthalmology, Graduate School of Medical Sciences, Kyushu University, Fukuoka, Japan; 26 Department of Statistical Genetics, Osaka University Graduate School of Medicine, Osaka, Japan; 27 Department of Allergy and Rheumatology, Graduate School of Medicine, The University of Tokyo, Tokyo, Japan; 28 Laboratory for Statistical and Translational Genetics, RIKEN Center for Integrative Medical Sciences, Yokohama, Japan; 29 Department of Biomedical Informatics, Harvard Medical School, Boston, MA USA; 30 Analytic and Translational Genetics Unit, Massachusetts General Hospital, Boston, MA USA; 31 K.G. Jebsen Center for Genetic Epidemiology, Department of Public Health and Nursing, NTNU, Norwegian University of Science and Technology, Trondheim, Norway; 32 MRC Integrative Epidemiology Unit (IEU), Bristol Medical School, University of Bristol, Oakfield House, Oakfield Grove, Bristol, BS8 2BN UK; 33 Clinic of Medicine, St. Olavs Hospital, Trondheim University Hospital, Trondheim, Norway; 34 Division of Medicine and Laboratory Sciences, University of Oslo, Oslo, Norway; 35 Institute for Molecular Medicine Finland (FIMM), HiLIFE, University of Helsinki, Tukholmankatu 8, 00014 Helsinki, Finland; 36 Department of Public Health and Welfare, Finnish Institute for Health and Welfare, Helsinki, Finland; 37 Department of Genetics, Institute for Biomedical Informatics, University of Pennsylvania, Perelman School of Medicine, Philadelphia, PA 19104 USA; 38 Center for Genetic Medicine, Northwestern University, Feinberg School of Medicine, Chicago, IL 60618 USA; 39 Department of Medicine (Medical Genetics), University of Washington, Seattle, WA USA; 40 Division of Biomedical Informatics, Cincinnati Children’s Hospital Medical Center, Cincinnati, OH USA; 41 Division of Clinical Pharmacology, Department of Medicine, Vanderbilt University Medical Center, Nashville, TN USA; 42 Department of Cardiovascular Medicine and the Gonda Vascular Center, Mayo Clinic, Rochester, MN USA; 43 Tohoku Medical Megabank Organization, Tohoku University, Sendai, 980-8573 Japan; 44 Wellcome Trust Sanger Institute, Hinxton, CB10 1SA UK; 45 Blizard Institute, Barts and the London School of Medicine and Dentistry, Queen Mary University of London, London, UK; 46 Fred Hutchinson Cancer Center, Division of Public Health Sciences, Seattle, WA 9810 USA; 47 Division of Preventive Medicine, Brigham and Women’s Hospital, Boston, MA 02215 USA; 48 Centre for Genomic and Experimental Medicine, Institute of Genetics and Cancer, University of Edinburgh, Western General Hospital, Edinburgh, EH4 2XU UK; 49 Usher Institute, The University of Edinburgh, Nine, Edinburgh Bioquarter, 9 Little France Road, Edinburgh, EH16 4UX UK; 50 Clinical Trial Service Unit and Epidemiological Studies Unit, Nuffield Department of Population Health, University of Oxford, Oxford, OX3 7LF UK; 51 Medical Research Council Population Health Research Unit, Nuffield Department of Population Health, University of Oxford, Oxford, OX3 7LF UK; 52 Center for Non-Communicable Diseases, Karachi, Sindh Pakistan; 53 Department of Population Medicine, Qatar University College of Medicine, QU Health, Doha, Qatar; 54 Survey Research Center, Institute for Social Research, University of Michigan, Ann Arbor, MI 48104 USA; 55 Department of Epidemiology, School of Public Health, University of Michigan, Ann Arbor, MI 48109 USA; 56 Program in Genetic Epidemiology and Statistical Genetics, Department of Epidemiology, Harvard T.H. Chan School of Public Health, 677 Huntington Avenue, Boston, MA 02115 USA; 57 Department of Epidemiology, Gillings School of Global Public Health, University of North Carolina, Chapel Hill, NC USA; 58 Sydney Brenner Institute for Molecular Bioscience, Faculty of Health Sciences, University of the Witwatersrand, Johannesburg, South Africa; 59 Wellcome Centre for Human Genetics, University of Oxford, Oxford, OX3 7BN UK; 60 Human Genetics Center, Department of Epidemiology, School of Public Health, Human Genetics, and Environmental Sciences, The University of Texas Health Science Center at Houston, Houston, TX 77030 USA; 61 Department of Epidemiology and Biostatistics, Imperial College London, London, W2 1PG UK; 62 Department of Cardiology, Ealing Hospital, London North West University Healthcare NHS Trust, Middlesex, UB1 3HW UK; 63 Department of Medical Sciences, Molecular Epidemiology and Science for Life Laboratory, Uppsala University, Uppsala, Sweden; 64 MRC Epidemiology Unit, University of Cambridge School of Clinical Medicine, Cambridge, CB2 0QQ UK; 65 Cardiovascular Epidemiology Unit, Department of Public Health and Primary Care, University of Cambridge, Strangeways Research Laboratory, Wort’s Causeway, Cambridge, CB1 8RN UK; 66 Center for Genomic Medicine, Kyoto University Graduate School of Medicine, Kyoto, Japan; 67 Department of Internal Medicine, Erasmus MC, University Medical Center Rotterdam, Rotterdam, the Netherlands; 68 Department of Biological Psychology, Vrije Universiteit Amsterdam, Amsterdam, The Netherlands; 69 Amsterdam Public Health Research Institute, Amsterdam UMC, the Netherlands; 70 Genetics of Complex Traits, University of Exeter Medical School, University of Exeter, Exeter, EX2 5DW UK; 71 Nanjing University of Chinese Medicine, Nanjing, 210029 Jiangsu China; 72 Research Unit of Molecular Epidemiology, Helmholtz Zentrum München, German Research Center for Environmental Health, Neuherberg, Germany; 73 Institute of Epidemiology, Helmholtz Zentrum München, German Research Center for Environmental Health, Neuherberg, Germany; 74 Bristol Dental School, University of Bristol, Lower Maudlin Street, Bristol, BS1 2LY UK; 75 Department of Genetic Medicine, Weill Cornell Medicine-Qatar, Doha, Qatar; 76 Department of Computer and Systems Engineering, Alexandria University, Alexandria, Egypt; 77 Population Health Sciences, Bristol Medical School, University of Bristol, Oakfield Grove, Bristol, BS8 2BN UK; 78 Saw Swee Hock School of Public Health, National University of Singapore and National University Health System, Singapore, 117549 Singapore; 79 Center for Clinical Research and Prevention, Bispebjerg and Frederiksberg Hospital, Copenhagen, Denmark; 80 Department of Clinical Medicine, Faculty of Health and Medical Sciences, University of Copenhagen, Copenhagen, Denmark; 81 The Institute for Translational Genomics and Population Sciences, Department of Pediatrics, Lundquist Institute for Biomedical Innovations (Formerly LABioMed) at Harbor-UCLA Medical Center, Torrance, CA 90502 USA; 82 Center for Public Health Genomics, University of Virginia, Charlottesville, VA 22903 USA; 83 Section of Endocrinology and Metabolism, Department of Medicine, Taipei Veterans General Hospital, Taipei, Taiwan; 84 Institute of Preventive Medicine, National Defense Medical Center, Postbox 90048~700, Sanhsia Dist, New Taipei City, 237101 Taiwan; 85 William Harvey Research Institute, Barts and The London School of Medicine and Dentistry, Queen Mary University of London, John Vane Science Centre, Charterhouse Square, London, EC1M 6BQ UK; 86 NIHR Barts Cardiovascular Biomedical Research Centre, Barts and The London School of Medicine and Dentistry, Queen Mary University of London, London, EC1M 6BQ UK; 87 Aragon Institute of Engineering Research, University of Zaragoza and Centro de Investigación Biomédica en Red - Bioingeniería, Biomateriales Y Nanomedicina, Spain; 88 Novo Nordisk Foundation Center for Basic Metabolic Research, Faculty of Health and Medical Sciences, University of Copenhagen, Copenhagen, Denmark; 89 Division of Cardiovascular Medicine, Radcliffe Department of Medicine, John Radcliffe Hospital, University of Oxford, Oxford, OX3 9DU UK; 90 Unit of Genomics of Complex Diseases, Institut d’Investigació Biomèdica Sant Pau (IIB SANT PAU), Barcelona, Spain; 91 Cardiovascular Medicine Unit, Department of Medicine, Karolinska Institutet, Center for Molecular Medicine, Karolinska University Hospital, Stockholm, Sweden; 92 Department of Internal Medicine, Section of Gerontology and Geriatrics, Leiden University Medical Center, Leiden, the Netherlands; 93 Istituto Di Ricerca Genetica E Biomedica, Consiglio Nazionale Delle Ricerche, Rome, Italy; 94 Dipartimento Di Scienze Biomediche, Università Degli Studi Di Sassari, Sardinia, Italy; 95 University Center for Primary Care and Public Health, University of Lausanne, Rte de Berne 113, 1010 Lausanne, Switzerland; 96 Swiss Institute of Bioinformatics, 1015 Lausanne, Switzerland; 97 Department of Medicine, Internal Medicine, Lausanne University Hospital and University of Lausanne, Rue du Bugnon 46, 1011 Lausanne, Switzerland; 98 Department of Epidemiology and Biostatistics, MRC-PHE Centre for Environment and Health, School of Public Health, Imperial College London, London, UK; 99 Department of Cardiology, Leiden University Medical Center, Leiden, the Netherlands; 100 Dept of Internal Medicine, Section of Gerontology and Geriatrics, Leiden University Medical Center, Leiden, the Netherlands; 101 BHF Glasgow Cardiovascular Research Centre, Faculty of Medicine, Glasgow, UK; 102 Institute for Cardiogenetics, University of Lübeck, DZHK (German Research Centre for Cardiovascular Research), Partner Site Hamburg/Lübeck/Kiel, University Heart Center Lübeck, Lübeck and Charité – University Medicine Berlin, corporate member of Freie Universität Berlin, Humboldt-Universität Zu Berlin and Berlin Institute of Health, Institute for Dental and Craniofacial Sciences, Department of Periodontology and Synoptic Dentistry, Berlin, Germany; 103 Deutsches Herzzentrum München, Klinik Für Herz- Und Kreislauferkrankungen, Technische Universität München, Munich, Germany; 104 Deutsches Zentrum Für Herz-Kreislauf-Forschung (DZHK) E.V., Partner Site Munich Heart Alliance, Munich, Germany; 105 Key Laboratory of Cardiovascular Epidemiology and Department of Epidemiology, State Key Laboratory of Cardiovascular Disease, Fuwai Hospital, National Center for Cardiovascular Diseases, Chinese Academy of Medical Sciences and Peking Union Medical College, Beijing, 100037 China; 106 Lund University Diabetes Center, Lund University, Malmö, Sweden; 107 Institute of Genetic Epidemiology, Department of Genetics, Medical University of Innsbruck, Innsbruck, Austria; 108 German Chronic Kidney Disease Study, Berlin, Germany; 109 Institute for Medical Informatics, Statistics and Epidemiology, University of Leipzig, Haertelstrasse 16-18, 04107 Leipzig, Germany; 110 LIFE Research Centre for Civilization Diseases, University of Leipzig, Philipp-Rosenthal-Straße 27, 04103 Leipzig, Germany; 111 Radboud University Medical Center, Radboud Institute for Health Sciences, Nijmegen, The Netherlands; 112 Quantinuum Research LLC, Wayne, PA 19087 USA; 113 Division of Statistical Genomics, Department of Genetics, Washington University School of Medicine, St. Louis, MO USA; 114 Department of Epidemiology, University of Pittsburgh, Pittsburgh, PA USA; 115 Institute for Biomedicine, Eurac Research, Affiliated Institute of the University of Lübeck, Via Galvani 31, 39100 Bolzano, Italy; 116 Department of Clinical Biochemistry, Lillebaelt Hospital, Vejle, Denmark; 117 Cardiovascular Health Research Unit, Department of Medicine, University of Washington, Seattle, 98101 USA; 118 Unidad de Investigacion Medica en Bioquimica, Hospital de Especialidades, Centro Medico Nacional Siglo XXI, Instituto Mexicano del Seguro Social, Mexico City, Mexico; 119 The Charles Bronfman Institute for Personalized Medicine, Icahn School of Medicine at Mount Sinai, New York, NY USA; 120 Department of Twin Research and Genetic Epidemiology, King’s College London, London, SE1 7EH UK; 121 NIHR Biomedical Research Centre at Guy’s and St Thomas’ Foundation Trust, London, SE1 9RT UK; 122 Department of Cardiology, University of Groningen, University Medical Center Groningen, Groningen, The Netherlands; 123 Institute of Cardiovascular Sciences, University College London, Gower Street, London, WC1E 6BT UK; 124 Department of Epidemiology and Public Health, University College London, 1-19 Torrington Place, London, WC1E 6BT UK; 125 Department of Surgery, University of Pennsylvania, Philadelphia, PA USA; 126 Amsterdam UMC, Department of Epidemiology and Data Science, Amsterdam Public Health Research Institute, Amsterdam, 1081HV the Netherlands; 127 Department of Cell and Chemical Biology, Leiden University Medical Center, Leiden, 2333ZA The Netherlands; 128 Montreal Heart Institute, Université de Montréal, 5000 Belanger Street, Montreal, PQ H1T1C8 Canada; 129 Icelandic Heart Association, 201 Kopavogur, Iceland; 130 Department of Anthropology, University of Toronto at Mississauga, Mississauga, ON L5L 1C6 Canada; 131 Vth Department of Medicine, Medical Faculty Mannheim, Heidelberg University, 68167 Mannheim, Germany; 132 SYNLAB MVZ Humangenetik Mannheim GmbH, 68163 Mannheim, Germany; 133 Shanghai Institute of Nutrition and Health, University of Chinese Academy of Sciences, Chinese Academy of Sciences, Shanghai, China; 134 Biomedical Technology Research Center, Tokushima Research Institute, Otsuka Pharmaceutical Co., Ltd, Tokushima, Japan; 135 School of Life Sciences, Westlake University, Hangzhou, 310024 Zhejiang China; 136 Westlake Laboratory of Life Sciences and Biomedicine, Hangzhou, 310024 Zhejiang China; 137 Institute for Molecular Bioscience, The University of Queensland, Brisbane, QLD 4072 Australia; 138 Nuffield Department of Population Health, University of Oxford, Oxford, UK; 139 Department of Epidemiology, Erasmus MC, University Medical Center Rotterdam, Rotterdam, the Netherlands; 140 Section of Statistical Multi-Omics, Department of Clinical and Experimental Research, University of Surrey, Guildford, Surrey UK; 141 Laboratory of Neurogenetics, National Institute On Aging, NIH, Bethesda, MD USA; 142 Data Tecnica International, Glen Echo, MD USA; 143 MRC Human Genetics Unit, Institute of Genetics and Cancer, University of Edinburgh, Western General Hospital, Crewe Road, Edinburgh, EH4 2XU Scotland; 144 Institute for Medical Informatics, Biometry and Epidemiology, University of Duisburg-Essen, Essen, Germany; 145 Centre for Public Health, Queen’s University of Belfast, Belfast, Northern Ireland; 146 Genomic Oncology Area, GENYO, Centre for Genomics and Oncological Research: Pfizer-University of Granada-Andalusian Regional Government, Granada, Spain; 147 Hematology Department, Hospital Universitario Virgen de Las Nieves, Granada, Spain; 148 Instituto de Investigación Biosanitaria de Granada (Ibs.GRANADA), Granada, Spain; 149 Department of Epidemiology and Biostatistics, School of Public Health, Tongji Medical College, Huazhong University of Science and Technology, Wuhan, China; 150 Genome Institute of Singapore, Agency for Science, Technology and Research, Singapore, Singapore; 151 Public Health Informatics Unit, Department of Integrated Health Sciences, Nagoya University Graduate School of Medicine, Nagoya, 461-8673 Japan; 152 MRC Unit for Lifelong Health and Ageing at UCL, 1-19 Torrington Place, London, WC1E 7HB UK; 153 Tampere Centre for Skills Training and Simulation, Faculty of Medicine and Health Technology, Tampere University, Tampere, Finland; 154 Brown Foundation Institute of Molecular Medicine, McGovern Medical School, University of Texas Health Science Center at Houston, Houston, TX 77030 USA; 155 CONACYT, Instituto Nacional de Ciencias Médicas Y Nutrición Salvador Zubirán, Ciudad de Mexico, Mexico; 156 Programs in Metabolism and Medical and Population Genetics, Broad Institute of MIT and Harvard, Cambridge, MA USA; 157 Departamento de Medicina Genómica Y Toxicología Ambiental, Instituto de Investigaciones Biomédicas, Universidad Nacional Autónoma de México, Coyoacán, 04510 Mexico City, Mexico; 158 Departamento de Genómica Computacional, Instituto Nacional de Medicina Genómica, Mexico City, Mexico; 159 Center for Diabetes Research, University of Bergen, Bergen, Norway; 160 Genomic Research On Complex Diseases (GRC Group), CSIR-Centre for Cellular and Molecular Biology, Hyderabad, Telangana India; 161 Academy of Scientific and Innovative Research (AcSIR), New Delhi, India; 162 Epidemiology, School of Public Health, University of Alabama at Birmingham, Birmingham, AL USA; 163 Hunter Medical Research Institute, Newcastle, Australia; 164 Medical Research Institute, Kangbuk Samsung Hospital, Sungkyunkwan University School of Medicine, Seoul, 03181 Korea; 165 Department of Clinical Research Design & Evaluation, SAIHST, Sungkyunkwan University, Seoul, 06355 Korea; 166 Center for Cohort Studies, Total Healthcare Center, Kangbuk Samsung Hospital, Sungkyunkwan University School of Medicine, Seoul, 04514 Korea; 167 Department of Occupational and Environmental Medicine, Kangbuk Samsung Hospital, Sungkyunkwan University School of Medicine, Seoul, 03181 Korea; 168 Centre for Global Health Research, Usher Institute, University of Edinburgh, Teviot Place, Edinburgh, EH8 9AG Scotland; 169 Thurston Arthritis Research Center, University of North Carolina, Chapel Hill, NC USA; 170 Duke-NUS Medical School, Health Services and Systems Research, Singapore, 169857 Singapore; 171 Division of Biomedical Informatics and Personalized Medicine, Department of Medicine, Anschutz Medical Campus, University of Colorado, Denver, Aurora, CO 80045 USA; 172 Genomics and Molecular Medicine Unit, CSIR-Institute of Genomics and Integrative Biology, New Delhi, 110020 India; 173 Academy of Scientific and Innovative Research, CSIR-Human Resource Development Centre, Ghaziabad, Uttar Pradesh India; 174 Departments of Ophthalmology and Human Genetics, Radboud University Nijmegen Medical Center, Philips Van Leydenlaan 15, Nijmegen, 6525 EX the Netherlands; 175 Vanderbilt Epidemiology Center, Division of Epidemiology, Vanderbilt University Medical Center, Nashville, USA; 176 Department of Pediatrics, University of California San Francisco, Oakland, CA 94609 USA; 177 National Center for Global Health and Medicine, Tokyo, 1628655 Japan; 178 Department of Genetics, University of North Carolina, Chapel Hill, NC 27599 USA; 179 Department of Biostatistics and Epidemiology, University of Massachusetts-Amherst, Amherst, MA 01003 USA; 180 Department of Cardiovascular Sciences, University of Leicester, Leicester, UK; 181 NIHR Leicester Biomedical Research Centre, Glenfield Hospital, Leicester, UK; 182 Beijing Institute of Ophthalmology, Beijing Key Laboratory of Ophthalmology and Visual Sciences, Beijing Tongren Eye Center, Beijing Tongren Hospital, Capital Medical University, 17 Hougou Lane, Chong Wen Men, Beijing, 100005 China; 183 Beijing Tongren Eye Center, Beijing Tongren Hospital, Capital Medical University, 1 Dong Jiao Min Xiang, Beijing, 100730 Dong Cheng District China; 184 Institute of Genetics and Biophysics “Adriano Buzzati-Traverso” - CNR, Naples, Italy; 185 IRCCS Neuromed, Pozzilli, Isernia Italy; 186 Division of Biostatistics, Washington University, St. Louis, MO 63110 USA; 187 Rush Alzheimer’s Disease Center, Rush University Medical Center, Chicago, IL USA; 188 Department of Neurological Sciences, Rush University Medical Center, Chicago, IL USA; 189 Dept of Nephrology, University Hospital Regensburg, Regensburg, Germany; 190 Institute for Maternal and Child Health, IRCCS Burlo Garofolo, Trieste, Italy; 191 Department of Molecular Epidemiology, German Institute of Human Nutrition Potsdam-Rehbruecke, Nuthetal, Germany; 192 German Center for Diabetes Research (DZD), Munich-Neuherberg, Germany; 193 Department of Genetics and Bioinformatics, Dasman Diabetes Institute, Kuwait City, Kuwait; 194 Department of Nutrition and Dietetics, School of Health Science and Education, Harokopio University of Athens, Athens, Greece; 195 Department of Clinical Epidemiology, Institute of Health Informatics, University College London, London, UK; 196 Clinical Division of Neurogeriatrics, Department of Neurology, Medical University of Graz, Graz, Austria; 197 Institute for Medical Informatics, Statistics and Documentation, Medical University of Graz, Graz, Austria; 198 Massachusetts General Hospital Cancer Center, Charlestown, MA 02129 USA; 199 Institute of Genetic and Biomedical Research, National Research Council of Italy, UOS of Sassari, Sassari, Italy; 200 Department of Epidemiology, University of Groningen, University Medical Center Groningen, Groningen, 9700 RB the Netherlands; 201 Research Centre of Applied and Preventive Cardiovascular Medicine, University of Turku, Turku, Finland; 202 Centre for Population Health Research, University of Turku and Turku University Hospital, Turku, Finland; 203 Sleep Medicine and Circadian Disorders, Brigham and Women’s Hospital, Boston, MA 02115 USA; 204 Division of Sleep Medicine, Harvard Medical School, Boston, MA 02115 USA; 205 Central Diagnostics Laboratory, Division Laboratories, Pharmacy, and Biomedical Genetics, University Medical Center Utrecht, Utrecht University, Utrecht, the Netherlands; 206 Laboratory of Epidemiology and Population Sciences, National Institute On Aging, NIH, Baltimore, MD 20892-9205 USA; 207 Department of Engineering Technology, University of Houston-Sugarland, Houston, TX USA; 208 Interfaculty Institute for Genetics and Functional Genomics, Department of Functional Genomics, University of Greifswald and University Medicine Greifswald, Greifswald, Germany; 209 Center for Research On Genomics and Global Health, National Human Genome Research Institute, National Institutes of Health, 12 South Drive, Room 1025, Bethesda, MD 20892 USA; 210 Oneomics. Co. Ltd. 2F, Soonchunhyang Mirai Medical Center 173, Buheuyng-Ro, Bucheon-Si Gyeonggi-Do, 14585 Korea; 211 Department of Clinical Biochemistry and Immunology, Hospital of Southern Jutland, Kresten Philipsens Vej 15, 6200 Aabenraa, Denmark; 212 Department of Clinical Biochemistry, Lillebaelt Hospital, Kolding, Denmark; 213 Department of Biomedical Science, Hallym University, Chuncheon, 24252 Gangwon-Do Korea; 214 Centre for Bone and Arthritis Research, Department of Internal Medicine and Clinical Nutrition, Institute of Medicine, Sahlgrenska Academy, University of Gothenburg, Gothenburg, Sweden; 215 Bioinformatics Core Facility, Sahlgrenska Academy, University of Gothenburg, Gothenburg, Sweden; 216 Institute of Medical Informatics and Statistics, Kiel University, Kiel, Germany; 217 Institute of Translational Genomics, Helmholtz Zentrum München – German Research Center for Environmental Health, Neuherberg, Germany; 218 Oxford Centre for Diabetes, Endocrinology and Metabolism, University of Oxford, Oxford, UK; 219 School of Medicine and Public Health, College of Health, Medicine and Wellbeing, University of Newcastle, Newcastle, NSW 2308 Australia; 220 Center for Geriatrics and Gerontology, Division of Endocrinology and Metabolism, Department of Internal Medicine, Taichung Veterans General Hospital, Taichung, Taiwan; 221 School of Medicine, National Yang-Ming University, Taipei, Taiwan; 222 School of Medicine, National Defense Medical Center, Taipei, Taiwan; 223 Division of Endocrinology and Metabolism, Department of Internal Medicine, Taichung Veterans General Hospital, Taichung, Taiwan; 224 Department of Medicine, School of Medicine, National Yang Ming Chiao Tung University, Taipei, Taiwan; 225 Department of Kinesiology, Université Laval, Québec, Canada; 226 Department of Clinical Chemistry, Fimlab Laboratories, 33520 Tampere, Finland; 227 Department of Clinical Chemistry, Finnish Cardiovascular Research Center - Tampere, Faculty of Medicine and Health Technology, Tampere University, 33014 Tampere, Finland; 228 Department of Cardiology, Heart Center, Tampere University Hospital, 33521 Tampere, Finland; 229 Department of Cardiology, Finnish Cardiovascular Research Center - Tampere, Faculty of Medicine and Health Technology, Tampere University, 33014 Tampere, Finland; 230 University of Queensland Diamantina Institute, Translational Research Institute, Kent St, Woolloongabba, Brisbane, QLD 4102 Australia; 231 Department of Medicine, Bornholms Hospital, Rønne, Denmark; 232 Department of Epidemiology, Ryals School of Public Health, University of Alabama at Birmingham, Birmingham, AL USA; 233 Cardiology, Division Heart and Lungs, University Medical Center Utrecht, Utrecht University, Utrecht, the Netherlands; 234 Department of Population and Quantitative Health Sciences, Case Western Reserve University, Cleveland, OH 44106 USA; 235 Department of Genetics, Stanford University School of Medicine Stanford, Palo Alto, CA 94305 USA; 236 Division of Endocrinology, Ohio State University, Columbus, OH 43210 USA; 237 Department of Epidemiology, University of Washington, Seattle, WA 98195 USA; 238 School of Medicine and Health Sciences, George Washington University, Washington, DC 20037 USA; 239 Department of Epidemiology, School of Public Health, Peking University Health Science Center, Beijing, China; 240 Institute for Laboratory Medicine, University Hospital Leipzig, Paul-List-Strasse 13/15, 04103 Leipzig, Germany; 241 Laboratory of Epidemiology and Population Sciences, National Institute On Aging, NIH, Baltimore, MD 20892-9205 USA; 242 Center for Alzheimer’s and Related Dementias, NIH, Bethesda, MD USA; 243 Data Tecnica International, Washington, DC USA; 244 Department of Clinical Physiology and Nuclear Medicine, Turku University Hospital, Turku, Finland; 245 Department of Environmental and Preventive Medicine, Jichi Medical University School of Medicine, Shimotsuke, 329-0498 Japan; 246 Centre for Population Health Sciences, Usher Institute, University of Edinburgh, Teviot Place, Edinburgh, EH8 9AG Scotland; 247 Department of Functional Pathology, Shimane University School of Medicine, Izumo, 6938501 Japan; 248 Department of Pediatrics and Adolescent Medicine, Turku University Hospital and University of Turku, Turku, Finland; 249 Department of Physiology, University of Turku, Turku, Finland; 250 University of Split School of Medicine, Šoltanska 2, HR-21000 Split, Croatia; 251 University of Leipzig Medical Center, Liebigstr. 18, 04103 Medical Department III – Endocrinology, Nephrology, RheumatologyLeipzig, Germany; 252 Department of Nutrition-Dietetics, Harokopio University, Eleftheriou Venizelou, 17676 Athens, Greece; 253 Department of Clinical Gene Therapy, Osaka University Graduate School of Medicine, Suita, 5650871 Japan; 254 Department of Geriatric and General Medicine, Osaka University Graduate School of Medicine, Suita, 5650871 Japan; 255 Department of Vascular Surgery, Division of Surgical Specialties, University Medical Center Utrecht, Utrecht University, Utrecht, the Netherlands; 256 Corneal Dystrophy Research Institute, Yonsei University College of Medicine, Saevit Eye Hospital, SeoulIlsan, 03722 Korea; 257 Dept of Radiology and Nuclear Medicine, Erasmus MC, University Medical Center Rotterdam, Rotterdam, the Netherlands; 258 Latin American Brain Health (BrainLat), Universidad Adolfo Ibáñez, Santiago, Chile; 259 Julius Centre for Health Sciences and Primary Care, University Medical Centre Utrecht, 3584CG Utrecht, the Netherlands; 260 Second Department of Cardiology, Medical School, National and Kapodistrian University of Athens, Attikon University Hospital, Athens, Greece; 261 Center for Vision Research, Department of Ophthalmology and The Westmead Institute, University of Sydney, Hawkesbury Rd, Sydney, NSW 2145 Australia; 262 School of Medicine, Menzies Institute for Medical Research, University of Tasmania, Liverpool St, Hobart, TAS 7000 Australia; 263 Centre for Eye Research Australia, University of Melbourne, Melbourne, VIC 3002 Australia; 264 Department of Clinical Physiology, Tampere University Hospital, 33521 Tampere, Finland; 265 Department of Clinical Physiology, Finnish Cardiovascular Research Center - Tampere, Faculty of Medicine and Health Technology, Tampere University, 33014 Tampere, Finland; 266 Centre Nutrition, Santé Et Société (NUTRISS), Institute of Nutrition and Functional Foods (INAF), Québec, Canada; 267 Pennington Biomedical Research Center, Baton Rouge, LA 70808 USA; 268 Discipline of Internal Medicine, Medical School, The University of Western Australia, Perth, WA Australia; 269 Institute of Epidemiology, Kiel University, Kiel, Germany; 270 Institute of Clinical Molecular Biology, Kiel University, Kiel, Germany; 271 Department of Drug Treatment, Sahlgrenska University Hospital, Gothenburg, Sweden; 272 Geriatric Medicine, Institute of Medicine, Sahlgrenska Academy, University of Gothenburg, Gothenburg, Sweden; 273 Department of Internal Medicine, EwhaWomans University School of Medicine, Seoul, Korea; 274 Division of Cancer Control and Population Sciences, UPMC Hillman Cancer Center, University of Pittsburgh, Pittsburgh, PA 15232 USA; 275 Department of Epidemiology, Graduate School of Public Health, University of Pittsburgh, Pittsburgh, PA 15232 USA; 276 Healthy Longevity Translational Research Programme, Yong Loo Lin School of Medicine, National University of Singapore, Singapore, 117545 Singapore; 277 Singapore Institute for Clinical Sciences, Agency for Science Technology and Research (A*STAR), Singapore, 117609 Singapore; 278 Department of Endocrinology and Metabolism, Kyung Hee University School of Medicine, Seoul, 02447 Korea; 279 Institute for Community Medicine, University Medicine Greifswald, Greifswald, Germany; 280 Laboratory of Epidemiology and Population Science, National Institute On Aging Intramural Research Program, NIH Biomedical Research Center, NIH 251 Bayview Blvd, Baltimore, MD 21224 USA; 281 Algebra University College, Ilica 242, Zagreb, Croatia; 282 Department of Physical Activity and Health, Paavo Nurmi Centre, Sports and Exercise Medicine Unit, University of Turku, Turku, Finland; 283 Interdisciplinary Center Psychopathology and Emotion Regulation (ICPE), University of Groningen, University Medical Center Groningen, Groningen, 9700 RB the Netherlands; 284 Institute of Molecular Genetics, National Research Council of Italy, Pavia, Italy; 285 Gottfried Schatz Research Center for Cell Signaling, Metabolism and Aging, Medical University of Graz, Graz, Austria; 286 Local Health Unit Toscana Centro, Florence, Italy; 287 Institute of Nutritional Science, University of Potsdam, Nuthetal, Germany; 288 Department of Medicine, Surgery and Health Sciences, University of Trieste, Strada Di Fiume 447, 34149 Trieste, Italy; 289 Department of Nephrology, , Traunstein Hospital, Diabetology, RheumatologyTraunstein, Germany; 290 KfH Kidney Center Traunstein, Traunstein, Germany; 291 Department of Neurology, Center for Translational and Systems Neuroimmunology, Columbia University Medical Center, New York, NY USA; 292 Medical School, National and Kapodistrian University Athens, 75 M. Assias Street, 115 27 Athens, Greece; 293 Dromokaiteio Psychiatric Hospital, 124 61 Athens, Greece; 294 Clinical Pharmacology, William Harvey Research Institute, Queen Mary University of London, London, EC1M 6BQ UK; 295 Department of Ophthalmology, Medical Faculty Mannheim, Heidelberg University, Kutzerufer 1, 68167 Mannheim, Germany; 296 Institute of Molecular and Clinical Ophthalmology, Basel, Switzerland; 297 Privatpraxis Prof Jonas Und Dr Panda-Jonas, Heidelberg, Germany; 298 Department of Human Genetics, David Geffen School of Medicine at UCLA, University of California, Los Angeles, CA USA; 299 Unidad de Biología Molecular Y Medicina Genómica, Instituto de Investigaciones Biomédicas UNAM/ Instituto Nacional de Ciencias Médicas Y Nutrición Salvador Zubirán, Mexico City, Mexico; 300 Departamento de Endocrinología Y Metabolismo, Instituto Nacional de Ciencias Médicas Y Nutrición Salvador Zubirán, 14080 Mexico, Mexico; 301 Department of Nutrition, Gillings School of Global Public Health, University of North Carolina, Chapel Hill, NC 27599 USA; 302 Carolina Population Center, University of North Carolina, Chapel Hill, NC 27516 USA; 303 USC–Office of Population Studies Foundation, University of San Carlos, 6000 Cebu City, Philippines; 304 Department of Anthropology, Sociology, and History, University of San Carlos, 6000 Cebu City, Philippines; 305 Department of Medicine, Faculty of Medicine, University of Kelaniya, Ragama, 11010 Sri Lanka; 306 Department of Public Health, Faculty of Medicine, University of Kelaniya, Ragama, 11010 Sri Lanka; 307 Children’s Hospital Oakland Research Institute, Oakland, CA 94609 USA; 308 Institute of Biomedical Sciences, Academia Sinica, Taipei, Taiwan; 309 Systems Genomics Laboratory, School of Biotechnology, Jawaharlal Nehru University, New Delhi, 110067 India; 310 Department of Medicine, University of Mississippi Medical Center, Jackson, MS 39216 USA; 311 Department of Physiology and Biophysics, University of Mississippi Medical Center, Jackson, MS 39216 USA; 312 Department of Medical Sciences, Uppsala University, Uppsala, Sweden; 313 Department of Paediatrics, Yong Loo Lin School of Medicine, National University of Singapore and Khoo Teck Puat - National University Children’s Medical Institute, National University Health System, Singapore, Singapore; 314 Department of Medicine, University of North Carolina, Chapel Hill, NC USA; 315 Injury Prevention Research Center, University of North Carolina, Chapel Hill, NC USA; 316 Division of Physical Therapy, University of North Carolina, Chapel Hill, NC USA; 317 Department of Psychiatry, Amsterdam UMC, Vrije Universiteit Amsterdam, Amsterdam, the Netherlands; 318 Department of Biochemistry, College of Medicine, Ewha Womans University, Seoul, 07804 Korea; 319 Faculty of Health and Medicine, University of Newcastle, Callaghan, Australia; 320 Division of Biostatistics, Washington University School of Medicine, St. Louis, USA; 321 Office of the Provost, University of South Carolina, Columbia, SC USA; 322 Department of Internal Medicine, University of Utah, Salt Lake City, Utah 84132 USA; 323 Institute of Cellular Medicine (Diabetes), The Medical School, Newcastle University, Framlington Place, Newcastle Upon Tyne, NE2 4HH UK; 324 Department of Medicine, Helsinki University Hospital, University of Helsinki, Haartmaninkatu 4, P.O.Box 340, 00029 Helsinki, Finland; 325 Minerva Foundation Institute for Medical Research, Biomedicum 2U, Tukholmankatu 8, 00290 Helsinki, Finland; 326 JSS Academy of Higher Education and Research, Mysuru, India; 327 Diabetes Unit and Center for Genomic Medicine, Massachusetts General Hospital, Boston, MA USA; 328 Harvard Medical School, Boston, MA 02115 USA; 329 InstitutoNacional de Salud Publica Y Centro de Estudios en Diabetes, Cuernavaca, Mexico; 330 Laboratorio de Inmunogenómica Y Enfermedades Metabólicas, Instituto Nacional de Medicina Genómica, Mexico City, Mexico; 331 Human Genetics Center, School of Public Health, University of Texas Health Science Center at Houston, Houston, TX 77030 USA; 332 Yong Loo Lin School of Medicine, National University of Singapore and National University Health System, Singapore, 119228 Singapore; 333 Department of Exercise and Nutrition Sciences, Milken Institute School of Public Health, George Washington University, Washington, DC 20052 USA; 334 Kurume University School of Medicine, Kurume, 830-0011 Japan; 335 Genetics, Merck Sharp & Dohme Corp., Kenilworth, NJ 07033 USA; 336 Population Health and Genomics, University of Dundee, Ninwells Hospital and Medical School, Dundee, DD1 9SY UK; 337 Intramural Research Program, National Institute On Aging, 3001 S. Hanover St., Baltimore, MD 21225 USA; 338 Beijing Institute of Ophthalmology, Beijing Tongren Eye Center, Beijing Tongren Hospital, Capital Medical University, Beijing Ophthalmology and Visual Sciences Key Laboratory, Beijing, 100730 China; 339 The Eye Hospital, School of Ophthalmology and Optometry, Wenzhou Medical University, Wenzhou, 325027 Zhejiang China; 340 Synlab Academy, SYNLAB Holding Deutschland GmbH, Mannheim and Augsburg, Germany; 341 Clinical Institute of Medical and Chemical Laboratory Diagnostics, Medical University of Graz, Graz, Austria; 342 Faculty of Medicine, University of Iceland, 101 Reykjavik, Iceland; 343 Department of Biomedical Data Sciences, Section Molecular Epidemiology, Leiden University Medical Center, Leiden, 2333ZA The Netherlands; 344 Department of Epidemiology and Data Science, Amsterdam UMC, Amsterdam, 1081HV the Netherlands; 345 Amsterdam Public Health Research Institute, Amsterdam Cardiovascular Sciences, Amsterdam, 1081HV the Netherlands; 346 Department of General Practice, Amsterdam UMC, Amsterdam, 1081HV the Netherlands; 347 Amsterdam Public Health Research Institute, Health Behaviours and Chronic Diseases, Amsterdam, 1081HV the Netherlands; 348 Corporal Michael Crescenz VA Medical Center, Philadelphia, PA 19104 USA; 349 Institute of Social and Economic Research, University of Essex, Wivenhoe Park, Colchester, CO4 3SQ UK; 350 Department of Environmental Medicine and Public Health, Icahn School of Medicine at Mount Sinai, New York, NY USA; 351 Department of Epidemiology, University of Washington, Seattle, WA USA; 352 Department of Health Systems and Population Health, University of Washington, Seattle, WA USA; 353 Institute of Regional Health Research, University of Southern Denmark, Odense, Denmark; 354 Danish Aging Research Center, University of Southern Denmark, Odense C, Denmark; 355 Public Health, Faculty of Medicine, University of Helsinki, Helsinki, Finland; 356 Broad Institute of MIT and Harvard, Cambridge, MA USA; 357 Center for Applied Genomics, Children’s Hospital of Philadelphia, Philadelphia, PA 19104 USA; 358 Department of Pediatrics, The University of Pennsylvania Perelman School of Medicine, Philadelphia, PA 19104 USA; 359 Division of Human Genetics, Children’s Hospital of Philadelphia, Philadelphia, PA 19104 USA; 360 School of Medicine, Southern University of Science and Technology, Shenzhen, China; 361 Institute for Cardiogenetics, University of Lübeck, DZHK (German Research Centre for Cardiovascular Research), Partner Site Hamburg/Lübeck/Kiel, and University Heart Center Lübeck, Lübeck, Germany; 362 Netherlands Heart Institute, Utrecht, the Netherlands; 363 Division of Cardiology, Department of Medicine, Massachusetts General Hospital, Boston, MA USA; 364 Department of Medicine, Center for Genomic Medicine, Massachusetts General Hospital, Boston, MA USA; 365 Department of Medicine, Harvard Medical School, Boston, MA USA; 366 Verve Therapeutics, Cambridge, MA USA; 367 Northern Finland Birth Cohorts, Infrastructure for Population Studies, Faculty of Medicine, University of Oulu, Oulu, Finland; 368 Center for Life Course Health Research, Faculty of Medicine, University of Oulu, Oulu, Finland; 369 Biocenter of Oulu, University of Oulu, Oulu, Finland; 370 Institute for Genetic and Biomedical Research, Italian National Council of Research (IRGB CNR), Cagliari, Italy; 371 University of Sassari, Sassari, Italy; 372 Department of Clinical Epidemiology, Leiden University Medical Center, Leiden, the Netherlands; 373 Department of Public Health and Primary Care, Leiden University Medical Center, Leiden, the Netherlands; 374 Department of Internal Medicine, Division of Endocrinology, Leiden University Medical Center, Leiden, the Netherlands; 375 Einthoven Laboratory for Experimental Vascular Medicine, Leiden University Medical Center, Leiden, the Netherlands; 376 Department of Human Genetics, Leiden University Medical Center, Leiden, the Netherlands; 377 Population Health Research Institute, St George’s, University of London, London, SW17 0RE UK; 378 National Heart and Lung Institute, Imperial College London, London, W12 0NN UK; 379 School of Public Health, Imperial College London, London, W12 7RH UK; 380 Department of Internal Medicine, National Taiwan University Hospital, No. 7, Chung-Shan South Road, Taipei, Taiwan; 381 OCDEM, University of Oxford, Churchill Hospital, Oxford, OX3 7LE UK; 382 NIHR Oxford Biomedical Research Centre, Churchill Hospital, Oxford, UK; 383 Ocular Epidemiology, Singapore Eye Research Institute, Singapore National Eye Centre, Singapore, 168751 Singapore; 384 Ophthalmology and Visual Sciences Academic Clinical Program (Eye ACP), Duke-NUS Medical School, Singapore, 169857 Singapore; 385 Data Science, Singapore Eye Research Institute, Singapore National Eye Centre, Singapore, 168751 Singapore; 386 Medical School, University of Exeter, University of Exeter, Exeter, EX2 5DW UK; 387 Department of Medical Epidemiology and Biostatistics, Karolinska Institutet, Stockholm, Sweden; 388 Framingham Heart Study, National Heart, Lung, and Blood Institute, US National Institutes of Health, Bethesda, MD USA; 389 Department of Genetics, School of Medicine, Mashhad University of Medical Sciences, Mashhad, Iran; 390 Department of Genetics, Shanghai-MOST Key Laboratory of Health and Disease Genomics, Chinese National Human Genome Center at Shanghai, Shanghai, 201203 China; 391 Computational Medicine, Berlin Institute of Health at Charité – Universitätsmedizin, Berlin, Germany; 392 Technical University of Munich (TUM) and Klinikum Rechts Der Isar, TUM School of Medicine, Munich, Germany; 393 Institute of Clinical Medicine, Internal Medicine, University of Eastern Finland and Kuopio University Hospital, Kuopio, Finland; 394 Stanford Cardiovascular Institute, Stanford University, Stanford, CA 94305 USA; 395 Stanford Diabetes Research Center, Stanford University, Stanford, CA 94305 USA; 396 Regeneron Pharmaceuticals, Tarrytown, NY USA; 397 Lee Kong Chian School of Medicine, Nanyang Technological University, Singapore, 308232 Singapore; 398 Imperial College Healthcare NHS Trust, Imperial College London, London, W12 0HS UK; 399 Imperial College Healthcare NHS Trust, London, W12 0HS UK; 400 MRC-PHE Centre for Environment and Health, Imperial College London, London, W2 1PG UK; 401 School of Electrical and Information Engineering, University of the Witwatersrand, Johannesburg, South Africa; 402 Institute for Minority Health Research, University of Illinois College of Medicine, Chicago, IL USA; 403 Department of Biostatistics, Harvard T.H. Chan School of Public Health, 677 Huntington Avenue, Boston, MA 02115 USA; 404 QIMR Berghofer Medical Research Institute, 300 Herston Road, Brisbane, QLD 4006 Australia; 405 Faisalabad Institute of Cardiology, Faislabad, Pakistan; 406 Department of Medicine, Columbia University Irving Medical Center, New York, NY USA; 407 Department of Cardiology, Columbia University Irving Medical Center, New York, NY USA; 408 Big Data Institute, University of Oxford, Oxford, OX3 7LF UK; 409 National Institute for Health Research Oxford Biomedical Research Centre, Oxford University Hospitals, Oxford, UK; 410 Aberdeen Centre for Health Data Science,1:042 Polwarth Building School of Medicine, Medical Science and Nutrition University of Aberdeen, Foresterhill, Aberdeen, AB25 2ZD UK; 411 Division of Population Health and Genomics, Ninewells Hospital and Medical School, University of Dundee, Dundee, DD1 9SY UK; 412 Department of Population Health Sciences, Geisinger Health, Danville, PA 17822 USA; 413 School of Basic and Medical Biosciences, Faculty of Life Sciences and Medicine, King’s College London, London, UK; 414 Department of Biomedical Informatics, Vanderbilt University Medical Center, Nashville, TN USA; 415 Departments of Medicine (Medical Genetics) and Genome Sciences, University of Washington, Washington, USA; 416 Center for Autoimmune Genomics and Etiology, Cincinnati Children’s Hospital Medical Center (CCHMC), Cincinnati, OH USA; 417 Division of Endocrinology, Metabolism, and Molecular Medicine, Department of Medicine, Northwestern University, Feinberg School of Medicine, Chicago, IL 60618 USA; 418 Department of Anthropology, Northwestern University, Evanston, IL 60208 USA; 419 Department of Public Health and Nursing, HUNT Research Centre, NTNU, Norwegian University of Science and Technology, 7600 Levanger, Norway; 420 Department of Research, St. Olavs Hospital, Trondheim University Hospital, Trondheim, Norway; 421 Department of Endocrinology, St. Olavs Hospital, Trondheim University Hospital, Trondheim, Norway; 422 RIKEN Center for Integrative Medical Sciences, Kanagawa, Japan; 423 Laboratory for Statistical Analysis, RIKEN Center for Integrative Medical Sciences, Kanagawa, Japan; 424 Laboratory of Complex Trait Genomics, Department of Computational Biology and Medical Sciences, Graduate School of Frontier Sciences, The University of Tokyo, Tokyo, Japan; 425 Laboratory for Systems Genetics, RIKEN Center for Integrative Medical Sciences, Kanagawa, Japan; 426 Department of Genome Informatics, Graduate School of Medicine, the University of Tokyo, Tokyo, Japan; 427 Division of Molecular Pathology, Institute of Medical Science, The University of Tokyo, Tokyo, Japan; 428 Faculty of Medicine, University of Iceland, Sæmundargötu 2, Reykjavik, 102 Iceland; 429 VA Boston Healthcare System, Boston, MA USA; 430 VA Informatics and Computing Infrastructure, VA Salt Lake City Health Care System, Salt Lake City, UT USA; 431 University of Massachusetts, Boston, MA USA; 432 Cardiovascular Institute, Stanford University School of Medicine, Stanford, CA USA; 433 Corporal Michael J. Crescenz VA Medical Center, Philadelphia, PA USA; 434 Department of Medicine, University of Pennsylvania Perelman School of Medicine, Philadelphia, PA USA; 435 Department of Medicine, Brigham Women’s Hospital, Boston, MA USA; 436 Division of Cardiology, Emory University School of Medicine, Atlanta, GA USA; 437 Departments of Pediatrics and Genetics, Harvard Medical School, Boston, MA USA; 438 Centre for Genetics and Genomics Versus Arthritis, Centre for Musculoskeletal Research, Division of Musculoskeletal and Dermatological Sciences, The University of Manchester, Manchester, UK; 439 Cardiology Division, Massachusetts General Hospital, Harvard Medical School, Boston, MA USA; 440 Department of Medicine, Massachusetts General Hospital, Harvard Medical School, Boston, MA USA; 441 Cardiovascular Research Center and Center for Genomic Medicine, Massachusetts General Hospital, Boston, MA USA; 442 Princess Al-Jawhara Al-Brahim Centre of Excellence in Research of Hereditary Disorders (PACER-HD), King Abdulaziz University, Jeddah, Saudi Arabia; 443 Department of Human Genetics, University of Michigan, Ann Arbor, MI 48109 USA

**Keywords:** Cholesterol, Lipids, Genetics, Genome-wide association study, GWAS

## Abstract

**Background:**

Genetic variants within nearly 1000 loci are known to contribute to modulation of blood lipid levels. However, the biological pathways underlying these associations are frequently unknown, limiting understanding of these findings and hindering downstream translational efforts such as drug target discovery.

**Results:**

To expand our understanding of the underlying biological pathways and mechanisms controlling blood lipid levels, we leverage a large multi-ancestry meta-analysis (*N* = 1,654,960) of blood lipids to prioritize putative causal genes for 2286 lipid associations using six gene prediction approaches. Using phenome-wide association (PheWAS) scans, we identify relationships of genetically predicted lipid levels to other diseases and conditions. We confirm known pleiotropic associations with cardiovascular phenotypes and determine novel associations, notably with cholelithiasis risk. We perform sex-stratified GWAS meta-analysis of lipid levels and show that 3–5% of autosomal lipid-associated loci demonstrate sex-biased effects. Finally, we report 21 novel lipid loci identified on the X chromosome. Many of the sex-biased autosomal and X chromosome lipid loci show pleiotropic associations with sex hormones, emphasizing the role of hormone regulation in lipid metabolism.

**Conclusions:**

Taken together, our findings provide insights into the biological mechanisms through which associated variants lead to altered lipid levels and potentially cardiovascular disease risk.

**Supplementary Information:**

The online version contains supplementary material available at 10.1186/s13059-022-02837-1.

## Background

Abnormal blood lipid levels are a major cause of cardiovascular disease [1], the leading cause of morbidity and mortality worldwide [2]. Conventional blood lipid measures, low-density lipoprotein cholesterol (LDL-C), total cholesterol (TC), triglyceride (TG), high-density lipoprotein cholesterol (HDL-C), and nonHDL-C (TC – HDL-C), are commonly used in clinical practice to identify individuals at high risk for cardiovascular events. Several treatments for reducing LDL-C, including statins, ezetimibe, and PCSK9 inhibitors [3], also reduce the risk of developing cardiovascular disease.

Genome-wide association studies (GWAS) for blood lipids have identified nearly 1000 associated genetic loci to date [4–23], including our recent multi-ancestry GWAS meta-analysis in 1.65 M individuals [24]. The latter focused on the gains from the multi-ancestry meta-analysis relative to the single-ancestry results, in terms of number of loci, fine-mapping, and polygenic score (PGS) transferability. However, a challenge in the field is that the underlying gene and biological pathways is often unknown for GWAS loci. Within lipid GWAS, prior fine-mapping studies combined with functional follow-up have successfully identified causal genes with high confidence for only a handful of associated GWAS loci, including *SORT1* [25], *TM6SF2* [12], and *ANGPTL3* [26], among others. Highly sophisticated methods are emerging to prioritize causal genes in well-powered GWAS studies, such as the Data-driven Expression-Prioritized Integration for Complex Traits [27] (DEPICT) and the Polygenic Priority Score [28] (PoPS), that take into account genome-wide properties of associated loci and larger sets of associated loci are beneficial. These methods can be combined with algorithms that integrate expression data such as transcriptome-wide association studies (TWAS) and comprehensive experimental data sets such as mouse gene knockouts. Gene sets enriched for causal genes will enhance our ability to unravel the biological pathways underlying these associations and there is growing interest in using a combination of gene prioritization methods to provide compelling evidence for putative causal genes [29].

In parallel, the linkage of electronic health records with genetic data in large-scale population studies and patient biobanks allows for the systematic exploration of pleiotropy of lipid-associated alleles. While blood lipid levels have a well-documented causal effect on cardiovascular disease based on genetic association studies validated by randomized controlled trials [30–32], genetic pleiotropic associations might exist for other conditions. Unraveling such pleiotropy may yield new biological insights by revealing previously unrecognized connections between blood lipids and both cardiovascular and non-cardiovascular diseases. Phenome-wide association scans (PheWAS) adopt an agnostic approach to test for pleiotropic associations between genetic factors and a wide range of phenotypes [33]. Such knowledge may allow for the identification of lipid levels as novel diagnostic biomarkers, the repurposing of drugs, and the prevention of adverse drug events [34].

Finally, given empirical sex differences in blood lipid distributions, sex-specific genetic associations may yield novel biological insights. Pre-menopausal females have lower levels of LDL-C than same-age males, and HDL-C levels are higher among females of all ages compared to males [35]. Lipid levels also show a greater estimated heritability in females compared with males [36], especially for LDL-C and TC (> 1.3-fold difference). Sexual dimorphism in lipid levels may be partly explained by X chromosome variants. Evidence from human X-linked abnormalities (like Turner or Klinefelter syndromes) suggests an important role of this chromosome in lipid metabolism [37]. This is further corroborated by the lipid and atherosclerosis profiles in the Four Core Genotypes mouse model [38], which comprises XX and XY gonadal males and XX and XY gonadal females. GWAS studies have traditionally understudied the X chromosome due to technical and analytical difficulties. A recent high coverage whole X chromosome sequencing study [39] prioritized *CHRDL1* as a candidate causal lipid gene, suggesting with larger sample sizes we may be able to discover additional variation on the X chromosome associated with lipid levels.

In this study, we first prioritize genes at GWAS lipid loci through multiple in silico gene prediction algorithms and experimental data sources using the latest Global Lipids Genetics Consortium multi-ancestry meta-analysis [24]. We then identify novel disease associations related to lipid levels through PheWAS in two large biobanks using PGSs. Finally, we perform sex-stratified meta-analysis to compare the associations between males and females to identify genetic loci with sex-specific associations and GWAS meta-analysis of the X chromosome, to better understand lipid level differences between the sexes. Together, our results highlight biological mechanisms through which lipid-associated variants lead to altered lipid levels.

## Results

### Identifying functional genes in lipid-associated loci

In a GWAS meta-analysis of blood lipid levels from 1.65 million individuals (Additional file 1: Table S1) at 91 million genotyped and imputed genetic variants, we observed a total of 2286 genome-wide significant index variants associated with lipid levels at 923 loci (± 500 kb regions). This corresponded to 416 index variants associated with LDL-C, 539 with HDL-C, 461 with TG, 487 with TC, and 383 with nonHDL-C. Uniquely, we observed 1750 variants associated with one or more lipid levels [24] (Additional file 2: Table S2).

**Fig. 1:**
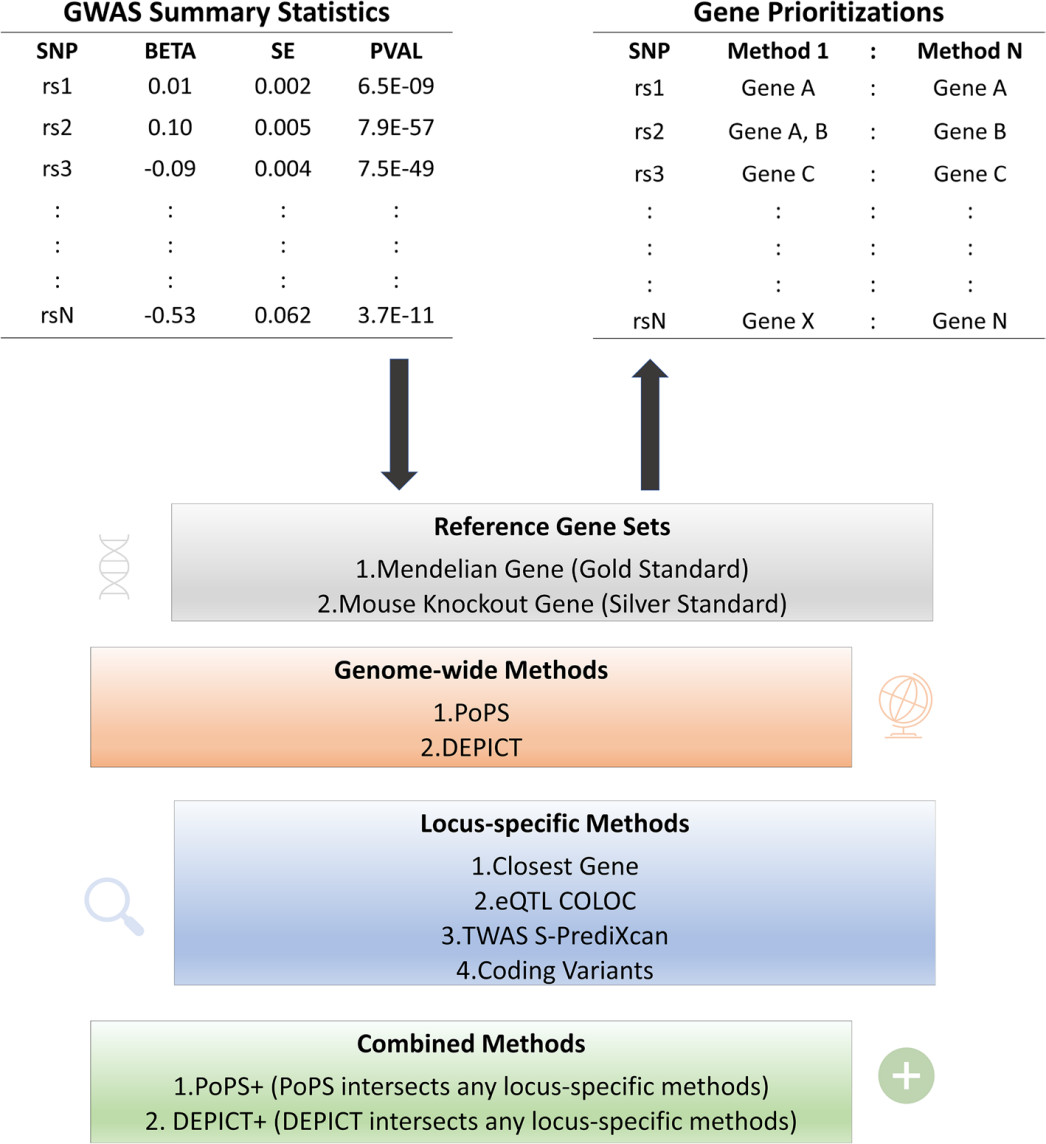
Schematic of multi-method candidate gene mapping of indexed variants associated with blood lipid levels. We defined indexed variants within the GLGC GWAS summary statistics and performed two similarity-based methods and four locus-based methods to prioritize genes for each of the indexed variants

We employed six approaches to identify candidate functional genes for all 2286 lipid associations. Our prioritization approaches include four locus-specific methods that are based on local information around the indexed variant: (1) the closest gene to the index variant, (2) genes with lipid-associated protein-altering variants, (3) colocalized expression quantitative trait loci (eQTL) genes, and (4) nearby genes prioritized by transcript-wide association studies (TWAS). We also used two genome-wide methods that leveraged genome-wide similarities of features: (1) gene-level Polygenic Priority Score (PoPS) [28] and (2) Data-driven Expression-Prioritized Integration for Complex Traits (DEPICT) [27]. We further combined the two genome-wide methods with the locus-specific methods to increase the confidence in prioritized genes: (1) PoPS intersects with any locus-specific methods (PoPS +), and (2) DEPICT intersects with any locus-specific methods (DEPICT +) (Fig. 1). Since the genome-wide gene prioritization approaches can prioritize different genes for different lipid types at the same locus, we report the gene prioritization results for all 2286 lipid-variant associations (Additional file 2: Table S2, Additional file 3: Figure S1).

We took the genes prioritized by PoPS + and performed text mining to determine whether previous biological evidence supported these genes as playing a role in lipid levels (Additional file 4: Table S3, S4). PoPS + leverages both locus-specific and genome-wide genetic signals to increase confidence level in prioritized genes [28]. In total, 882 out of 2286 lipid associations were assigned to one potential causal gene based on PoPS + . We identified a group of 466 unique genes among the 882 lipid associations. We determined that 31 out of the 466 PoPS + genes have over 1000 lipid-related publications, 91 PoPS + genes have 100–999 lipid-related publications, 321 PoPS + genes have 1–99 lipid-related publications, and 23 PoPS + genes had no lipid-related publications retrieved by the text mining algorithm. These 23 genes could indicate novel genes related to lipid levels for future work or be due to incorrect gene prioritization for a small fraction of index variants. (Additional file 5: Table S4). We then randomly selected 466 genes from 18,383 protein-coding genes using by the PoPS as the reference group to estimate the number of lipid-related publications we would expect to see by chance. A Mann–Whitney *U* test showed that there was a significant difference (*W* = 52,353, *p*-value < 2.2 x 10^−16^) between the set of genes identified by PoPS + compared to the reference set of 466 genes (Additional file 6: Figure S2). The median count of lipid-related publications was 19 for the PoPS + gene set compared with 2 lipid-related publications for genes in the reference set.

We performed a comprehensive lookup of all PoPS + prioritized lipid genes in the Therapeutic Target Database 2022 [24] and found 2092 drugs targeting at least one of our 102 PoPS + prioritized lipid genes observed in the database (Additional file 7: Table S5). Among those 102 PoPS + genes, we identify known drug target genes including *PCSK9* druggable as subtilisin/kexin type 9 inhibitor, *HMGCR* druggable as HMG-CoA reductase inhibitor, *PDE3A* druggable as phosphodiesterase 3A inhibitor (CILOSTAZOL), and *NR1H4 as a* bile acid receptor FXR agonist (URSODIOL). We also identify several other potential drug targets [24] such as *LIPG* (lipase G) and *NR1H3* (nuclear receptor subfamily 1 group H member 3), with relevant lipid biology. *LIPG* has phospholipase and triglyceride lipase activities and is a primary determinant of plasma HDL levels. *NR1H3* has an important role in the regulation of cholesterol homeostasis, regulating cholesterol uptake through MYLIP-dependent ubiquitination of LDLR, VLDLR, and LRP8 that could be targeted as an LXR-alpha modulator.

### Effects of protein-altering lipid alleles with protective effects on CAD, T2D, and NAFLD

Coronary artery disease (CAD), type 2 diabetes (T2D), and non-alcoholic fatty liver disease (NAFLD) are typically characterized by dyslipidemias. We examined protein-altering alleles with favorable lipid profiles for their associations with CAD, T2D, and NAFLD to identify potential cardiovascular drug targets without off-target liver or diabetes effects. Of the 2286 lipid associations, we observed 166 coding index variants. Eighteen coding variants with a protective lipid effect also had a protective effect for CAD or T2D (*p*-value < 0.001) and the lipid results colocalized with the CAD or T2D results, as appropriate, with a posterior probability of a shared causal variant > 0.8 (Table 1 and Additional file 8: Table S6). Six of these twenty variants had protective effects for both CAD and T2D, while nine were protective for CAD and three were protective for T2D (Table 1). Additionally, 269 noncoding alleles with a protective lipid effect also had a protective effect for CAD or T2D (*p* < 0.001; Additional file 8: Table S6).

**Table 1:** Protective lipid coding alleles associated with CAD and/or T2D

RSID	Trait	Coding variant	Effect allele (EAF)	Lipid effect	CAD OR (95% CI)	CAD *P*-value	T2D OR (95% CI)	T2D *P*-value	NAFLD OR (95% CI)	NAFLD *P*-value
**Protective lipid alleles associated with reduced risk of CAD and T2D**										
rs116843064	TG	*ANGPTL4* p.Glu40Lys	A (0.020)	− 0.238	0.87 (0.85,0.90)	1.92x10^−18^	0.91 (0.86,0.95)	2.30x10^−5^	0.99 (0.84,1.15)	0.85
rs1169288	TC	*HNF1A* p.Ile27Leu	A (0.682)	− 0.035	0.97 (0.96,0.98)	2.31x10^−16^	0.95 (0.94,0.96)	7.30x10^−13^	0.95 (0.91,1.00)	0.04
rs2307111	HDL-C	*POC5* p.His36Arg	C (0.440)	0.016	0.99 (0.98,0.99)	9.09x10^−05^	0.95 (0.94,0.96)	3.30x10^−16^	1.00 (0.96,1.05)	0.85
rs6480771	HDL-C	*DUSP13* p.Ser111Gly	T (0.531)	0.008	0.99 (0.98,0.99)	1.44x10^−04^	0.97 (0.96,0.99)	4.40x10^−05^	0.92 (0.88,0.97)	5.21x10^−04^
rs35742417	TG	*RREB1* p.Ser1554Tyr	A (0.173)	− 0.012	0.98 (0.97,0.99)	5.06x10 ^−04^	0.96 (0.95,0.98)	3.70 x10^−06^	0.98 (0.93,1.04)	0.60
rs72681869	TG	*SOS2* p.Pro191Arg	C (0.008)	− 0.053	0.93 (0.89,0.98)	3.71x10^−03^	0.88 (0.82,0.94)	3.90x10^−04^	0.87 (0.68,1.12)	0.29
**Protective lipid alleles associated with reduced risk of CAD**										
rs7412	LDL-C	*APOE* p.Arg176Cys	T (0.076)	− 0.517	0.90 (0.88,0.91)	9.94x10^−52^	1.01 (0.98,1.03)	0.55	1.01 (0.93,1.10)	0.84
rs11591147	LDL-C	*PCSK9* p.Arg46Leu	T (0.015)	− 0.434	0.80 (0.77,0.83)	5.97x10^−36^	1.04 (0.99,1.09)	0.16	1.05 (0.88,1.26)	0.58
rs11601507	LDL-C	*TRIM5* p.Val112Phe	C (0.926)	− 0.042	0.95 (0.94,0.96)	2.80x10^−12^	0.99 (0.96,1.01)	0.26	1.02 (0.93,1.11)	0.72
rs1132274	HDL-C	*RRBP1* p.Arg891Leu	C (0.827)	0.017	0.97 (0.96,0.98)	3.57x10^−08^	1.01 (0.99,1.02)	0.43	1.00 (0.94,1.07)	0.91
rs4760	HDL-C	*PLAUR* p.Leu317Pro	A (0.860)	0.016	0.97 (0.96,0.98)	7.31x10^−07^	0.99 (0.97,1.01)	0.3	0.96 (0.91,1.03)	0.26
rs855791	LDL-C	*TMPRSS6* p.Val736Ala	G (0.578)	− 0.009	0.98 (0.97,0.99)	1.08x10^−06^	1.00 (0.99,1.01)	0.75	0.94 (0.9,0.98)	4.83x10^−03^
rs58542926	TC	*TM6SF2* p.Glu167Lys	T (0.073)	− 0.124	0.97 (0.95,0.98)	4.02x10^−06^	1.10 (1.07,1.12)	2.00x10^−14^	1.45 (1.33,1.58)	1.05x10^−16^
rs56196860	HDL-C	*FKBP4* p.Asn197Lys	A (0.027)	0.031	0.95 (0.92,0.97)	1.05x10^-05^	0.98 (0.94,1.02)	0.33	1.03 (0.87,1.21)	0.73
rs72836561	HDL-C	*CD300LG* *p.Arg82Cys*	C (0.971)	0.187	0.95 (0.93,0.98)	1.34x10^−04^	0.98 (0.95,1.02)	0.4	0.98 (0.86,1.12)	0.77
**Protective lipid alleles associated with reduced risk of T2D**										
rs1800961	HDL-C	*HNF4A* p.Thr139Ile	C (0.969)	0.134	0.99 (0.97,1.01)	0.3751	0.85 (0.82,0.88)	3.20x10^− 20^	1.02 (0.90,1.17)	0.74
rs1801253	TG	*ADRB1* p.Gly389Arg	C (0.732)	− 0.011	1.01 (1.00,1.02)	8.76x10^− 03^	0.97 (0.96,0.98)	1.9010x^− 05^	1.00 (0.95,1.06)	0.85
rs13107325	HDL-C	*SLC39A8* p.Ala391Thr	C (0.941)	0.082	1.00 (0.98,1.01)	0.8876	0.95 (0.93,0.98)	3.30Ex10^− 04^	0.85 (0.78,0.93)	1.85x10^− 04^

### Driver tissues for lipid levels

We applied DESE (Driver-tissue Estimation by Selective Expression) [40] to estimate the driver tissues of five lipid traits using both gene-level and transcript-level eQTL summary statistics from GTEx v8 tissues [41]. We identified liver as the top-ranked tissue for HDL-C (gene-level *p*-value = 4.5 x 10^−18^, transcript-level *p*-value = 3.0 x 10^−26^), TC (gene-level *p*-value = 1.1 x 10^−25^, transcript-level *p*-value = 1.4 x 10^−33^), and nonHDL-C (gene-level *p*-value = 2.0 x 10^−19^, transcript-level *p*-value = 3.9 x 10^−29^) based on both gene-level and transcript-level selective expression (Additional file 9: Figure S3, Additional file 10: Table S7). For LDL-C, we identified the spleen as the top-ranked tissue using GTEx gene-level data (*p*-value = 8.3 x 10^−20^), while liver was ranked second (*p*-value = 4.8 x 10^−17^). However, when using GTEx transcript-level data, liver was the top-ranked tissue (*p*-value = 4.3 x 10^−29^) and second was whole blood (*p*-value = 4.3 x 10^−20^). The top tissue for TG according to both GTEx gene-level and transcript-level expression data was whole blood (gene-level *p*-value = 6.4 x 10^−20^, transcript-level *p*-value = 1.4 x 10^−21^). Spleen and liver were second according to GTEx gene-level and transcript-level expression data, respectively. The results were consistent with previous knowledge that the liver is a major tissue for lipid metabolism. Transcript-level selective expression provided more statistically significant results for the estimated driver tissues compared to the gene-level selective expression, as reported in the original [40].

### Polygenic scores for lipid phenotypes and phenome-wide association scans

We have previously reported that a polygenic score (PGS) for LDL-C was most informative when generated from the multi-ancestry GWAS and that the multi-ancestry PGS performed equally well in European-ancestry Americans, African-ancestry Americans, and continental Africans [24]. Using a similar approach, we generated PGS for the other four lipid traits (“Methods”).

We next performed a phenome-wide association scan (PheWAS) for the multi-ancestry lipid PGS (LDL-C PGS previously reported [24]) to identify pleiotropic effects of lipids with other traits in the European subsets of the UK Biobank and the Million Veteran Program (MVP) cohorts. We compared the effect sizes from the PheWAS analysis between the UK Biobank and MVP per lipid PGS and observed a moderate correlation between the two datasets (Additional file 11: Figure S4). The correlation of the PGS effects on all phenotypes between the UK Biobank and MVP ranges from 0.12 for the HDL-C PGS to 0.39 for the TC PGS (Additional file 11: Figure S4). In general, correlations were stronger for the ICD-10-based phecodes (*r*^2^ of 0.42–0.52) compared to the biomarkers (*r*^2^ of 0.06–0.23) (Additional file 11: Figure S4), which may reflect differences in study populations due to varied environmental effects, prevalence of chronic health conditions, and sex distribution. Among PheWAS results with *p*-value ≤ 0.05 in the UK Biobank, the correlation was even higher for ICD-10-based phecodes (*r*^2^ of 0.52–0.76) but remained the same for the biomarkers (*r*^2^ of 0.07–0.22).

**Fig. 2:**
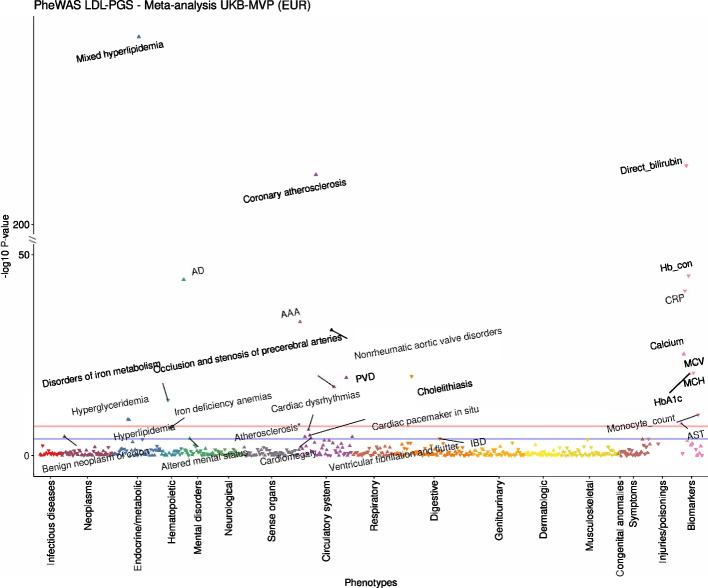
PheWAS meta-analysis results for multi-ancestry LDL-C PGS in the UK Biobank and MVP. The blue horizontal line denotes phenome-wide significance (*p*-value ≤ 6.5 × 10^−5^, to account for multiple testing of 773 phenotypes) and the red line is genome-wide significance (*p*-value ≤ 5 × 10^−8^). Phenotypes have been pruned, so that the most significant one per correlated phenotype group (correlation coefficient > 0.2) is retained. Pairwise correlations were estimated with chi-square test and Cramer’s V for the dichotomous phenotypes and Pearson’s correlation for the continuous phenotypes. AAA: abdominal aortic aneurysm, AD: Alzheimer’s disease, AST: aspartate aminotransferase, Atherosclerosis*: atherosclerosis of native arteries of the extremities with intermittent claudication, Hb_con: hemoglobin concentration, IBD: irritable bowel disease, MCH: mean corpuscular hemoglobin, MCV: mean corpuscular volume, PVD: peripheral vascular disease

We meta-analyzed the results from the two cohorts to increase the power of the PheWAS, by matching ICD10-mapped phecodes and biomarkers. In the combined the UK Biobank-MVP PheWAS results, we detected 58 phenotypes associated with the LDL-C PGS at phenome-wide significance level (*p*-value ≤ 6.5 × 10^−5^, corrected for 773 phenotypes), 165 with the HDL-C PGS, 59 with the TC PGS, 166 with the TG PGS, and 78 with the nonHDL-C PGS (Fig. 2, Additional file 12: Table S8, Additional file 13: Figure S5, Additional file 14: Figure S6, Additional file 15: Figure S7, Additional file 16: Figure S8). As expected, multiple cardiovascular phenotypes related to atherosclerosis, including the expected coronary artery disease as well as aortic aneurysm and essential hypertension, were phenome-wide significantly associated with all five lipid PGSs, indicating increased risk of these diseases for individuals with genetically predicted increased LDL-C, TG, TC, or nonHDL-C or genetically predicted decreased HDL-C. A recent wide-ranging Mendelian randomization analysis confirmed the causal effect of circulating lipids, not only for coronary artery disease, but other cardiovascular outcomes [42]. Additionally, all lipid PGSs were also significantly associated with decreased levels of direct bilirubin (Additional file 12: Table S8, Fig. 2, Additional file 13: Figure S5, Additional file 14: Figure S6, Additional file 15: Figure S7, Additional file 16: Figure S8), indicating genetically predicted lower LDL-C increased levels of bilirubin (Fig. 2). Correspondingly, lipid PGSs were associated with lower risk for cholelithiasis (gallstones) with the opposite direction for TG PGS, indicating that extreme lowering of LDL-C may impact rates of cholelithiasis (Additional file 12: Table S8, Fig. 2, Additional file 13: Figure S5, Additional file 14: Figure S6, Additional file 15: Figure S7, Additional file 16: Figure S8). To further clarify whether this association might be driven by the *ABCG8* gene alone, we excluded from the LDL-PGS all variants within the locus and tested the association between LDL-PGS and cholelithiasis in the UK Biobank. There was no attenuation of the observed association (OR = 0.94 and *p*-value = 7.94 × 10^−17^ without the ABCG8 locus vs. OR = 0.93 and *p*-value = 1.96 × 10^−21^).

In the PheWAS analysis, we found that the TC and LDL-C PGS were significantly associated with increased levels of HbA1c (beta = 0.101 mmol/mol per SD PGS increase, *p* -value= 1.21 × 10^−23^ and beta = 0.095 mmol/mol per SD PGS increase, *p*-value = 4.37 × 10^−21^, respectively), while the HDL-C PGS was associated with decreased levels of HbA1c (beta =  − 0.257 mmol/mol per SD PGS increase, *p*-value = 2.84 × 10^−143^) (Additional file 12: Table S8). Furthermore, genetically predicted increased LDL-C was significantly associated with decreased hemoglobin concentration (*p*-value = 1.92 × 10^−45^, similar significant associations for all other lipid PGSs with a reverse direction of effect for TG, Additional file 12: Table S8). As expected, genetically predicted increased LDL-C and TC were both associated with increased risk for Alzheimer’s disease [43] (OR = 1.33 per SD PGS increase, *p*-value = 1.74 × 10^−44^ and OR = 1.26 per SD PGS increase, *p*-value = 1.48 × 10^−30^, respectively; Additional file 12: Table S8). To further investigate how this association might be driven by the ApoE locus, we excluded all genetic variants overlapping this gene from the LDL-PGS and repeated the analysis in the UK Biobank. While the association between the LDL-PGS and the risk for Alzheimer’s disease was slightly attenuated after removing the ApoE locus (OR = 1.23 vs. 1.36 per SD PGS increase), the association remained significant (*p*-value = 2.51 × 10^−21^). Recent Mendelian randomization studies also provide evidence for the causal effect of lipids on risk for dementia [44] and Alzheimer’s disease [45]. The LDL-C and TC PGSs were also associated with increased aspartate aminotransferase levels (a liver enzyme), in accordance with other studies [46]. We also observed inverse associations between LDL-PGS (*p*-value = 1.43 × 10^−14^) and TC PGS (*p*-value = 8.34 × 10^−14^) with the risk of iron metabolism disorders (Additional file 12: Table S8).

To better understand the loci that drive the association between each of the lipid PGSs and cholelithiasis and cholecystitis, we interrogated the results from the single-variant PheWAS meta-analysis in the UK Biobank and MVP with all lipid multi-ancestry index variants (*N* = 1750 unique). We identified 22 genetic variants associated with cholelithiasis and/or cholecystitis at genome-wide significance. Genes prioritized for these index variants included genes already reported to be associated with gallstone disease [47] (*CYP7A1*, *ABCG5/8*), as well as additional genes (*ABCB4*, *LRBA*, *HNF4A*, *NUCB1*,* GATA4*), that may play also a role. Importantly, we found there was overlap (same index variant) between the previously published index variants for gallstone disease and our lipid index variants for these two loci (Additional file 17: Table S9).

### Lipid loci show sex-specific effects

Sex-stratified analyses have the potential to identify loci missed by sex-combined analyses [48] as well as to detect loci exhibiting differential effects on lipids between sexes. First, we performed GWAS meta-analysis separately in each sex (*N*_males_ = 749,391; *N*_females_ = 562,410), excluding loci discovered in the sex-combined analysis [24]. We identified twelve loci in females and four in males that reached genome-wide significance in the sex-stratified analysis (*p*-value < 5 × 10^−8^; Additional file 18: Table S10, Additional file 19: Table S11, Additional file 20: Table S12) but not in the sex-combined meta-analysis. As variants may show association to a single sex for reasons unrelated to biological sex differences, including differences in sample sizes between groups, we additionally tested for heterogeneity by sex for these variants in GLGC participating cohorts with close to equal number of males and females. Of the sixteen loci, eight showed significant sex-heterogeneity (*p*-value < 0.0031, Bonferroni-corrected threshold for sixteen tests). For example, the non-synonymous variant rs34372369 (*EPHA1*, p.Pro582Leu) is associated with nonHDL-C only in females (male *p*-value > 0.05) and shows significant sex-heterogeneity (*p*-value = 0.0012). This variant has been previously found to be linked with expression levels of the sex hormone-binding globulin gene (*SHBG*) more strongly in males than females [49], suggesting a possible reason for the difference in observed associations. We additionally sought to replicate the sex-heterogeneity results of these variants in 8 independent multi-ancestry cohorts (*N* = 311,639, 77% non-European ancestry, Additional file 21: Table S13). However, we did not detect significant differences in effect sizes between sexes for these variants after accounting for the number of tests (*p*-value > 0.0031, Additional file 22: Table S14), potentially due to the limited sample size or difference in ancestry makeup.

Second, we tested for a difference in the male- and female-specific effect sizes for each of the index variants identified from the sex-combined multi-ancestry meta-analysis. Of the 1750 unique index variants, 64 showed a significant difference in effect size by sex for one or more traits (Bonferroni correction for the number of index variants in each trait, Additional file 23: Table S15). These were evenly distributed across traits and more often had stronger effects in females than males (67%, Additional file 24: Figure S9). We tested for replication of the sex-specific differences in up to 311,120 participants from eight independent multi-ancestry cohorts not included in the original meta-analysis (Additional file 21: Table S13). Fifty-four of the 64 (84%) variants had effect size differences that were directionally consistent with the original meta-analysis (Additional file 25: Table S16). Of these, 10 had significantly different effect sizes (*p*-value < 7.8 × 10^−4^, Bonferroni correction for 64 variants) and 22 were nominally significant (*p*-value < 0.05). We attribute the low rate of replication to the small sample size and the differing proportions of ancestry groups within our replication samples, but we cannot dismiss the potential of false positives in the sex-specific discovery results.

We tested whether the observed sex differences could be caused by a higher frequency of cholesterol-lowering medications in males, potentially indicating an insufficient correction for pre-medication cholesterol levels. Among white British individuals in the UK Biobank, variants with significant sex differences had significantly higher effect size estimates on average after excluding individuals on medication (Additional file 26: Figure S10, Additional file 27: Table S17). However, of the 17 variants that exhibited a significant difference in effect size by sex in the UK Biobank alone, 15 remained significant after excluding individuals taking medications. Based on this observation, the observed differences did not appear to be driven solely, or even primarily, by differences in medication use between sexes. Furthermore, none of the identified sex-specific variants were associated with sex-participation bias [50] (Additional file 28: Table S18), indicating that differential study enrollment between sexes was unlikely to be the cause of the observed sex-specific lipid associations. We next investigated differences in environmental factors between sexes for these variants in the UK Biobank (Additional file 29: Table S19), including alcohol use [48], smoking status [48], body mass index (BMI) [51], and waist-hip ratio adjusted for BMI (WHRadjBMI) [51]. Twenty-two of the variants (34%) with differential effects on lipids by sex also exhibited a significant difference by sex for WHRadjBMI and one variant had a significant difference by sex for alcohol use (*ADH1B* p.His48Arg). The observed sex differences may therefore be partially attributed to pleiotropic associations with other traits.

**Fig. 3:**
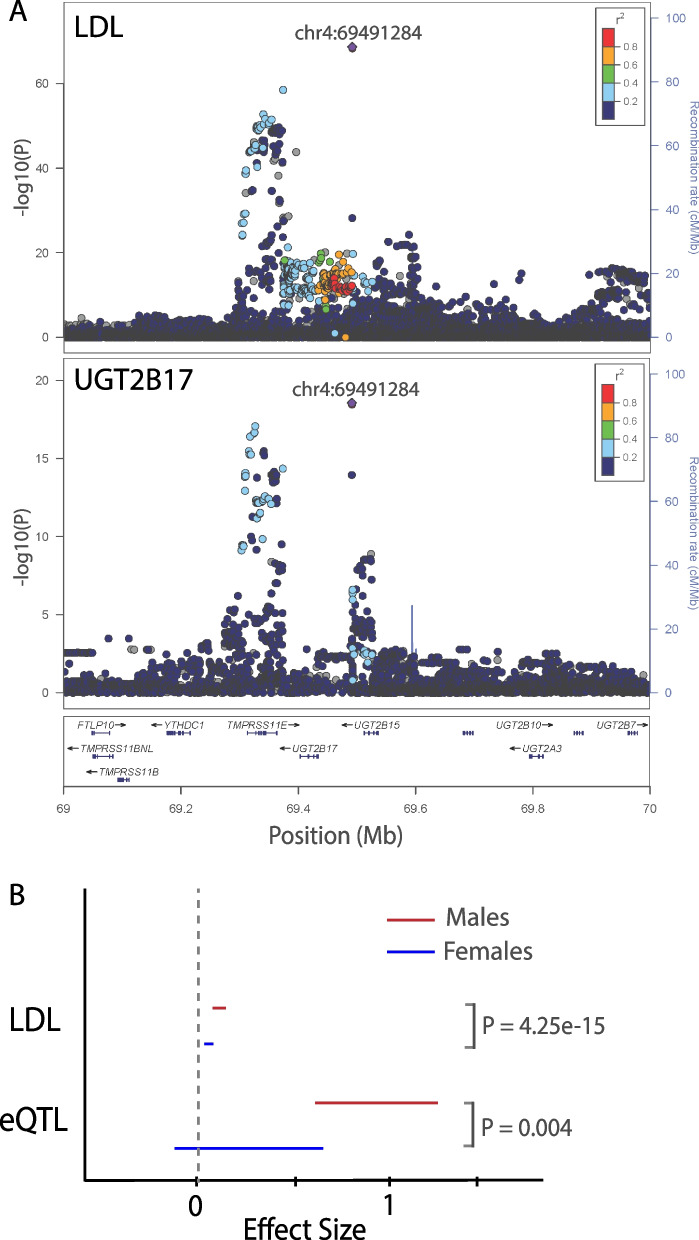
Sex specificity at the *UGT2B17* Locus. **A** The association signal for LDL-C (top panel) colocalizes with the *UGT2B17* eQTL signal in the liver (bottom panel). **B** The effect sizes of this variant on LDL-C and *UGT2B17* expression are both significantly higher for males (red) compared to females (blue)

Finally, we annotated each locus that showed significant sex differences with regulatory information to identify biological mechanisms that could underlie this difference. Of the 64 lipid variants with significant sex-stratified associations, 14 colocalized (posterior probability of H4 > 0.8) with expression of 20 genes in lipid-related tissues (liver, adipose, or skeletal muscle; Additional file 30: Table S20). Eight of these loci also show a sex-biased eQTL effect in at least one tissue in the direction concordant with the observed sex specificity of the GWAS effect (Additional file 30: Table S20). Among these ten is *CETP*, a gene with strong prior evidence for association with lipids, and *UGT2B17* [20] (Additional file 31: Supplementary Note, Fig. 3). The lead variant of *UGT2B17*, rs4860987, shows a significantly stronger effect of LDL-C in males (Beta_male_ = 0.042, SE_male_ = 0.002, Beta_female_ = 0.016, SE_female_ = 0.003, *p*-value_difference_ = 4.2 × 10^−15^) and colocalizes with a male-specific liver eQTL associated with increased expression of *UGT2B17*. Common variants at this locus are in moderate LD (*R*^2^ = 0.51) with a common copy number variation (CNV), which may mediate the causal effect (Additional file 31: Supplementary Note). *UGT2B17* plays a role in the metabolism of androgens [52], including testosterone, which is consistent with the observed pleiotropic relationship of this locus with testosterone in males (Additional file 30: Table S20). We note that the index variant in *UGT2B17*, rs4860987, did not show significant sex-specific effects in the replication cohorts, but this could be due to varying frequencies for the index variant between ancestry groups and the moderate LD to the causal CNV in the region. We observed that the combined frequency of rs4860987 across the replication studies was much lower (8%) compared with our combined frequency in the discovery (24%) due to differing proportions of ancestry groups and, along with the lower number of individuals (*N* = 218,437), led to a much-reduced power to replicate this sex-specific effect.

### Lipid-associated loci on the X chromosome

Lastly, we meta-analyzed association statistics for 3.1 million X chromosomal variants, including PAR regions, across 1,238,180 individuals from multiple ancestry groups. We identified 28 index variants significantly associated with lipid levels (Additional file 32: Table S21), of which 21 have not been previously reported [20, 39, 53] (15 for HDL-C, 4 for LDL-C, 4 for TC, 5 for TG and 4 for nonHDL-C, Table 2). Among these 28 loci, two have index variants with a minor allele frequency (MAF) < 1% and three index variants are missense mutations (in genes *ARSL*,* TSPAN6*, and *G6PD*), all of which are novel. We validated the identified X chromosomal associations in up to 255,475 individuals from seven multi-ancestry cohorts (Additional file 21: Table S13). Twenty index variants were at least nominally associated (*p*-value < 0.05), with five reaching genome-wide significance in the replication cohorts alone (*p*-value < 5 × 10^−8^, Additional file 32: Table S21).

**Table 2:** Novel X chromosome lipid-associated loci

RSID	Position in chromosome X (hg19)	EA/NEA	Annotation (closest gene)	Associated trait	EAF	*N*	Effect size (SE) from METAL	Ancestry and GC corrected *p*-value from MR-MEGA	Sex difference *p*-value
rs35143646	2,856,155	T/C	Missense (*ARSL*)	LDL-C	0.6352	1,038,070	0.0115 (0.0014)	1.83x10^− 16^	0.1791
				TC	0.6449	1,100,310	0.0091 (0.0014)	1.47x10^− 14^	0.08484
				nonHDL-C	0.6822	712,983	0.0112 (0.0017)	2.08x10^− 11^	0.5294
rs5934507	8,917,206	G/A	Intergenic (*FAM9B*)	HDL-C	0.274	1,158,000	− 0.0076 (0.0012)	9.99x10^− 10^	5.83x10^− 4^
rs191084933	14,133,208	G/A	Intergenic (*GEMIN8*)	HDL-C	0.0055	1,052,630	0.0694 (0.0101)	5.20x10^− 10^	0.3272
rs7888119	16,813,128	T/C	Intronic (*TXLNG*)	HDL-C	0.546	1,158,000	0.0114 (0.0012)	1.47x10^− 20^	0.2212
rs2230488	20,204,461	T/G	Synonymous (*RPS6KA3*)	LDL-C	0.1772	1,135,110	− 0.0143 (0.0015)	6.90x10^− 18^	0.9297
				TC	0.1745	1,237,380	− 0.0114 (0.0014)	4.68x10^− 12^	0.8906
rs6527977	20,322,238	A/C	Intergenic (*RPS6KA3*)	nonHDL-C	0.1602	789,200	− 0.0147 (0.0020)	3.06x10^− 10^	0.7000
rs12012576	21,813,178	G/A	Intergenic (*SMPX*)	TG	0.3103	1,160,340	− 0.0072 (0.0012)	4.88x10^− 8^	0.4558
rs6609434	46,636,767	C/A	Intergenic (*SLC9A7*)	HDL-C	0.4959	767,051	0.0060 (0.0014)	4.25x10^− 8^	0.9035
rs113957181	49,848,600	A/C	Intronic (*CLCN5*)	HDL-C	0.0566	452,268	− 0.0297 (0.0047)	1.39x10^− 9^	0.1896
rs782397956	53,993,589	T/C	Intronic (*PHF8*)	HDL-C	0.2050	621,342	− 0.0009 (0.0021)	2.65x10^− 15^	0.2585
rs72305711	55,981,911	T/TTA	Intronic (*RP13-188A5.1*)	HDL-C	0.1111	534,967	− 0.0167 (0.0033)	2.22x10^− 22^	0.03202
rs5914559	56,139,739	G/T	Intergenic (*KLF8*)	TG	0.6697	738,155	− 0.0131 (0.0016)	4.44x10^− 16^	0.3224
rs5964416	64,368,487	A/C	Intergenic (*ZC4H2*)	HDL-C	0.0924	614,283	− 0.0316 (0.0016)	2.68x10^− 11^	0.1478
rs5965342	66,204,144	T/C	Intergenic (*EDA2R*)	HDL-C	0.2037	745,721	− 0.0212 (0.0020)	1.20x10^− 29^	0.02050
rs505520	66,258,914	C/A	Intergenic (*EDA2R*)	TG	0.2191	750,415	0.0217 (0.0019)	2.91x10^− 30^	0.004371
rs771540123	67,967,645	A/G	Intergenic (*STARD8*)	nonHDL-C	0.0027	15,311	− 0.0202 (0.115)	1.61x10^− 9^	NA
rs5937000	70,047,788	C/T	Intronic (*TEX11*)	HDL-C	0.4515	796,971	0.0075 (0.0013)	9.55x10^− 9^	0.02603
rs5938008	74,496,225	C/T	Intronic (*UPRT*)	HDL-C	0.9167	1,072,550	0.0174 (0.003)	1.24x10^− 8^	0.7235
rs1802288	99,890,204	T/C	Missense (*TSPAN6*)	LDL-C	0.1665	714,113	0.0159 (0.0019)	1.42x10^− 16^	0.9642
				TC	0.1684	753,479	0.0128 (0.0018)	2.49x10^− 10^	0.8646
rs139144471	117,829,694	G/T	Intergenic (*DOCK11*)	HDL-C	0.0876	1,098,540	− 0.0121 (0.0022)	1.16x10^− 8^	0.1111
rs6648533	122,804,678	C/T	Intronic (*THOC2*)	HDL-C	0.299	1,106,090	− 0.0100 (0.0013)	2.24x10^− 12^	0.4516
rs5929738	135,265,287	C/A	Intronic (*FHL1*)	HDL-C	0.4679	970,330	0.0071 (0.0011)	3.21x10^− 10^	0.4197
rs5975692	135,266,089	G/A	Intronic (*FHL1*)	TG	0.465	1,138,740	− 0.0084 (0.0011)	7.87x10^− 12^	0.9215
rs2070826	153,582,198	C/T	Intronic (*FLNA*)	HDL-C	0.1427	1,141,020	0.0170 (0.0017)	6.65x10^− 26^	0.08671
rs11593	153,627,145	C/A	Intronic (*RPL10*)	TG	0.1586	1,143,360	− 0.0166 (0.0017)	5.76x10^− 21^	0.8695
rs7886627	153,679,609	G/A	Intergenic (*FAM50A*)	nonHDL-C	0.1214	771,706	− 0.0147 (0.0022)	9.26x10^− 9^	0.06819
rs1050828	153,764,217	T/C	Missense (*G6PD*)	LDL-C	0.0113	744,968	− 0.0514 (0.0061)	2.54x10^− 15^	0.6533
rs762517	153,764,734	A/G	Intronic (*G6PD*)	TC	0.0142	798,600	− 0.0480 (0.0057)	5.26x10^− 16^	0.7594

*EA* Effect allele, *NEA* Non-effect allele, *EAF* Effect allele frequency, *N* Number of samples, *SE* Standard error of the effect size, *GC* Genomic control, *LDL-C* Low-density lipoprotein cholesterol, *TC* Total cholesterol, *HDL-C* High-density lipoprotein cholesterol, *TG* Triglycerides

We additionally considered potential sex differences for the X chromosome variants. A missense variant in *RENBP* with MAF = 2.5% reached genome-wide significance only in males but was not significant in the sex-combined meta-analysis or in the female-only analysis (*p*-value = 4.59 × 10^−8^, 0.003 and 0.2, respectively). We also observe three X chromosome loci with significant heterogeneity in effects between sexes; however, these were not significant in the replication cohorts alone, possibly due to the lower sample size (Bonferroni correction for the number of index variants in each trait, Additional file 32: Table S21).

Using a PheWAS approach in the UK Biobank, we found four of the novel loci to have pleiotropic associations with body composition traits (*FAM9B* [HDL-C], *EDA2R* [HDL, TG], *TSPAN6* [LDL-C, TC], and *DOCK11* [HDL-C]), four variants with coronary atherosclerosis and ischemic heart disease, three with immune-related biomarkers (*SLC9A7* [HDL-C], *CLCN5* [HDL-C], *THOC2* [HDL-C]), and two with blood clotting-related biomarkers (*KLF8* [TG], *TEX11* [HDL-C]) (Additional file 32: Table S21). Interestingly, two of the three sex-biased X chromosome variants demonstrate the most significant association with testosterone of all lipid X chromosome variants tested in the PheWAS (rs505520: beta/SE =  − 0.089/0.007 nmol/L per TG-increasing allele and rs5934507: beta/SE = 0.237/0.006 nmol/L per HDL-increasing allele).

## Discussion

In this study, we identify and prioritize likely candidate genes at lipid-associated loci discovered through a variety of approaches including multi-ancestry meta-analysis of autosomes [24] (~ 91 million variants) and the X chromosome (~ 3 million variants), as well as sex-specific meta-analyses using sample sizes ranging from 1.35 to 1.65 million individuals. We previously reported a comparison of multi-ancestry vs single-ancestry lipid findings using autosomal chromosomes and identified improvements in fine-mapping of credible sets and PGS performance, with slight differences in the number of identified loci by ancestry group [24]. Here, we add X chromosome and sex-specific discovery results. We also focus on lipid biology by prioritizing implicated genes, identifying novel phenotypes and diseases associated with genetically predicted lipid levels, and predicting candidate drug target genes.

Our results from this effort translate our GWAS findings for three complimentary research areas, helping us further elucidate the biological mechanisms underlying the lipid-associated genetic variants. We first sought to identify methods for prioritization of functional genes at GWAS loci by performing six gene prioritization methods. Lipids are an excellent exemplar phenotype for gene prioritization algorithms because of a wealth of GWAS loci (~ 1000), Mendelian dyslipidemia genes (21), and mouse dyslipidemia phenotypes observed in gene knockouts (740). While the gene prioritization approaches are not independent of each other, integrating several prioritization predictors provides higher confidence when attempting to characterize causal genes. Others have also highlighted the importance of such frameworks in different diseases [29, 54, 55].

We identify 466 unique genes by combining evidence from a global approach (PoPS) with local gene prioritization approaches. The vast majority of these genes had many lipid-related publications, suggesting the accuracy of our combined prioritization approach. Twenty-three PoPS + identified genes had no lipid-related publications, indicating they could be truly novel or possibly were incorrectly prioritized. Functional validation of the larger pool of prioritized genes, which will require highly parallel experimental methods, will help to further optimize bioinformatics algorithms to prioritize genes and is beyond the scope of this manuscript.

Our prioritization approach also indicates several genes as potential drug targets including *PDE3A* and *NR1H4*. *PDE3A* encodes the phosphodiesterase 3A gene and is predicted to be druggable as phosphodiesterase 3A inhibitor (CILOSTAZOL). Cilostazol has antiplatelet, anti-proliferative, vasodilatory, and ischemic-reperfusion protective properties [56] and has been previously suggested for the primary or secondary prevention of CAD [22]. *NR1H4* encodes a bile acid receptor and regulates the expression of genes involved in bile acid synthesis and transport. The target gene is predicted to be druggable as *a* bile acid receptor FXR agonist (URSODIOL). Ursodiol is used to treat primary biliary cirrhosis and cholelithiasis and could be a potential candidate for drug repurposing.

We also identify eighteen coding variants where the protective lipid allele is also protective for CAD or T2D. Among these, *PCSK9* is a well-documented drug target, not only for lipids but also for cardiovascular events [57–59]. In comparison to published studies [60], others find a non-significant increased risk for T2D [61] and an arguably stronger protective effect for CAD [62], for *PCSK9* variant carriers. Our observation is consistent with the lack of excess T2D risk observed in PCSK9 inhibitor clinical trials [57–59, 63] and with strong protective effects for coronary heart disease [64]. Furthermore, these variants are potential therapeutic targets for protective lipid profiles and lowering risk of disease.

Our second goal was to identify diseases that may benefit from lipid-lowering as well as diseases or traits that may become problematic due to very low lipids. To accomplish this, we examined the association of genetically predicted lipid traits (using PGS) with 773 phenotypes in 478,556 individuals. We observed that genetically predicted increased LDL-C, TC, and HDL-C levels, or decreased TG levels, decrease the risk of cholelithiasis. Prior epidemiological studies have not consistently reported an association between lipid levels and risk of gallstones, with some studies showing that increased levels of LDL-C, TC, and TG and decreased levels of HDL-C predispose to the risk for cholelithiasis [65, 66], but others showing no association [67, 68]. Our results are corroborated by a recent Mendelian randomization meta-analysis study in the FinGen and UK Biobank cohorts [69]. The prioritized genes for the individual index lipid variants significantly associated with cholelithiasis in the PheWAS analysis include *ABCG8*, a hepatic cholesterol transporter, responsible for the efflux of cholesterol from the enterocytes to the lumen and from the hepatocytes into bile [70]. The lipid-decreasing allele of index variant in *ABCG8*, rs4245791, has been previously associated with high risk for gallstone disease [47] and high intestinal cholesterol absorption [71], possibly mediated by an increased expression of *ABCG8* [72]. Furthermore, even after excluding *ABCG5/8* variants from the LDL-PGS, the association with the risk of cholelithiasis was not attenuated. These PGS-PheWAS results suggest the existence of many other cholesterol transporters like *ABCG8* that modify blood cholesterol levels perhaps in large part by facilitating an increased secretion of cholesterol into the biliary system, which in turn increases the risk of the formation of gallstones through the supersaturation of bile. We also observed that HbA1c levels were elevated among subjects with genetically predicted increased LDL-C and TC and with genetically predicted decreased HDL-C. Previous epidemiological studies have established associations between dyslipidemia (increased LDL-C, TC, TG, and decreased HDL-C levels) and increased HbA1c levels among subjects with T2D, as well as insulin-resistant subjects without diabetes [73, 74]. Our observations support a strong genetic basis to these associations and are in accordance with previous studies showing shared pathways between lipid biology, T2D, and HbA1c [75], as well as pleiotropic effects of blood red cell variants with lipid levels [76]. Mendelian randomization studies have shown that hemoglobin and LDL show bidirectional inverse relationships and hemoglobin effects on LDL are also mediated through Hb1Ac, implying that genetic variation influencing erythrocytic factors could also determine lipid levels and the opposite [77]. While most of our significant PheWAS findings could be confirmed via Mendelian randomization studies, we cannot exclude the possibility of spurious associations due to pleiotropy.

Lastly, we sought to expand the coverage of the genome and performed the most comprehensive GWAS of lipid levels to date by including assessment of 3 million variants on the X chromosome as well as explicitly testing for sex-specific effects across 23 chromosomes in 1.35 million individuals of diverse ancestries. We report 21 novel X chromosome loci, including an LDL-lowering locus involving a missense variant in *G6PD*, known to cause G6PD deficiency (p.V68M) [78]. The proposed mechanism is via the inhibition of the NADPH-dependent hydroxymethylglutaryl-CoA (HMG-CoA) reductase, resulting in decreased cholesterol biosynthesis, even though the protective effect of the G6PD deficiency on cardiovascular risk is debatable [79].

We also observed that approximately 3–5% of the genome-wide lipid index variants exhibited differential effects between sexes, which did not seem to be due to differential prevalence in the use of lipid medications or study selection bias. These findings may have important implications in the interpretation of lipid biology, the identification of novel drug targets, and possibly for more accurate prediction of blood cholesterol-related conditions. For example, the *UGT2B17* locus, one of the ten sex-biased loci with corresponding sex-biased eQTL effect, is known to be implicated in androgen and drug metabolism [52]. A common CNV in the region, partially tagged by the lipid index variant, is associated with significant variations in expression levels between ethnic groups [80], which would explain lack of replication in the set of independent studies, and the deletion has been linked to testosterone-related decreased BMI levels [81], as well as decreased risk for osteoporosis in men [82].

Several of the reported sex-biased and X chromosome loci showed significant pleiotropic effects with sex hormone levels, including testosterone and SHBG, highlighting the role of hormone regulation in lipid metabolism [83]. In particular, we observe an overall inverse effect between the X chromosome lipid index variants and the sex hormone levels. Observational studies have long suggested a potential influence of the sex hormones on the risk for cardiovascular risk [84] but this hypothesis has not been consistently supported by recent Mendelian randomization studies, raising the issues of reverse causality [85, 86].

## Conclusions

In conclusion, we leverage the power of a large multi-ancestry GWAS study to further our understanding of lipid metabolism and the impact on chronic diseases. We identify novel lipid loci on the X chromosome and autosomal loci with evident sex-biased lipid effects. We compare a range of gene prioritizing methods to identify causal genes, an approach applicable to studying other complex traits. We additionally further our understanding of lipid metabolism through a phenome-wide study that implicates a relationship between genetically predicted low cholesterol with risk of cholelithiasis.

## Methods

### Meta-analysis

Summary statistics for sex-combined autosomal analyses were previously published [24]. Following the same procedure, we carried out meta-analyses stratified by sex for 5 lipid traits (HDL-C, LDL-C, TG, nonHDL-C, and TC) for both the autosomes and chromosome X. The sample size for chromosome X (Total *N* = 1,238,180; males = 749,391; females = 562,410) was lower than available for autosomes as not all participating biobanks submitted results for chromosome X. Quality control of summary statistics from contributing cohorts was performed using EasyQC [87]. Prior to meta-analysis, we removed variants with low imputation info scores (*r*^2^ < 0.3), those with minor allele count < 3, and those with Hardy–Weinberg equilibrium *p*-value < 1 × 10^−8^. Variants on the X chromosome were filtered using the female imputation info scores and Hardy–Weinberg equilibrium *p*-values. Summary statistics were corrected by the genomic-control factor calculated from the median *p*-value of variants with minor allele frequency > 0.5%. For cohorts that contributed summary statistics imputed both on the Haplotype Reference Consortium (HRC) and 1000 Genomes Population v3 (1KGP3) panels, we generated a single file containing all possible variants, favoring those imputed from the HRC imputation panel due to generally higher imputation quality of these variants. Multi-ancestry meta-analysis was performed with MR-MEGA [88] with 5 principal components and using the inverse-variance weighted method in METAL to estimate effect sizes [89]. Independent loci were defined with physical distance > 500 kb or genetic distance > 0.25 cM, whichever one would result in a larger window, followed by a conditional analysis using rareGWAMA [90] as previously described [24], to identify index variants that were shadows of nearby, more-significant associations. Conditional analysis for chromosome X used a female-only UK Biobank LD reference (*N* = 21,510). In line with the analysis in the autosomes, a locus was identified as dependent if the effect size after conditioning on the most significant variant in the area was more than 1.43 times smaller than the original (95th percentile of the effect size ratios for chromosome X).

Differences in effect size between males and females were tested within each cohort using [91]:

$$Z=\frac{{B}_{m}-{B}_{f}}{\sqrt{{se}_{m}^{2}+{se}_{f}^{2}-2*r*{se}_{f}*{se}_{m}}}$$

and were then meta-analyzed across studies using METAL, to account for cohort-specific ascertainment (e.g., enrichment of cases for type 2 diabetes), or demographics, such as age.

### Replication

We collected summary statistics from 8 cohorts across 6 ancestry groups, including African or African American, East Asian, European, Hispanic, Middle Eastern, and South Asian. Each cohort provided sex-stratified and X chromosome association results for the tested traits, as available. The difference in effect sizes between males and females was calculated within each cohort as described above and then meta-analyzed across studies using METAL. X chromosome association results were meta-analyzed using METAL with weighting by sample size.

### Gene prioritization methods

#### Closest gene

We annotated the closest gene to the lipid multi-ancestry index variants [24] by identifying the closest gene transcript on either side (500 kb) of the index variant [92].

#### Colocalization with GTEx eQTLs

For each of the five lipid phenotypes, we first lifted over GWAS summary statistics from the multi-ancestry meta-analysis [24] to GRCh38 using the UCSC liftOver tool. Then, we defined a set of approximately independent windows across the genome within which colocalization with eQTLs was run. To define these, we first identified all genome-wide significant variants (*p*-value < 5e − 08) from the meta-analysis for each lipid trait and sorted them by significance, from most significant to least. Starting with the most significant variant, we aimed to define a window defining independent genetic signals; we define a variant’s window as a region within the greater of 500 kb or 0.25 cM on either side of this “sentinel variant.” Genetic distances were defined using reference maps from HapMap 3. All other genome-wide significant variants within this window were discarded from the list of sentinel variants, and similar windows were defined for the remaining genome-wide significant variants.

We ran an eQTL colocalization using GTEx v8 eQTL summary statistics within each of our defined windows for all lipid traits. For each of the 49 GTEx tissues, we first identified all genes within 1 Mb of the sentinel variant, and then restricted analysis to those genes with eQTLs (“eGenes”) in that tissue (FDR < 0.05). We used the R package “coloc” (run on R version 3.4.3, coloc version 3.2.1) [93] with default parameters to run colocalization between the GWAS signal and the eQTL signal for each of these cis-eGenes, using as input those variants in the defined window, i.e., all variants present in both datasets. A colocalization posterior probability of (PP3 + PP4) > 0.8 was used to identify loci with enough colocalization power, and PP4/PP3 > 0.9 was used to define those loci that show significant colocalization [94].

#### Transcriptome-wide association studies (TWAS)

For our transcriptome-wide association analysis (TWAS), we integrated the results of our GWAS with eQTL summary statistics from GTEx v8. The S-PrediXcan software [95] allows us to integrate these two datasets using only summary statistics from GWAS without needing individual-level genotype data. We used the multi-ancestry lipid GWAS summary statistics [24] and harmonized them with the GTEx summary statistics. Then we performed the TWAS using the eQTL models estimated on GTEx v8 expression data. For each of the 49 GTEx tissues, we identified “significant genes” those genes with *p*-values more significant than an FDR threshold of 0.05.

#### Genes with coding variants

We determine the coding variants within 99% credible sets and the coding variants in LD > 0.8 with variants in the 99% credible sets with the credible sets as defined here [24]. We define regions for construction of the credible sets as ± 500 kb around each index variant. We used Bayes factors (BFs) for each variant from the MR-MEGA output and generated the credible sets within each region by ranking all variants by BF and calculating the number of variants required to reach a cumulative probability of at least 99%. Additionally, we used previously established gene-based associations [96] to determine whether rare coding variation in a gene were associated with blood lipid levels (*p* < 0.001). We labeled a gene as having coding variants if any of these criteria were met.

#### DEPICT

We used Data-driven Expression-Prioritized Integration for Complex Traits (DEPICT, v1 beta version rel194 for 1 KG imputed GWAS) to prioritize genes at our index variants, on the assumption that truly associated genes share functional annotations [27]. Index variants [24] with *p*-value < 5 x 10^−8^ were retained as input. We implemented the DEPICT analysis with the default settings of 500 permutations for bias adjustment and 20 replications for FDR estimation. DEPICT prioritizes genes by calculating the similarity of a given gene to genes from other associated loci across 14,461 reconstituted gene sets and estimates the nominal gene prioritization *p*-value and the estimated false discovery rate of each gene. The prioritized genes at FDR < 0.05 were considered significant.

#### PoPS

We used the PoPS method to prioritize genes in the previously reported [24] multi-ancestry index variants for all lipid traits. The PoPS method [28] is a new gene prioritization method that identifies the causal genes by integrating GWAS summary statistics with gene expression, biological pathway, and predicted protein–protein interaction data. First, as part of the PoPS analysis, we used MAGMA to compute gene association statistics (*z*-scores) and gene–gene correlations from GWAS summary statistics and LD information from a multi-ancestry reference panel (1000 Genomes). Next, PoPS performs marginal feature selection by using MAGMA to perform enrichment analysis for each gene feature separately. The model is fitted by generalized least squares (GLS), and MAGMA results are used to perform marginal feature selection, retaining only features that pass a nominal significance threshold (*p* < 0.05). Then PoPS computes a joint enrichment of all selected features simultaneously in a leave one chromosome out (LOCO) framework. The gene features employed by PoPS are listed here: https://github.com/FinucaneLab/gene_features. Finally, PoPS computes polygenic priority scores for each gene by fitting a joint model for the enrichment of all selected features. The PoPS score for a gene is independent of the GWAS data on the chromosome where the gene is located. The PoPS analysis returned scores for a total of 18,383 genes per lipid trait. We only kept the top 20% genes among all 18,383 genes. We then annotated our index variants with the nearest ENSEMBL genes in a 500-kb window (either side) and selected the highest PoPS score gene in the locus as the prioritized one.

We performed the PoPS analysis on our lipid-specific multi-ancestry meta-analysis results, using all populations from 1000G as the reference for the LD information in MAGMA. As a sensitivity step, we also repeated the same analysis using only the European population from 1000G as the reference. We observed high concordance in the top two PoPS prioritized genes from both reference panels. In detail, the same 2119 genes (89%) were prioritized as the top genes from both panels, a further 203 genes were prioritized as a top gene with one panel and as the second top with the other and only 7 genes were completely mismatched between the two reference panels.

#### Monogenic genes

We annotated genes known to cause Mendelian lipid disorders based on proximity with identified GWAS loci [97, 98]. GWAS index variants within ± 500 kb of the transcription start and end positions from the USCS genome browser annotations were annotated as nearby known monogenic dyslipidemia genes.

#### Mouse knockout lipid phenotype silver set genes

Human gene symbols (9557 unique genes) were mapped to gene identifiers (HGNC) and their corresponding mouse ortholog genes were obtained using Ensembl (www.ensembl.org). Phenotype data for single-gene knockout mouse models were obtained from the International Mouse Phenotyping Consortium (IMPC) (www.mousephenotype.org) latest data release 12.0 (www.mousephenotype.org/data/release). The knockout mouse models were primarily produced by IMPC but also include some models that have been reported from the relevant literature and were curated by Mouse Genome Informatics (MGI) data release 6.16 (www.informatics.jax.org). For each mouse model, reported phenotypes were grouped using the mammalian phenotype ontology hierarchy into broad categories relevant to lipids: growth and body weight (MP:0001259), lipid homeostasis (MP:0002118), cholesterol homeostasis (MP:0005278), and lipid metabolism (MP:0013245). This resulted in mapping of human genes to significant phenotypes in animals.

For each of the multi-ancestry lipid index variant [24], we mapped the closest gene to the knockout mouse phenotypes and curated the set to only include mouse phenotypes strictly relating to lipid metabolism. That resulted in our silver set of 740 genes with mouse lipid phenotypes (Additional file 33: Table S22).

#### Overlap between methods

We standardized the gene names across different methods using the R/geneSynonym package, a wrapper to gene synonym information in ftp://ftp.ncbi.nlm.nih.gov/gene/DATA/gene_info.gz. We also quantified how often the same gene was prioritized by multiple methods for each index variant and determined scores that ranged from 1 to 6 (S1-S6), based on the number of methods that prioritized the gene.

We integrated multiple gene prioritization methods to identify likely causal genes in the latest global lipid genetics consortium GWAS results. In total, we have implemented the 6 individual gene prioritization methods above that utilize the GWAS summary statistics from meta-analysis. Our gene prioritization methods can be placed into two broad categories, the locus-specific methods and the genome-wide methods. The locus-specific methods leverage local GWAS data by connecting the causal variants to the causal gene(s) using genomic distance, eQTLs, or protein-coding variants.

More specifically, there are four locus-specific methods that have been implemented including: (1) The closest protein-coding gene around the index variants based on the genomic distance, (2) eQTL colocalization using r COLOC package, (3) TWAS using S-PrediXcan, (4) coding variants which have been identified in 99% credible sets OR in LD > 0.8 with coding variants OR from gene-based tests (*p* < 0.001) of rare coding variants. For the eQTL and TWAS, we first used all the 49 GTEx tissues and then restricted to only 5 lipid-specific tissues: liver, adipose subcutaneous, adipose visceral, whole blood, and small intestine. In addition, two genome-wide methods were employed: (1) DEPICT (FDR < 0.05), (2) PoPS (Top 1 gene). It is reasonable to combine similarity-based methods with locus-based methods since they use two different sources of information.

To determine the relative performance of each prioritization method and their combined scores for lipid loci, we used 21 genes known to cause Mendelian dyslipidemias as a gold standard set (*ABCA1*,* ABCG5*,* ANGPTL3*,* APOA5*,* APOB*,* APOE*,* CETP*,* CYP27A1*,* GPD1*,* GPIHBP1*,* LCAT*,* LDLR*,* LDLRAP1*,* LIPA*,* LIPC*,* LMF1*,* LPL*,* MTTP*,* PCSK9*,* SAR1B*,* SCARB1*), and 740 mouse knockout genes causing lipid phenotypes as a silver standard set (Additional file 33: Table S22). We examined two metrics for each gene prioritization approach: (1) the proportion of prioritized genes in the gold/silver standard set, and (2) the proportion of correctly identified genes among all prioritized genes (Additional file 3: Figure S1). Note that out of the 2286 lipid associations, 97 fell within 500 kb of a Mendelian gene and 1280 within 500 kb of a mouse knockout gene with a lipid phenotype. We observed that the TWAS results yielded a high number of prioritized genes, but lead to a low proportion correctly identified. The TWAS approach had a much smaller proportion of genes correctly prioritized among all the prioritized genes, given it prioritized a total of 3511 genes, which was 3.5-fold greater than the other methods (~ 1000 genes). Notably, PoPS provided a similar proportion of correctly identified genes (78%) as of TWAS, while retained a high proportion of prioritized genes in the gold standard set (67%). Compared with PoPS, PoPS + (PoPS plus one of the local methods) slightly sacrificed the proportion of correctly identified genes from 78 to 71%, but improved the proportion of prioritized genes in the gold standard set from 67% to 79%. Overall, PoPS/PoPS + outperform other gene prioritization methods on both metrics for our gold (Additional file 3: Figure S1A) and silver (Additional file 3: Figure S1B) standard gene sets. We also assessed lipid-relevant tissue (liver, subcutaneous and visceral adipose, whole blood, and small intestine) expression QTLs (lipid eQTLs) and transcriptome-wide association (lipid TWAS) and found that the expression results from all tissues performed slightly better at recovering the reference gene sets compared with limiting to the lipid-relevant tissues (Additional file 3: Figure S1).

#### Text mining analysis

We retrieved the whole MEDLINE/PubMed titles and abstracts as of March 06, 2022, from National Library of Medicine (https://ftp.ncbi.nlm.nih.gov/pubmed/baseline/; https://ftp.ncbi.nlm.nih.gov/pubmed/updatefiles/). We then examined whether a list of genes prioritized by PoPS + and any one of the lipid-related keywords (lipid, lipids, triglyceride, triglycerides, fatty acid, cholesterol, dyslipidemias, hyperlipidemia, hypercholesteremia, diabetes, type 2 diabetes, type II diabetes, heart, cardiovascular, artery, coronary, coronary artery, coronary heart, atherosclerosis, peripheral vascular, PAD, stroke) occurred in the same abstract. We counted how many lipid-related publications that have a specific gene co-occurred with at least one lipid-related keyword. The same text mining approach was also implemented to a set of randomly selected genes from the 18,383 protein-coding genes used by the PoPS. We estimated the number of lipid-related publications we would expect to see by chance. A Mann–Whitney *U* test was performed to show whether there was a significant difference between the number of lipid-related publications of the PoPS + gene set and reference gene set.

#### Drug target mining analysis

To gain therapeutic insights from our gene prioritization results, we performed a lookup in Therapeutic Target Database (TTD) 2022 [99] (http://db.idrblab.net/ttd/). Specifically, we cross-referenced 466 unique lipid-associated genes prioritized by PoPS + (Additional file 2: Table S2) with 1563 genes corresponding to at least one drug (either under development or approved) with known clinical indication in TTD 2022. As a quality control for this lookup, we excluded all TTD entries related to drugs that were discontinued, terminated, or withdrawn from the market. The full lookup results are available in Additional file 8: Table S6.

#### Driver tissues for lipid levels

We performed phenotype-tissue association analysis using DESE (driver-tissue estimation by selective expression) [40]. DESE estimates the causal tissues by selective expression of phenotype-associated genes in GWAS. We used the GWAS summary statistics from the five lipid traits and the GTEx v8 normalized gene-level and transcript-level expression datasets as input. SNPs inside a gene and its ± 5 kb adjacent regions were first mapped to the gene, and then DESE ran iteratively to produce a list of driver tissues and the corresponding *p*-values of the associations. We used a Bonferroni-corrected significance threshold of 0.05/54 = 9.3 x 10^−4^.

### PheWAS analysis

#### Construction of lipid PGSs

We had previously developed a multi-ancestry PGS for LDL-C that was demonstrated to perform well across multiple ancestry groups [24]. In a similar manner, we also generated PGS for HDL-C, nonHDL-C, TC, and triglycerides. First, multi-ancestry meta-analysis results were generated with METAL [89] after excluding individuals from the Michigan Genomics Initiative and the UK Biobank. The set of variants used to construct the PGS was limited to those that were well-imputed (*R*^2^ > 0.3) in MGI, UK Biobank, and MVP. Risk scores based on PRS-CS [100] or pruning and thresholding with Plink [101] across several *r*^2^ (0.1, 0.2), distance (250 kb, 500 kb), and *p*-value thresholds (5 × 10^−10^, 5 × 10^−9^, 5 × 10^−8^, 5 × 10^−7^, 5 × 10^−6^, 5 × 10^−5^, 5 × 10^−4^, 5 × 10^−3^, 0.05) were developed. For each trait, the single best score was selected based on the adjusted *r*^2^ calculated in the UK Biobank of the linear model for the lipid trait with the risk score and age, sex, batch, and PC1-4 as covariates. This corresponded to PRS-CS for HDL-C and nonHDL-C and pruning and thresholding for LDL-C (*r*^2^ = 0.1, *p*-value = 5 × 10^−4^, 500 kb), TG (*r*^2^ = 0.1, *p*-value = 5 × 10^−3^, 500 kb), and TC (*r*^2^ = 0.1, *p*-value = 5 × 10^−4^, 500 kb). The variance explained by the risk score among the UK Biobank participants was similar across traits (adjusted *r*^2^ of the full model-adjusted *r*^2^ of covariates: HDL-C = 0.13; LDL-C = 0.15; nonHDL-C = 0.14; TC = 0.14; TG = 0.10) and validated the ability of the risk score to predict genetically increased lipid levels.

#### PheWAS of lipid PGSs and index lipid variants in the UK Biobank and MVP

We used the European ancestry subset of individuals from the UK Biobank (408,886 samples) and the European samples from MVP (69,670 samples) to perform the PheWAS analysis.

We constructed a weighted PGS for each of the lipid traits, based on the corresponding genome-wide significant multi-ancestry index variants. We used the PheWAS package in R [102] to map ICD-10 codes from hospital records into clinically relevant phenotypes (phecodes) and to implement these association analyses, while adjusting for sex, age, 10 genetic principal components, and genotyping array (for the UK Biobank only) in each cohort. For the lipid-PGS PheWAS, each PGS was inverse normalized prior to analysis and lipid levels were corrected for statin use. The MVP samples used for the PheWAS analysis were not included in the GWAS meta-analysis [24].

Similarly, we extracted all multi-ancestry autosomal index variants for all lipid traits from the same European ancestry subset of the UK Biobank and MVP and performed a single-variant PheWAS association analysis per cohort. Additionally, we performed a single-variant PheWAS association analysis in the UK Biobank only with the sex-stratified and X chromosome index variants from the multi-ancestry analysis.

#### Meta-analysis of MVP and the UK Biobank PheWAS results

We combined, via meta-analysis, PheWAS lipid-specific PGS results for all intersecting phecodes and biomarkers between the UK Biobank and MVP (Europeans only) per lipid trait. We used ICD10-based phecodes and manually matched biomarkers to identify intersecting phenotypes between the two datasets. We restricted our meta-analysis to phenotypes that had at least 100 samples (total number for continuous traits or number of cases for binary traits) in each cohort. After the meta-analysis, we excluded phenotypes that had less than 500 combined samples (total number for continuous traits or number of cases for binary traits), to avoid reporting spurious results [103]. That resulted in a total of 773 phenotypes (739 phecodes and 34 biomarkers/measurements). We used both fixed and random effects model for the meta-analysis. We assessed heterogeneity using the *p*-value for Cochran’s *q* and set the level for significant heterogeneity at a Bonferroni threshold (*p*-value ≤ 6.5 × 10^−5^, to account for multiple testing of 773 phenotypes). We report the results from the fixed-effects model for the phenotypes with non-significant heterogeneity and the results from the random effects model for all others. Similarly, we meta-analyzed all index-variant PheWAS results between the UK Biobank and MVP and obtained results for 811 phenotypes and 1750 lipid multi-ancestry index variants, after excluding instances with a combined sample size < 500.

### Lipid index variants with CAD, T2D, and NAFLD datasets

The GWAS meta-analysis results of CAD and T2D were acquired from MVP [62] and DIAGRAM Consortium [61], respectively. For variant rs1229984, the CAD result is from CARDIoGRAM*Plus*C4D meta-analysis [104], as it was not present in the MVP results. The NAFLD GWAS and meta-analysis was performed in the UK Biobank and Michigan Genomics Initiative (MGI). We determined the association of the lipid index variants with CAD, T2D, and NAFLD and aligned the alleles across all the traits to the LDL-lowering allele. We then highlighted the protective lipid coding alleles associated with CAD.

#### GWAS and meta-analysis of NAFLD in the UK Biobank and Michigan Genomics Initiative (MGI)

Individuals with NAFLD were identified using ICD-9 571.8 and ICD-10 K76.0. Individuals with hepatitis, liver cirrhosis, liver abscess, ascites, a liver transplant, hepatomegaly, jaundice, or with abnormal result of serum enzyme levels or a function study of the liver were excluded (exclusion phecodes 70.2, 70.3, 571.51, 571.6, 571.8, 571.81, 572, 573, 573.2, 573.3, 573.5, 573.7, 573.9) [105]. Analysis was performed using SAIGE v43.3 [106]. Analysis in the UK Biobank included white British individuals with batch, sex, birth year, and the first 4 genetic principal components as covariates. A total of 1122 cases and 399,900 controls were included in the analysis. Analysis in MGI included only European-ancestry participants with array version, sex, birth year, and the first 4 genetic principal components as covariates. A total of 2901 cases and 49,098 controls were analyzed. Meta-analysis was performed using METAL with weighting based on the effective sample size calculated as 4/((1/Ncases) + (1/Ncontrols)).

### CAD/T2D colocalization analysis with lipid traits

We used R package coloc v3.2.1 [93] to perform summary statistics-based colocalization via a Bayesian approach and test whether the 5 lipid traits share common genetic causal variants with CAD or T2D. We first defined a window of ± 100 kb around each index variant [24]. Then for each window of the 10 pairs of traits, we ran colocalization with default parameters using those SNPs present in both datasets. A colocalization posterior probability of PP4 > 0.8 was used to define those loci that show significant colocalization.

## Supplementary Information

**Additional file 1: Table S1.** Characteristics of contributing cohorts (as provided by each participating cohort).

**Additional file 2: Table S2.** Association results for the multi-ancestry index SNPs with the gene prioritization.

**Additional file 3: Figure S1.** Summary of prioritizing genes for A. Mendelian and B. mouse model genes separately by trait.

**Additional file 4: Table S3.** Text mining results for the PoPS+ prioritized genes.

**Additional file 5: Table S4.** Frequency of lipid-related publications for the PoPS+ prioritized genes.

**Additional file 6: Figure S2.** Frequency distribution of the lipid-related publications for both high confidence genes and the baseline genes.

**Additional file 7: Table S5.** Lookup of all prioritized lipid genes in the Therapeutic Target Database 2022.

**Additional file 8: Table S6.** Association of lipid index variants with CAD, T2D and NAFLD.

**Additional file 9: Figure S3.** Lipid traits – tissue/cell type associations estimated by DESE according to GTEx gene-level and GTEx transcript-level selective expression.

**Additional file 10: Table S7.** DESE phenotype-tissue association results using both GTEx gene-level and transcript-level selective expression.

**Additional file 11: Figure S4.** Comparison of PheWAS results in UKB and MVP for the LDL-C PGS, HDL-C PGS, TC PGS, TG PGS and nonHDL-C PGS.

**Additional file 12: Table S8.** PheWAS UKB-MVP meta-analysis results for each lipid PGS.

**Additional file 13: Figure S5.** PheWAS meta-analysis results for the trans-ethnic HDL-C PGS in UK Biobank and MVP.

**Additional file 14: Figure S6.** PheWAS meta-analysis results for the trans-ethnic TC PGS in UK Biobank and MVP.

**Additional file 15: Figure S7.** PheWAS meta-analysis results for the trans-ethnic TG PGS in UK Biobank and MVP.

**Additional file 16: Figure S8.** PheWAS meta-analysis results for the trans-ethnic nonHDL-C PGS in UK Biobank and MVP.

**Additional file 17: Table S9.** PheWAS UKB-MVP meta-analysis results for each index lipid variant at Bonferroni threshold for multiple testing *p*<=3.5e-8)

**Additional file 18: Table S10.** Lambda GC values across minor allele frequency bins for sex-specific meta-analyses.

**Additional file 19: Table S11.** Significant female-specific multi-ancestry meta-analysis results.

**Additional file 20: Table S12.** Significant male-specific multi-ancestry meta-analysis results.

**Additional file 21: Table S13.** Characteristics of replication cohorts (as provided by each participating cohort).

**Additional file 22: Table S14.** Test for difference in effects for index variants from sex-stratified meta-analysis.

**Additional file 23: Table S15.** Comparison of the sex-specific effects.

**Additional file 24: Figure S9.** Comparison of effect size estimates between males and females for index variants showing a significant difference in effect size between sexes.

**Additional file 25: Table S16.** Comparison of effect size estimates for sex-stratified analysis in the replication cohorts.

**Additional file 26: Figure S10.** Comparison of effect sizes for trans-ancestry index variants excluding cholesterol-lowering medication.

**Additional file 27: Table S17.** Sex-stratified effect sizes in UK Biobank considering all individuals or only those not on cholesterol lowering medications.

**Additional file 28: Table S18.** Sex-participation association of the variants with significant sex-specific lipid results.

**Additional file 29: Table S19.** Comparison of sex-stratified effect sizes in the UK Biobank for BMI, waist hip ratio adjusted for BMI, alcohol use, and smoking status.

**Additional file 30: Table S20.** Colocalization results for the sex-specific loci.

**Additional file 31: Supplementary Note.** Supplementary Note and Cohort Acknowledgments.

**Additional file 32: Table S21.** Significant X chromosome results in the sex-combined and sex-stratified analysis and replication.

**Additional file 33: Table S22.** Mouse genes with lipid phenotypes (silver set).

Additional file 34. Review history.

## Data Availability

The GWAS meta-analysis results (including both ancestry-specific and trans-ancestry analyses) and risk score weights are available at: http://csg.sph.umich.edu/willer/public/glgc-lipids2021 [107]. A web browser displaying the gene prioritization and PheWAS results is available at https://hugeamp.org:8000/research.html?pageid=GLGC_149 [108]. The optimized trans-ancestry polygenic score weights are deposited within the PGS Catalog (https://www.pgscatalog.org/publication/PGP000230/ [109] and https://www.pgscatalog.org/publication/PGP000366/ [110]. Scripts used for analysis and summary of results are available under the MIT license on this GitHub repository: https://github.com/Global-Lipids-Genetics [111]. The version of source code used in the manuscript is deposited in Zenodo: https://doi.org/10.5281/zenodo.7130299 [112].
