# Supplementary material for: Implicating genes, pleiotropy, and sexual dimorphism at blood lipid loci through multi-ancestry meta-analysis
Source: Genome Biol. 2022 Dec 27;23:268. doi: 10.1186/s13059-022-02837-1 (PMC9793579; doi:10.1186/s13059-022-02837-1)
Supplement: Supplementary file 3 — Additional file 3: Figure S1. Summary of prioritizing genes for A. Mendelian and B. mouse model genes separately by trait. [file 13059_2022_2837_MOESM3_ESM.pdf]

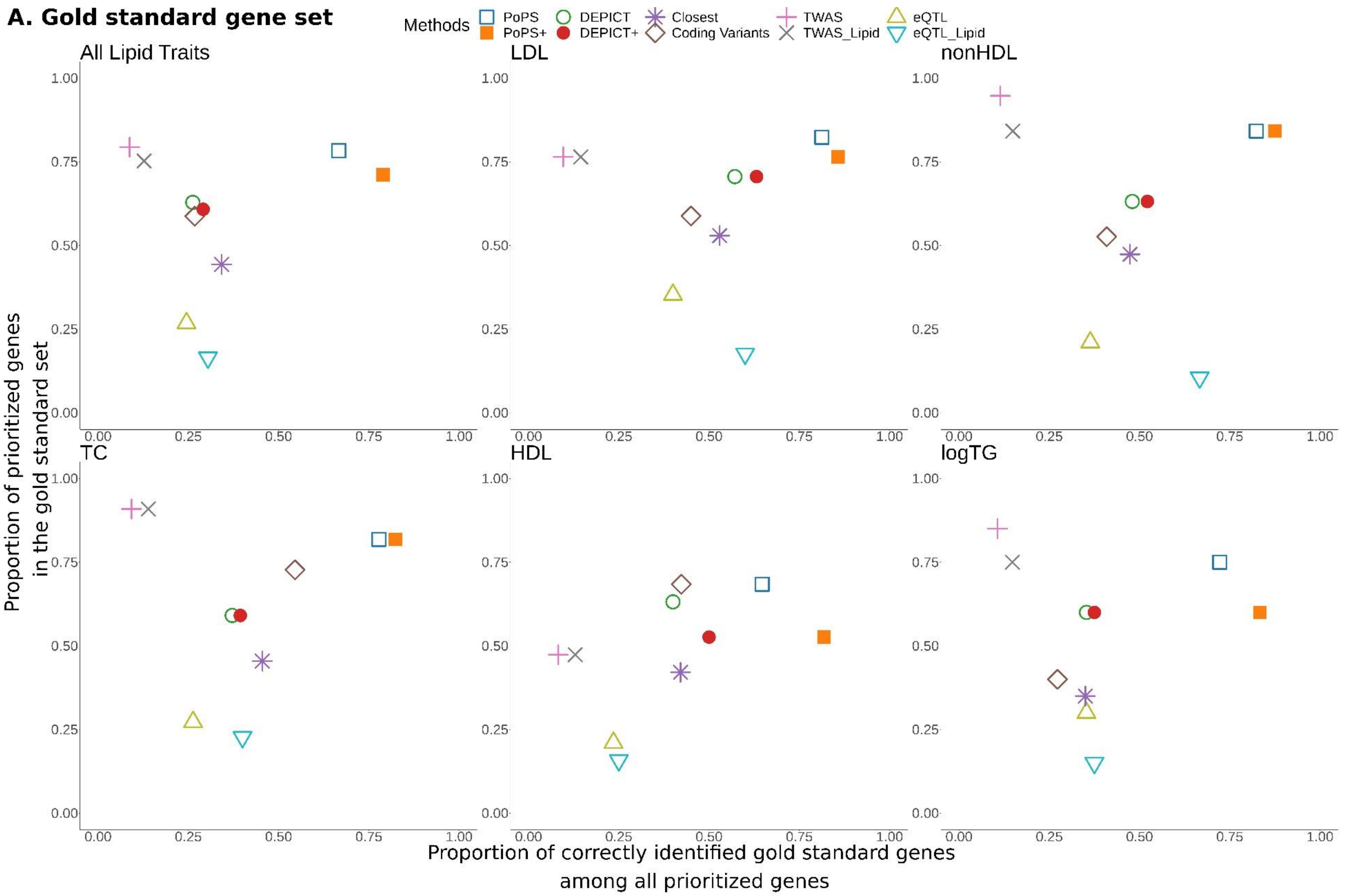
A. Gold standard gene set
Methods
PoPS
PoPS+
DEPICT
DEPICT+
Closest
Coding Variants
TWAS
TWAS_Lipid
eQTL
eQTL_Lipid
All Lipid Traits
LDL
nonHDL
TC
HDL
logTG
0.00
0.25
0.50
0.75
1.00
Proportion of prioritized genes in the gold standard set
Proportion of correctly identified gold standard genes among all prioritized genes

B. Silver standard gene set

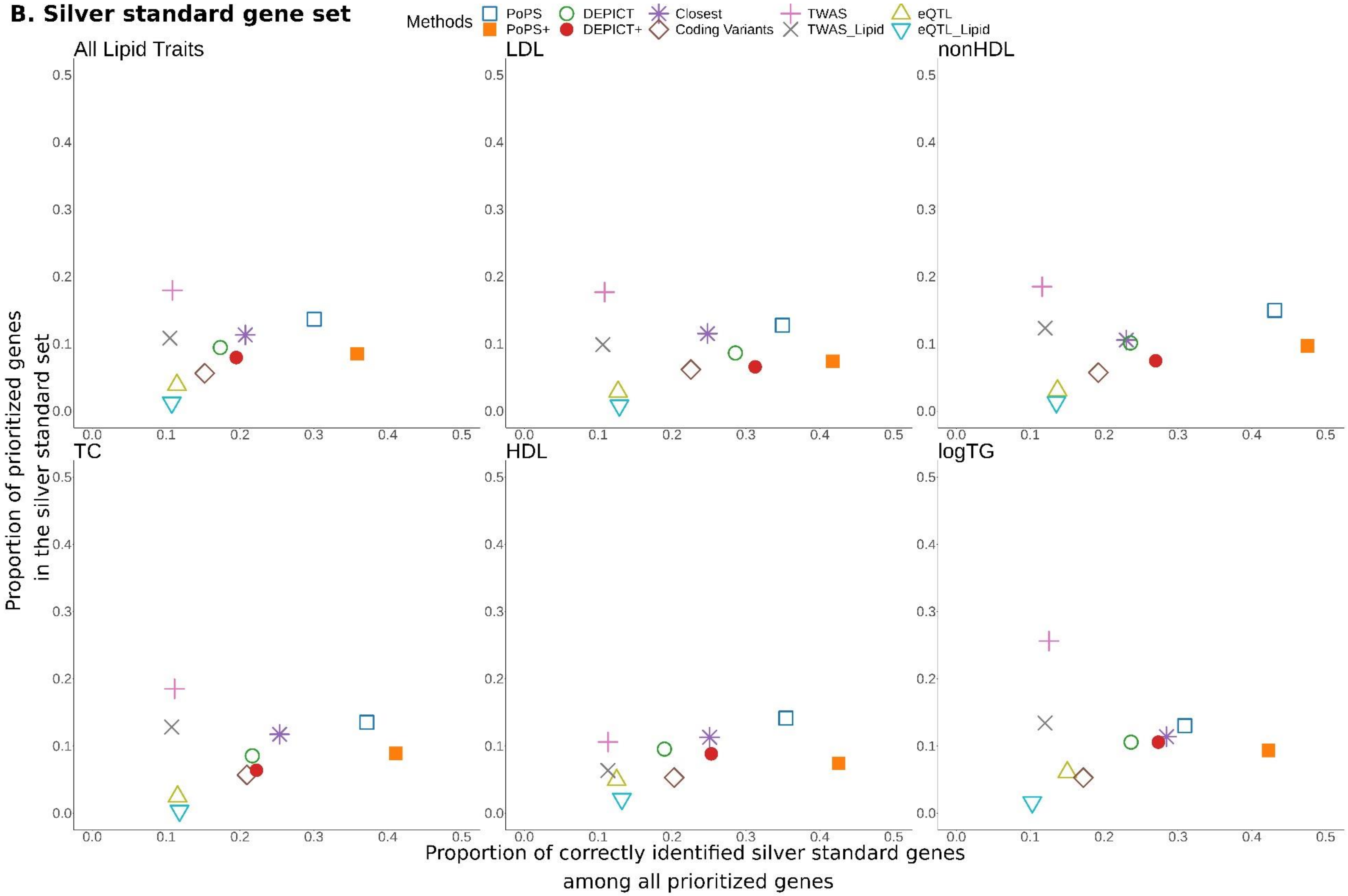

Figure S1. **Summary of prioritizing genes for A. Mendelian and B. mouse model genes separately by trait.**

We determined the proportion of prioritized genes in the gold standard set (y-axis) and proportion of correctly identified gold standard genes among all prioritized genes (x-axis) for each gene prioritization methods utilizing the gold-standard (**A**) and silver-standard (**B**) gene sets. We calculated the overall proportions and separately by trait.
