# Supplementary material for: Implicating genes, pleiotropy, and sexual dimorphism at blood lipid loci through multi-ancestry meta-analysis
Source: Genome Biol. 2022 Dec 27;23:268. doi: 10.1186/s13059-022-02837-1 (PMC9793579; doi:10.1186/s13059-022-02837-1)
Supplement: Supplementary file 6 — Additional file 6: Figure S2. Frequency distribution of the lipid-related publications for both high confidence genes and the baseline genes. [file 13059_2022_2837_MOESM6_ESM.pdf]

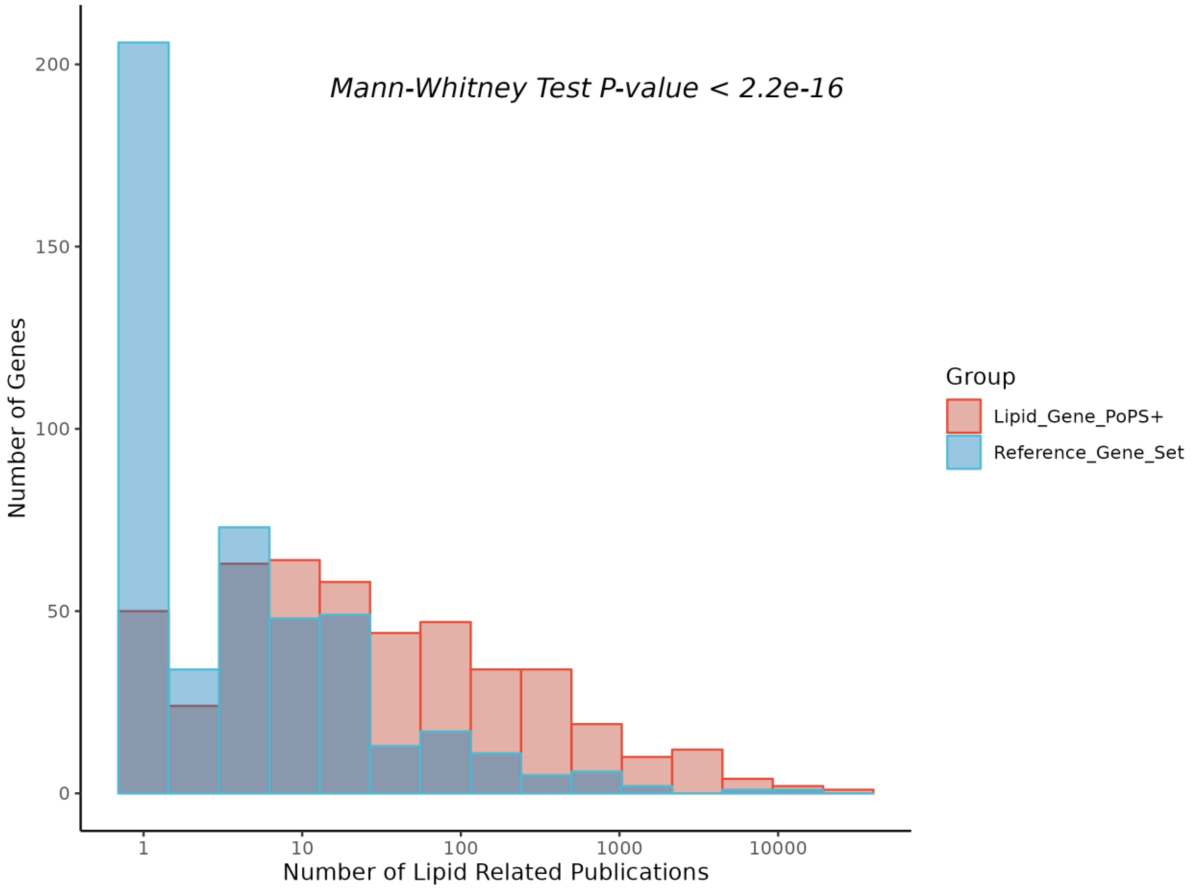

Figure S2. Frequency distribution of the lipid-related publications for both high confidence genes and the baseline genes. The A Mann-Whitney U test showed that there was a significant difference (W = 52353, p-value < 2.2e-16) between the high confidence gene group compared to the baseline group. The exact numbers of lipid-related publications for the high confidence prioritized genes can be found in **Additional file 7: Table S5**.
