# Supplementary material for: Implicating genes, pleiotropy, and sexual dimorphism at blood lipid loci through multi-ancestry meta-analysis
Source: Genome Biol. 2022 Dec 27;23:268. doi: 10.1186/s13059-022-02837-1 (PMC9793579; doi:10.1186/s13059-022-02837-1)
Supplement: Supplementary file 9 — Additional file 9: Figure S3. Lipid traits – tissue/cell type associations estimated by DESE according to GTEx gene-level and GTEx transcript-level selective expression. [file 13059_2022_2837_MOESM9_ESM.pdf]

A

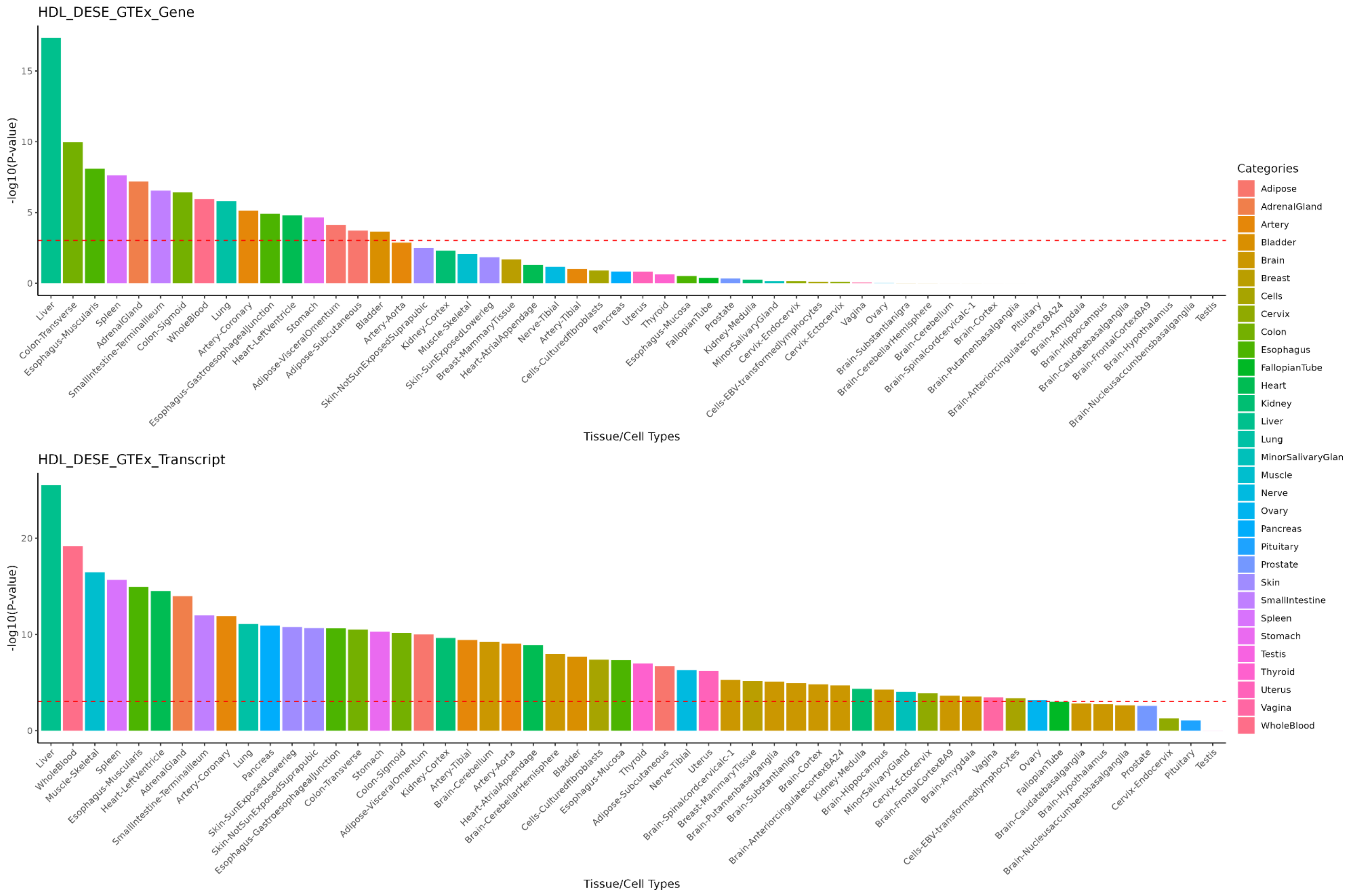
HDL_DESE_GTEx_Gene
-log10(P-value)
Tissue/Cell Types
HDL_DESE_GTEx_Transcript
-log10(P-value)
Tissue/Cell Types
Categories
Adipose
AdrenalGland
Artery
Bladder
Brain
Breast
Cells
Cervix
Colon
Esophagus
FallopianTube
Heart
Kidney
Liver
Lung
MinorSalivaryGlan
Muscle
Nerve
Ovary
Pancreas
Pituitary
Prostate
Skin
SmallIntestine
Spleen
Stomach
Testis
Thyroid
Uterus
Vagina
WholeBlood

B

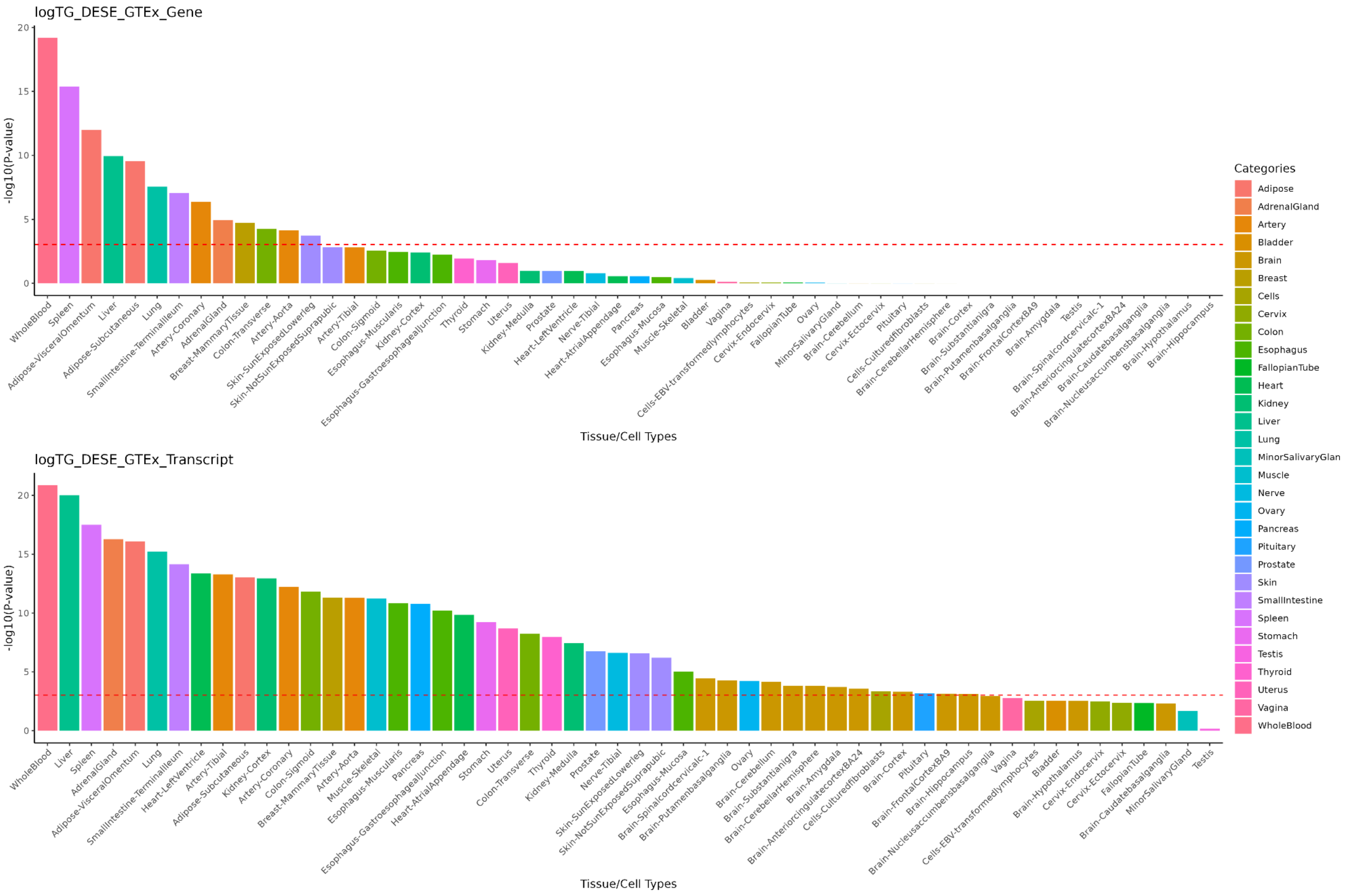

logTG_DESE_GTEx_Gene
-log10(P-value)
Tissue/Cell Types
logTG_DESE_GTEx_Transcript
-log10(P-value)
Tissue/Cell Types
Categories
Adipose
AdrenalGland
Artery
Bladder
Brain
Breast
Cells
Cervix
Colon
Esophagus
FallopianTube
Heart
Kidney
Liver
Lung
MinorSalivaryGlan
Muscle
Nerve
Ovary
Pancreas
Pituitary
Prostate
Skin
SmallIntestine
Spleen
Stomach
Testis
Thyroid
Uterus
Vagina
WholeBlood

C

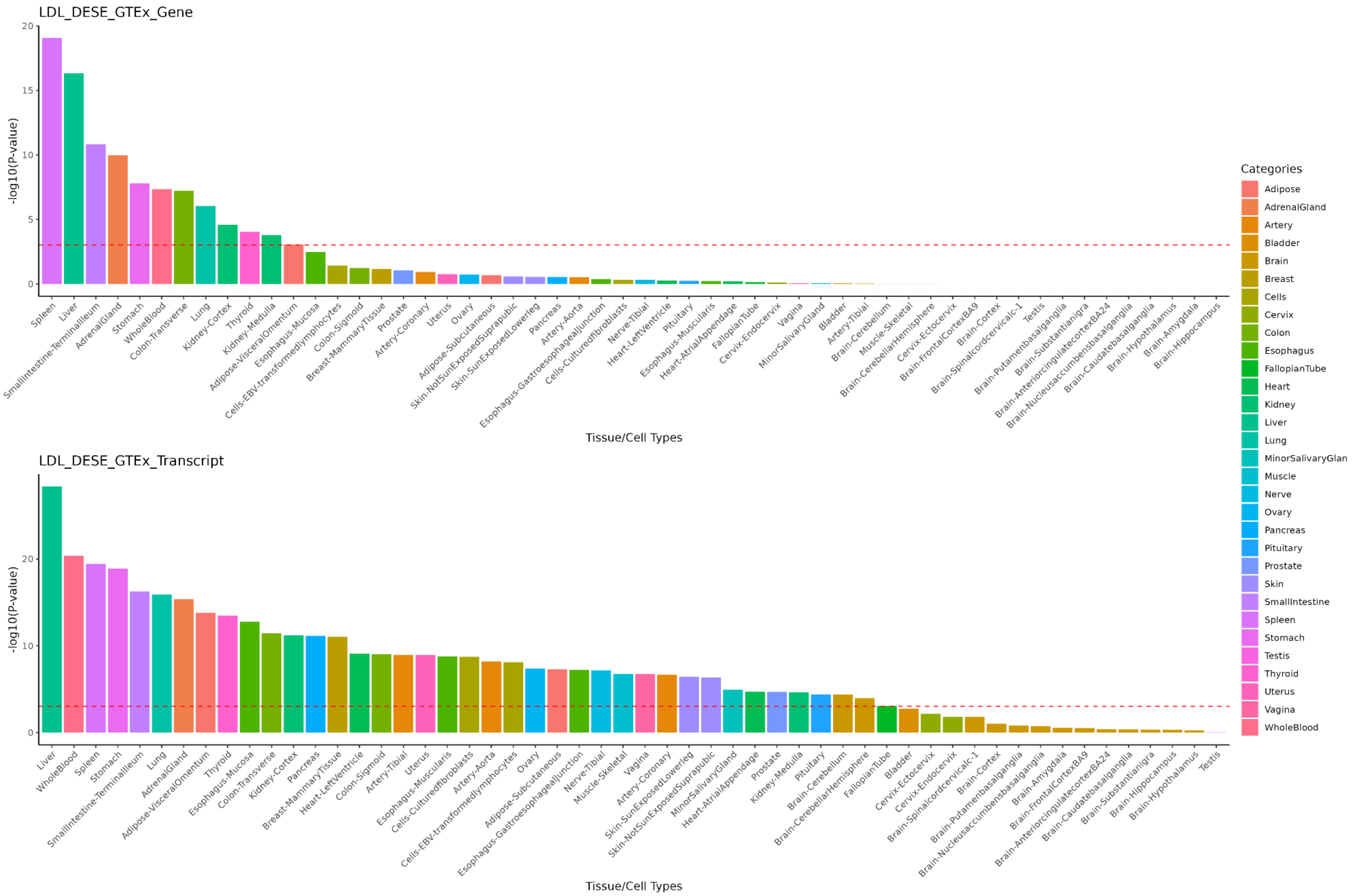
LDL_DESE_GTEx_Gene
-log10(P-value)
0
5
10
15
20
Spleen
Liver
SmallIntestine-TerminalIleum
AdrenalGland
Stomach
WholeBlood
Colon-Transverse
Lung
Kidney-Cortex
Thyroid
Kidney-Medulla
Adipose-VisceralOmentum
Esophagus-Mucosa
Cells-EBV-transformedlymphocytes
Colon-Sigmoid
Breast-MammaryTissue
Prostate
Artery-Coronary
Uterus
Ovary
Adipose-Subcutaneous
Skin-NotSunExposedSuprapubic
Skin-SunExposedLowerleg
Pancreas
Artery-Aorta
Esophagus-GastroesophagealJunction
Cells-Culturedfibroblasts
Nerve-Tibial
Heart-LeftVentricle
Pituitary
Esophagus-Muscularis
Heart-AtrialAppendage
FallopianTube
Cervix-Endocervix
Vagina
MinorSalivaryGland
Bladder
Artery-Tibial
Brain-Cerebellum
Muscle-Skeletal
Brain-CerebellarHemisphere
Cervix-Ectocervix
Brain-FrontalCortexBA9
Brain-Cortex
Brain-Spinalcordcervicalc-1
Testis
Brain-Putamenbasalganglia
Brain-Substantianigra
Brain-AnteriorcingulatecortexBA24
Brain-Nucleusaccumbensbasalganglia
Brain-Caudatebasalganglia
Brain-Hypothalamus
Brain-Amygdala
Brain-Hippocampus
Tissue/Cell Types
LDL_DESE_GTEx_Transcript
-log10(P-value)
0
10
20
Liver
WholeBlood
Spleen
Stomach
SmallIntestine-TerminalIleum
Lung
AdrenalGland
Adipose-VisceralOmentum
Thyroid
Esophagus-Mucosa
Colon-Transverse
Kidney-Cortex
Pancreas
Breast-MammaryTissue
Heart-LeftVentricle
Colon-Sigmoid
Artery-Tibial
Uterus
Esophagus-Muscularis
Cells-Culturedfibroblasts
Artery-Aorta
Cells-EBV-transformedlymphocytes
Ovary
Adipose-Subcutaneous
Esophagus-GastroesophagealJunction
Nerve-Tibial
Muscle-Skeletal
Vagina
Artery-Coronary
Skin-SunExposedLowerleg
Skin-NotSunExposedSuprapubic
MinorSalivaryGland
Heart-AtrialAppendage
Prostate
Kidney-Medulla
Pituitary
Brain-Cerebellum
Brain-CerebellarHemisphere
FallopianTube
Bladder
Cervix-Ectocervix
Cervix-Endocervix
Brain-Spinalcordcervicalc-1
Brain-Cortex
Brain-Putamenbasalganglia
Brain-Nucleusaccumbensbasalganglia
Brain-Amygdala
Brain-FrontalCortexBA9
Brain-AnteriorcingulatecortexBA24
Brain-Caudatebasalganglia
Brain-Substantianigra
Brain-Hippocampus
Brain-Hypothalamus
Testis
Tissue/Cell Types
Categories
Adipose
AdrenalGland
Artery
Bladder
Brain
Breast
Cells
Cervix
Colon
Esophagus
FallopianTube
Heart
Kidney
Liver
Lung
MinorSalivaryGlan
Muscle
Nerve
Ovary
Pancreas
Pituitary
Prostate
Skin
SmallIntestine
Spleen
Stomach
Testis
Thyroid
Uterus
Vagina
WholeBlood

D

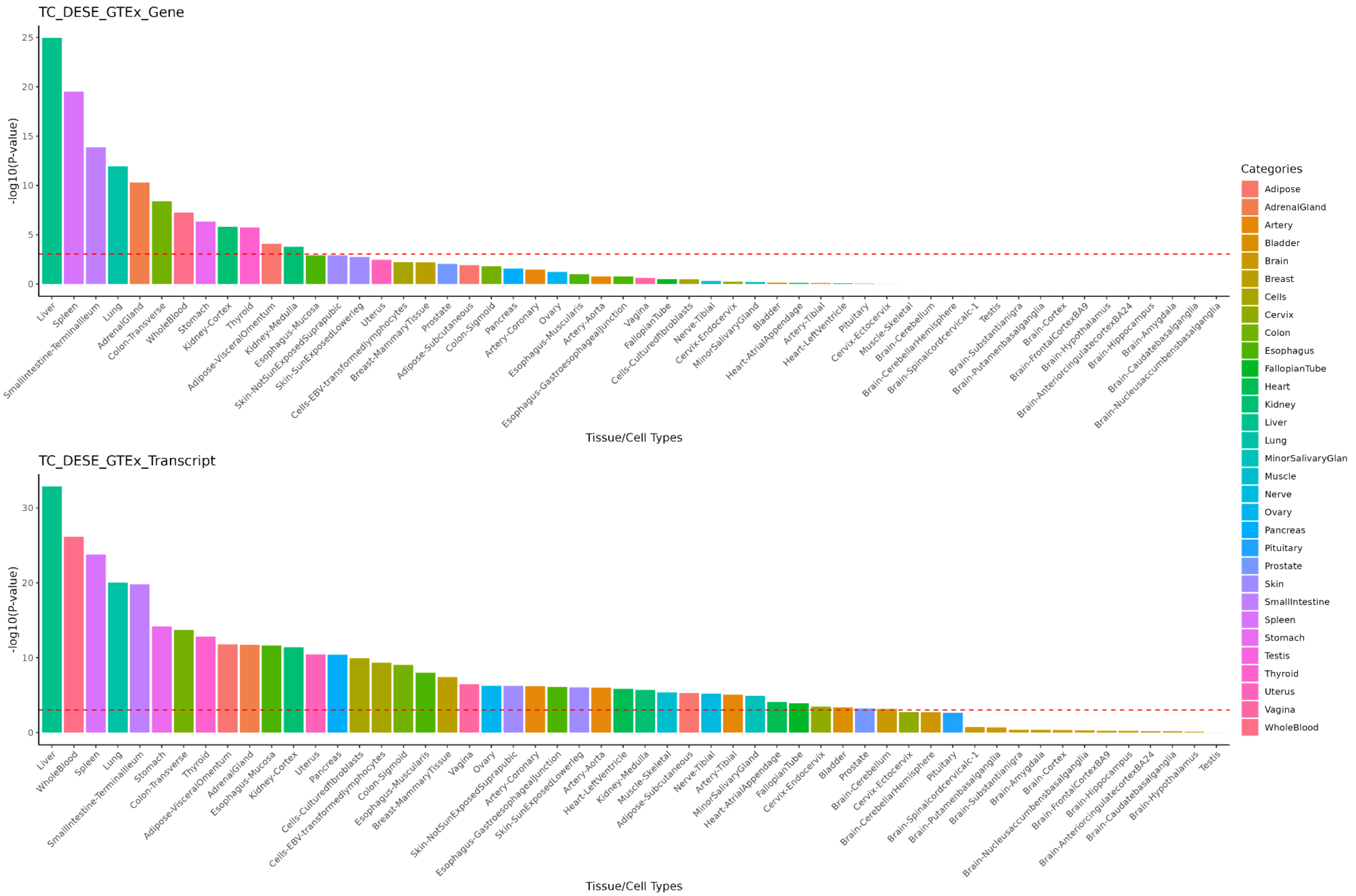
TC_DESE_GTEx_Gene
-log10(P-value)
0
5
10
15
20
25
Liver
Spleen
SmallIntestine-TerminalIleum
Lung
AdrenalGland
Colon-Transverse
WholeBlood
Stomach
Kidney-Cortex
Thyroid
Adipose-VisceralOmentum
Kidney-Medulla
Esophagus-Mucosa
Skin-NotSunExposedSuprapubic
Skin-SunExposedLowerleg
Uterus
Cells-EBV-transformedlymphocytes
Breast-MammaryTissue
Prostate
Adipose-Subcutaneous
Colon-Sigmoid
Pancreas
Artery-Coronary
Ovary
Esophagus-Muscularis
Artery-Aorta
Esophagus-GastroesophagealJunction
Vagina
FallopianTube
Cells-Culturedfibroblasts
Nerve-Tibial
Cervix-Endocervix
MinorSalivaryGland
Bladder
Heart-AtrialAppendage
Artery-Tibial
Heart-LeftVentricle
Pituitary
Cervix-Ectocervix
Muscle-Skeletal
Brain-Cerebellum
Brain-CerebellarHemisphere
Brain-Spinalcordcervicalc-1
Testis
Brain-Substantianigra
Brain-Putamenbasalganglia
Brain-Cortex
Brain-FrontalCortexBA9
Brain-Hypothalamus
Brain-AnteriorcingulatecortexBA24
Brain-Hippocampus
Brain-Amygdala
Brain-Caudatebasalganglia
Brain-Nucleusaccumbensbasalganglia
Tissue/Cell Types
TC_DESE_GTEx_Transcript
-log10(P-value)
0
10
20
30
Liver
WholeBlood
Spleen
Lung
SmallIntestine-TerminalIleum
Stomach
Colon-Transverse
Thyroid
Adipose-VisceralOmentum
AdrenalGland
Esophagus-Mucosa
Kidney-Cortex
Uterus
Pancreas
Cells-Culturedfibroblasts
Cells-EBV-transformedlymphocytes
Colon-Sigmoid
Esophagus-Muscularis
Breast-MammaryTissue
Vagina
Ovary
Skin-NotSunExposedSuprapubic
Artery-Coronary
Esophagus-GastroesophagealJunction
Skin-SunExposedLowerleg
Artery-Aorta
Heart-LeftVentricle
Kidney-Medulla
Muscle-Skeletal
Adipose-Subcutaneous
Nerve-Tibial
Artery-Tibial
MinorSalivaryGland
Heart-AtrialAppendage
FallopianTube
Cervix-Endocervix
Bladder
Prostate
Brain-Cerebellum
Cervix-Ectocervix
Brain-CerebellarHemisphere
Pituitary
Brain-Spinalcordcervicalc-1
Brain-Putamenbasalganglia
Brain-Substantianigra
Brain-Amygdala
Brain-Cortex
Brain-Nucleusaccumbensbasalganglia
Brain-FrontalCortexBA9
Brain-Hippocampus
Brain-AnteriorcingulatecortexBA24
Brain-Caudatebasalganglia
Brain-Hypothalamus
Testis
Tissue/Cell Types
Categories
Adipose
AdrenalGland
Artery
Bladder
Brain
Breast
Cells
Cervix
Colon
Esophagus
FallopianTube
Heart
Kidney
Liver
Lung
MinorSalivaryGlan
Muscle
Nerve
Ovary
Pancreas
Pituitary
Prostate
Skin
SmallIntestine
Spleen
Stomach
Testis
Thyroid
Uterus
Vagina
WholeBlood

E

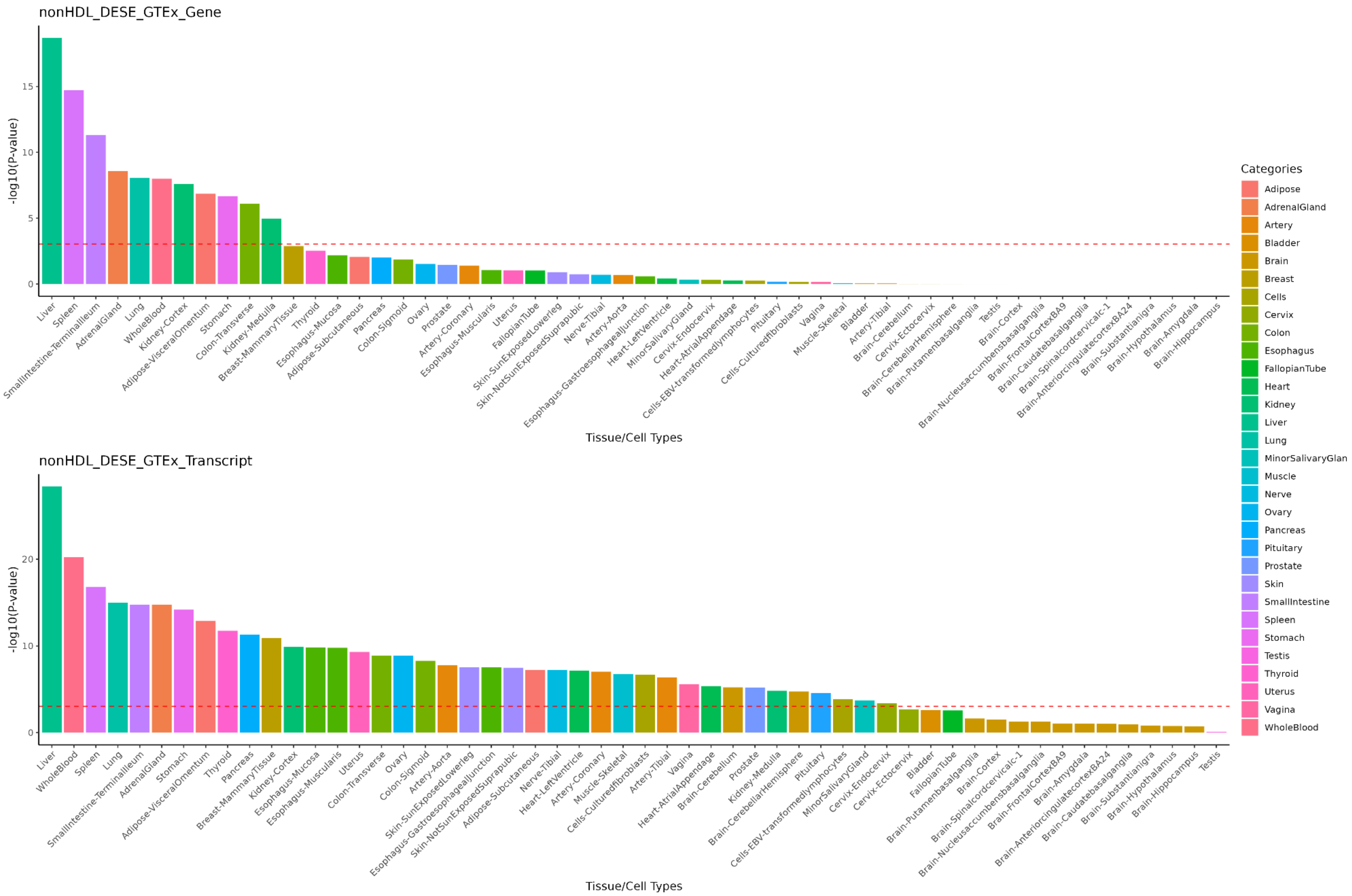
nonHDL_DESE_GTEx_Gene
-log10(P-value)
0
5
10
15
Liver
Spleen
SmallIntestine-TerminalIleum
AdrenalGland
Lung
WholeBlood
Kidney-Cortex
Adipose-VisceralOmentum
Stomach
Colon-Transverse
Kidney-Medulla
Breast-MammaryTissue
Thyroid
Esophagus-Mucosa
Adipose-Subcutaneous
Pancreas
Colon-Sigmoid
Ovary
Prostate
Artery-Coronary
Esophagus-Muscularis
Uterus
FallopianTube
Skin-SunExposedLowerleg
Skin-NotSunExposedSuprapubic
Nerve-Tibial
Artery-Aorta
Esophagus-GastroesophagealJunction
Heart-LeftVentricle
MinorSalivaryGland
Cervix-Endocervix
Heart-AtrialAppendage
Cells-EBV-transformedlymphocytes
Pituitary
Cells-Culturedfibroblasts
Vagina
Muscle-Skeletal
Bladder
Artery-Tibial
Brain-Cerebellum
Cervix-Ectocervix
Brain-CerebellarHemisphere
Brain-Putamenbasalganglia
Testis
Brain-Cortex
Brain-Nucleusaccumbensbasalganglia
Brain-FrontalCortexBA9
Brain-Caudatebasalganglia
Brain-Spinalcordcervicalc-1
Brain-AnteriorcingulatecortexBA24
Brain-Substantianigra
Brain-Hypothalamus
Brain-Amygdala
Brain-Hippocampus
Tissue/Cell Types
nonHDL_DESE_GTEx_Transcript
-log10(P-value)
0
10
20
Liver
WholeBlood
Spleen
Lung
SmallIntestine-TerminalIleum
AdrenalGland
Stomach
Adipose-VisceralOmentum
Thyroid
Pancreas
Breast-MammaryTissue
Kidney-Cortex
Esophagus-Mucosa
Esophagus-Muscularis
Uterus
Colon-Transverse
Ovary
Colon-Sigmoid
Artery-Aorta
Skin-SunExposedLowerleg
Esophagus-GastroesophagealJunction
Skin-NotSunExposedSuprapubic
Adipose-Subcutaneous
Nerve-Tibial
Heart-LeftVentricle
Artery-Coronary
Muscle-Skeletal
Cells-Culturedfibroblasts
Artery-Tibial
Vagina
Heart-AtrialAppendage
Brain-Cerebellum
Prostate
Kidney-Medulla
Brain-CerebellarHemisphere
Pituitary
Cells-EBV-transformedlymphocytes
MinorSalivaryGland
Cervix-Endocervix
Cervix-Ectocervix
Bladder
FallopianTube
Brain-Putamenbasalganglia
Brain-Cortex
Brain-Spinalcordcervicalc-1
Brain-Nucleusaccumbensbasalganglia
Brain-FrontalCortexBA9
Brain-Amygdala
Brain-AnteriorcingulatecortexBA24
Brain-Caudatebasalganglia
Brain-Substantianigra
Brain-Hypothalamus
Brain-Hippocampus
Testis
Tissue/Cell Types
Categories
Adipose
AdrenalGland
Artery
Bladder
Brain
Breast
Cells
Cervix
Colon
Esophagus
FallopianTube
Heart
Kidney
Liver
Lung
MinorSalivaryGlan
Muscle
Nerve
Ovary
Pancreas
Pituitary
Prostate
Skin
SmallIntestine
Spleen
Stomach
Testis
Thyroid
Uterus
Vagina
WholeBlood

Figure S3. **Lipid traits – tissue/cell type associations estimated by DESE according to GTEx gene-level and GTEx transcript-level selective expression.**

We performed phenotype-tissue association tests in five lipid traits: (**A**) HDL-C, (**B**) TG, (**C**) LDL-C, (**D**) TC, and (**E**) nonHDL-C with 54 GTEx tissues. For each lipid traits, the upper panel showed the estimated driver tissues according to GTEx gene-level selective expression and the bottom panel showed estimated driver tissues according to GTEx transcript-level selective expression. The tissues were ranked by their association significance (-log10(P-value)) with each lipid traits. The red horizontal line denoted the significance threshold using a Bonferroni correction.
