# Supplementary material for: Implicating genes, pleiotropy, and sexual dimorphism at blood lipid loci through multi-ancestry meta-analysis
Source: Genome Biol. 2022 Dec 27;23:268. doi: 10.1186/s13059-022-02837-1 (PMC9793579; doi:10.1186/s13059-022-02837-1)
Supplement: Supplementary file 11 — Additional file 11: Figure S4. Comparison of PheWAS results in UKB and MVP for the LDL-C PGS, HDL-C PGS, TC PGS, TG PGS and nonHDL-C PGS. [file 13059_2022_2837_MOESM11_ESM.pdf]

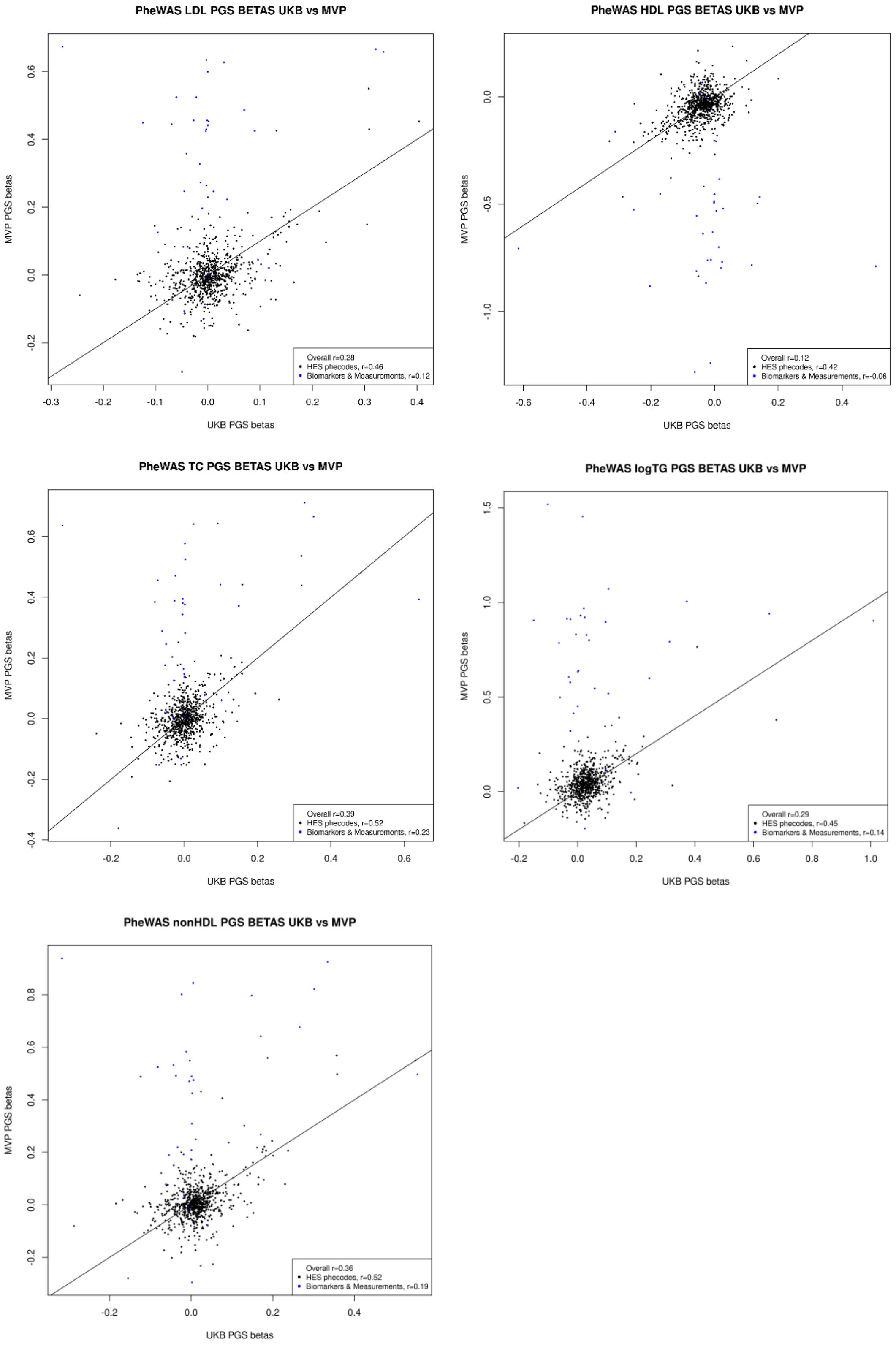

Figure S4. **Comparison of PheWAS results in UKB and MVP for the LDL-C PGS, HDL-C PGS, TC PGS, TG PGS and nonHDL-C PGS.** Effect sizes (betas) for the association of each PGS with each phecode and biomarker are plotted for UK Biobank (x-axis) versus MVP (y-axis). Black points represent ICD10-derived phecodes and blue points represent biomarkers.
