# Supplementary figures and images for: Implicating genes, pleiotropy, and sexual dimorphism at blood lipid loci through multi-ancestry meta-analysis
Source: Genome Biol. 2022 Dec 27;23:268. doi: 10.1186/s13059-022-02837-1 (PMC9793579; doi:10.1186/s13059-022-02837-1)

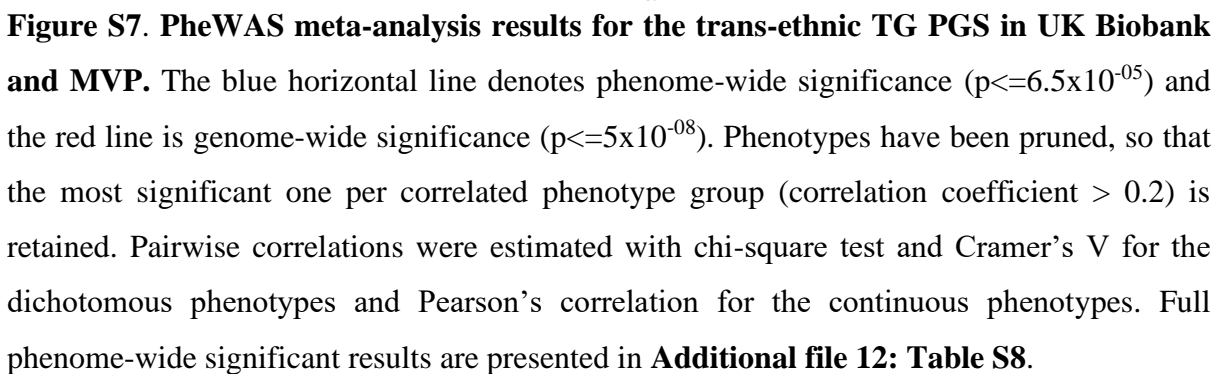

Supplement: Supplementary file 15 — Additional file 15: Figure S7. PheWAS meta-analysis results for the trans-ethnic TG PGS in UK Biobank and MVP. [file 13059_2022_2837_MOESM15_ESM.pdf]
