# Supplementary material for: Implicating genes, pleiotropy, and sexual dimorphism at blood lipid loci through multi-ancestry meta-analysis
Source: Genome Biol. 2022 Dec 27;23:268. doi: 10.1186/s13059-022-02837-1 (PMC9793579; doi:10.1186/s13059-022-02837-1)
Supplement: Supplementary file 16 — Additional file 16: Figure S8. PheWAS meta-analysis results for the trans-ethnic nonHDL-C PGS in UK Biobank and MVP. [file 13059_2022_2837_MOESM16_ESM.pdf]

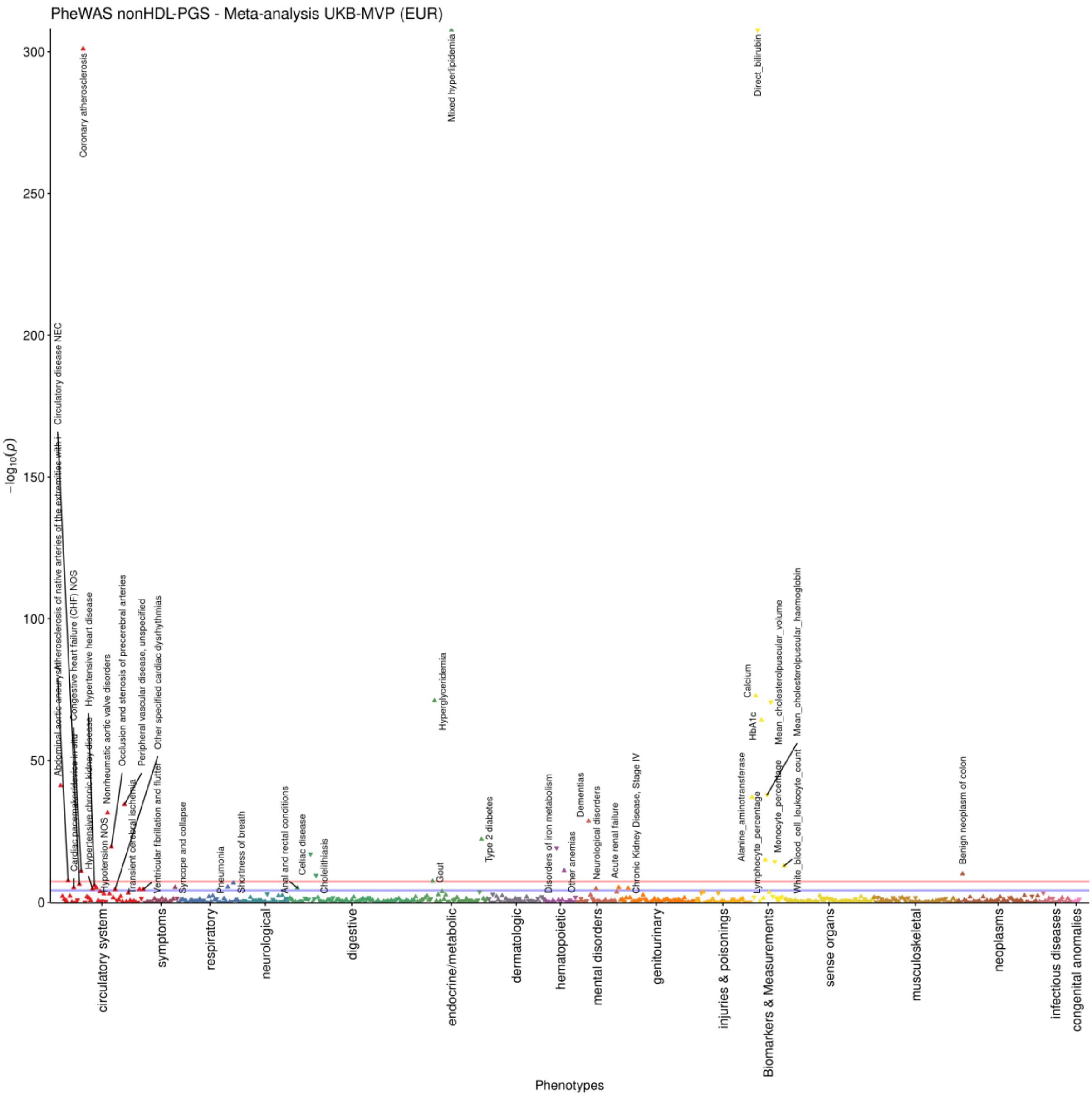

Figure S8. **PheWAS meta-analysis results for the trans-ethnic nonHDL-C PGS in UK Biobank and MVP.** The blue horizontal line denotes phenome-wide significance ($p<=6.5x10^{-05}$) and the red line is genome-wide significance ($p<=5x10^{-08}$). Phenotypes have been pruned, so that the most significant one per correlated phenotype group (correlation coefficient > 0.2) is retained. Pairwise correlations were estimated with chi-square test and Cramer's V for the dichotomous phenotypes and Pearson's correlation for the continuous phenotypes. Full phenome-wide significant results are presented in **Additional file 12: Table S8**.
