# Supplementary material for: Implicating genes, pleiotropy, and sexual dimorphism at blood lipid loci through multi-ancestry meta-analysis
Source: Genome Biol. 2022 Dec 27;23:268. doi: 10.1186/s13059-022-02837-1 (PMC9793579; doi:10.1186/s13059-022-02837-1)
Supplement: Supplementary file 24 — Additional file 24: Figure S9. Comparison of effect size estimates between males and females for index variants showing a significant difference in effect size between sexes. [file 13059_2022_2837_MOESM24_ESM.pdf]

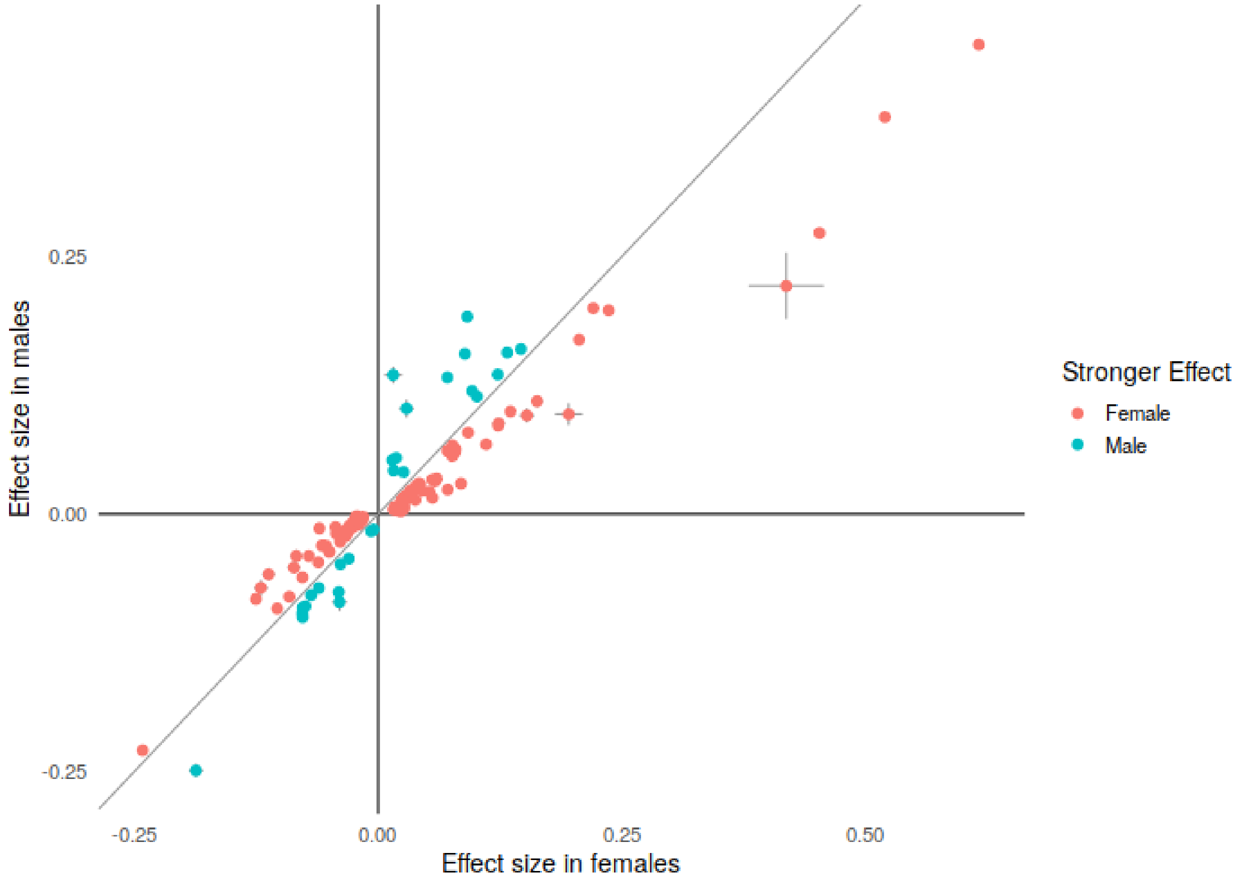

Figure S9. **Comparison of effect size estimates between males and females for index variants showing a significant difference in effect size between sexes.** Effect size estimates are from trans-ancestry meta-analysis in each sex and were more often stronger in females relative to males.
