# Supplementary material for: Implicating genes, pleiotropy, and sexual dimorphism at blood lipid loci through multi-ancestry meta-analysis
Source: Genome Biol. 2022 Dec 27;23:268. doi: 10.1186/s13059-022-02837-1 (PMC9793579; doi:10.1186/s13059-022-02837-1)
Supplement: Supplementary file 26 — Additional file 26: Figure S10. Comparison of effect sizes for trans-ancestry index variants excluding cholesterol-lowering medication. [file 13059_2022_2837_MOESM26_ESM.pdf]

A. Females (all index variants)

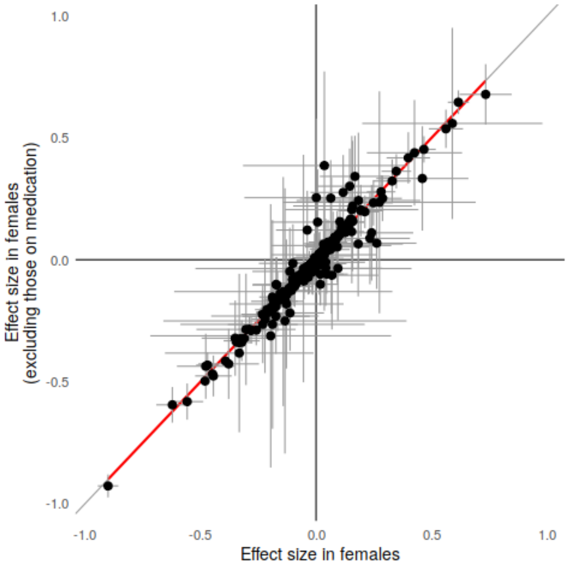

B. Females (only variants with significant difference by sex)

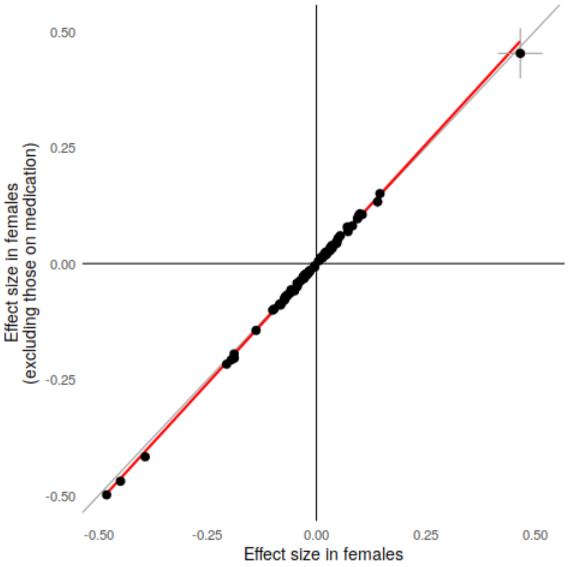

C. Males (all index variants)

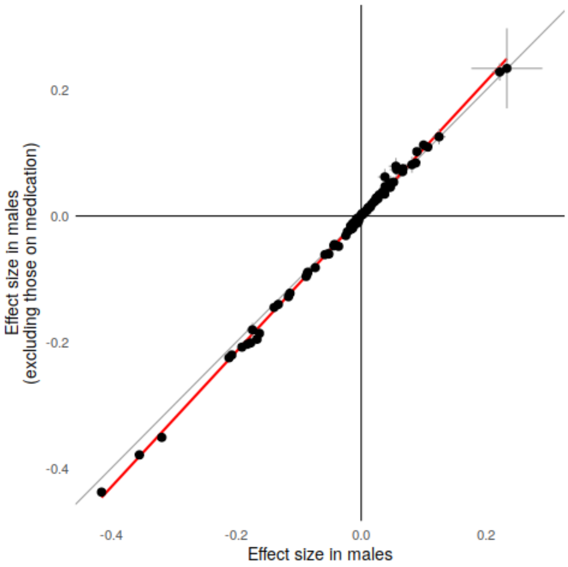

D. Males (only variants with significant difference by sex)

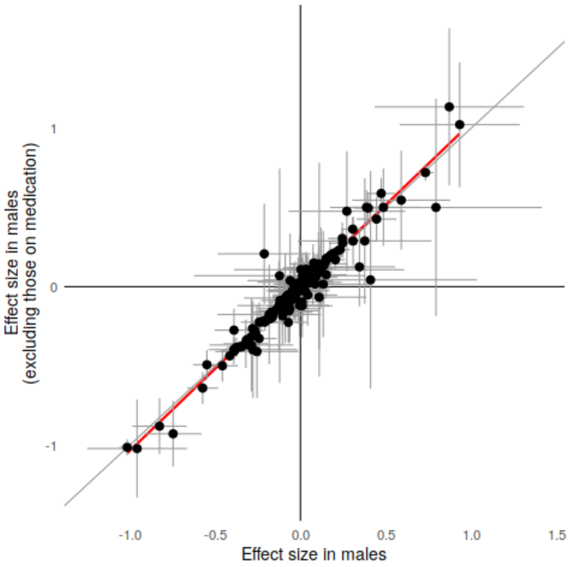

Figure S10. Comparison of effect sizes for trans-ancestry index variants excluding cholesterol-lowering medication. Grey lines show the y=x diagonal while red lines are from linear regression. Effect sizes were slightly larger after excluding individuals on cholesterol-lowering medication.
