# Supplementary material for: Implicating genes, pleiotropy, and sexual dimorphism at blood lipid loci through multi-ancestry meta-analysis
Source: Genome Biol. 2022 Dec 27;23:268. doi: 10.1186/s13059-022-02837-1 (PMC9793579; doi:10.1186/s13059-022-02837-1)
Supplement: Supplementary file 34 — Additional file 34. Review history. [file 13059_2022_2837_MOESM34_ESM.docx]

Review History

**First round of review**

**Reviewer 1**

**Were you able to assess all statistics in the manuscript, including the appropriateness of statistical tests used?**

Yes, and I have assessed the statistics in my report.

**Comments to author:**

This manuscript presents a secondary analysis of association loci for prioritizing genetic factors of blood lipid on autosomes and for discovering loci on X chromosome. The manuscript is well-written generally with clear structure and abundant results supporting their conclusions. It may have broad interest to others in the field. However, this reviewer has several comments which may help improve the manuscript.

1. Given this valuable data with sex infroamtion and sex difference of lipid, it may be interesting to estimate the heritability on X chromosome for males and females.

2. It is necessary to remove reduandent genes associated with lipids due to their variant in LD in such a huge GWAS sample. Probably, the author can try a conditional gene-based analysis proposed by DESE (Genome biology, 2019, 20(1):1-19) . More importantly, DESE can also be used to prioritize the tissues or cell types by the lipid(http://pmglab.top/kggsee), which is a critical factor for the many prororitization analyses by the authors.

3. For the bench-mark setting and the first locus-specific method, falling within 500kb of a credible gene may be too broad.

4. In addition, given the genetic difference between Mendelian and complex diseases, the authors should justify why it is acceptable to use the Mendelian gene for the validation of different methods.

5. What do you mean "protective protein-altering lipid alleles", Pls explain the term briefly.

6. The phenome-wide association scan looks strange and remains association. The MR methods have been wildly used for causal inference among the traits. Why did not the author use MR for the causation analysis?

7. Given there a lot of results this study has produced, it may necessary to make a web paper for readers to quickly search or browse the prioritized genes and corresponding evidences.

8. It is not clear what are the new major findings of this study compared with the previous studies particularly the [ref. 40].

9. A minor point is that Figure 2 has too much empty space.

**Reviewer 2**

**Were you able to assess all statistics in the manuscript, including the appropriateness of statistical tests used?**

Yes, and I have assessed the statistics in my report.

**Comments to author:**

Kanoni et al. present a comprehensive genetic analysis of blood lipids in a multi-ancestry meta-analytic setting. They address three critical issues in lipids genetics: 1. Putative causal gene implication; 2. Utility of genetically-predicted lipid levels; and 3. sex-specific lipids genetic associations. Overall, I find the paper is most clearly written and addresses important issues in lipids genetics and genomics. However, I have a few concerns about some technical aspects of the presented analyses. I hope that the authors can address these issues in the revision.

1. On putative causal gene implication. This is the main emphasis of the paper, and the authors present the results from the six available methods. My concern is that some of the claims in the article diverge to evaluating the various gene implication methods, and the data used in this paper is not designed for this purpose. Firstly, the authors construct a gold and silver set of genes and use them to benchmark the performance of various gene implication methods. I am concerned about how "precision" and "recall" are computed in this process. I don't think the terminologies are appropriate here. Particularly, the "recalls" (they are generally low across methods) are only valid if none of the genes outside the gold/silver set are causal genes, which is too strong an assumption. Secondly, even if we ignore the caveats of defining "precision" and "recall," the cutoffs used by different methods for prioritizing causal genes are seemingly very different and difficult to compare. E.g., the TWAS method controls FDR, the colocalization method controls local FDR, and other methods do not offer type I error control, and the threshold seems arbitrary. On this issue, instead of presenting Figure 1B, the complete precision-recall curve for each method seems more informative and appropriate. Thirdly, can the authors provide some reasoning for the superior performance of PoPS? Specifically, do the training weights (on genomic features) offer any biological insights? If only human_multiple from GTEx data is used, how is its performance compared to the TWAS and the colocalization analysis? How relevant are the mouse gene feature datasets concerning the gold and silver sets, respectively? In summary, I think the current presentation of this analysis opens the door for a series of technical questions like the above. Alternatively, I suggest the authors focus on the common set of causal genes identified by multiple methods and establish their biological relevance (instead of comparing gene implication methods).

2. On lipid PRS associations. The analysis presented in this section shares many common properties with the TWAS analysis. As pointed out by Barfield 2018 Genetic Epi, Mancuso 2019 Nature Genetics, and Hukku 2022 AJHG, some of the signals are simply due to some GWAS hits for lipids and other traits in modest LD. Do the authors also observe such phenomena. If so, do they impact the interpretations?

3. On sex-specific lipids associations. The reasoning of p-values for the sex-stratified analysis is questionable and not convincing. The caveats of using p-values to classify the presence and absence of true associations have been well-known in applications like defining replication successes and identifying tissue-specific eQTLs. Presenting the estimated effects from the stratified analysis and computing some heterogeneity measures would be better evidence for this specific analysis.

4. Some minor details are also needed for the Method section. E.g., the coding variants method uses a 99% credible set. Are the credible sets constructed by single-SNP or fine-mapping analysis? In coloc analysis, are the fine-mapping results of GTEx eQTL used or just the simple single-SNP association results? These details will help the readers to interpret the results presented in the paper.

**Authors Response**

**Point-by-point responses to the reviewers’ comments:**

Reviewer #1:

This manuscript presents a secondary analysis of association loci for prioritizing genetic factors of blood lipid on autosomes and for discovering loci on X chromosome. The manuscript is well-written generally with clear structure and abundant results supporting their conclusions. It may have broad interest to others in the field. However, this reviewer has several comments which may help improve the manuscript.

1. Given this valuable data with sex infroamtion and sex difference of lipid, it may be interesting to estimate the heritability on X chromosome for males and females.

*Response: We agree that the chrX heritability would be interesting to assess. However, the only currently available method for heritability analysis involving chrX that could account for the chrX inactivation is GCTA-GREML[1] and this method can only be applied to individual level data. As the power of our chrX analysis lies in the large overall sample size of our metaanalysis, we cannot harness it for this purpose due to lack of available methodology.*

2. It is necessary to remove reduandent genes associated with lipids due to their variant in LD in such a huge GWAS sample. Probably, the author can try a conditional gene-based analysis proposed by DESE (Genome biology, 2019, 20(1):1-19) . More importantly, DESE can also be used to prioritize the tissues or cell types by the lipid(http://pmglab.top/kggsee), which is a critical factor for the many prororitization analyses by the authors.

*Response: We determined independent loci by physical distance >500kb or genetic distance >0.25cM, whichever one would result in a larger window, followed by a conditional analysis using rareGWAMA[2] to identify index variants that were shadows of nearby, moresignificant associations. We then performed gene prioritization of each of the indexed variants. While the variants are independent, they can point to the same gene.*

*We thank the reviewer for suggesting we use DESE to prioritize tissues or cell types. We have added a section to the manuscript with this information. As expected, liver is a major tissue for lipid metabolism. Furthermore, in a recently published manuscript in the AJHG[3], we explored tissue and cell-type prioritization in detail. In that manuscript, liver tissues also showed strong enrichments across all lipid traits.*

*Text added to the Results:*

*Driver tissues for lipid levels*

*We applied DESE (Driver-tissue Estimation by Selective Expression) [40] to estimate the driver tissues of five lipid traits using both gene-level and transcript-level eQTL summary statistics from GTEx v8 tissues [41]. We identified liver as the top-ranked tissue for HDL-C (gene-level p value = 4.5E-18, transcript-level p value = 3.0E-26), TC (gene-level p value = 1.1E-25, transcript-level p value = 1.4E-33), and non-HDL-C (gene-level p value = 2.0E-19, transcript-level p value = 3.9E-29) based on both gene-level and transcript-level selective expression (Figure S3, Table S7). For LDL-C, we identified the spleen as the top-ranked tissue using GTEx gene-level data (p value = 8.3E-20), while liver was ranked second (p value = 4.8E-17). However, when using GTEx transcript-level data, liver was the top-ranked tissue (p value = 4.3E-29) and second was whole blood (p value = 4.3E-20). The top-ranked tissue for TG according to both GTEx gene-level and transcript-level expression data was whole blood (genelevel p value = 6.4E-20, transcript-level p value = 1.4E-21). Spleen and liver were second according to GTEx gene-level and transcript-level expression data, respectively. The results were consistent with previous knowledge that the liver is a major tissue for lipid metabolism. Transcript-level selective expression provided more statistically significant results for the estimated driver tissues compared to the gene-level selective expression, as reported in the original DESE paper (Jiang et al., 2019).*

*Text added to the Methods:*

*We performed phenotype-tissue association analysis using DESE (Driver-tissue Estimation by Selective Expression) [40]. DESE estimates the causal tissues by selective expression of phenotype-associated genes in GWAS. We used the GWAS summary statistics from the five lipid traits and the GTEx v8 normalized gene-level and transcript-level expression datasets as inputs. SNPs inside a gene and its +/-5kb adjacent regions were first mapped to the gene, and then DESE ran iteratively to produce a list of driver tissues and the corresponding p-values of the associations. We used a Bonferroni corrected significance threshold of 0.05/54 = 9.3e-4.*

3. For the bench-mark setting and the first locus-specific method, falling within 500kb of a credible gene may be too broad.

*Response: We thank the reviewer for this comment. We chose to use a 500kb window to be consistent with our prior publication [4] for defining loci, because it is a typical window used[5], and to be inclusive so as to not miss genes due to long-range LD.*

4. In addition, given the genetic difference between Mendelian and complex diseases, the authors should justify why it is acceptable to use the Mendelian gene for the validation of different methods.

*Response: We have previously observed that Mendelian lipid genes such as PCSK9[6], LPL[7], APOB[8], CETP[9], LDLR[10], etc. have allelic series with rare variants with large effect to common variants [11] with a small effect on lipid levels. While this is an imperfect choice, we believe it is a reasonable choice. Furthermore, we have removed the validation of the methods from the main results of the manuscript.*

5. What do you mean "protective protein-altering lipid alleles", Pls explain the term briefly.

*Response: We have modified the section title to clarify this statement. The section title is now: “Effects of protein-altering lipid alleles with protective effects on CAD, T2D, and NAFLD”.*

6. The phenome-wide association scan looks strange and remains association. The MR methods have been wildly used for causal inference among the traits. Why did not the author use MR for the causation analysis?

*Response: We are unclear on why the reviewer suggests that our PheWAS “looks strange and remains association”. Re: MR, a comprehensive MR PheWAS analysis was not within the scope of analyses we planned to present in this manuscript. Given the imperfections of MR and multitude of sensitivity analyses that would have to be included (e.g. multivariate MR), we believe that this would warrant a separate manuscript to fully address MR for lipid alleles across a wide-range of traits. Furthermore, our top PheWAS findings are already corroborated by previously published MR analysis, including the causal effect of blood lipids on coronary artery disease and other cardiovascular phenotypes [12], on dementia [13], on Alzheimer’s disease [14], on hemoglobin [15], and on cholelithiasis [16]. We now highlight all this evidence of causality in our results and discussion.*

*Text added to the Results:*

*A recent wide-ranging Mendelian Randomization analysis confirmed the causal effect of circulating lipids, not only for coronary artery disease, but other cardiovascular outcomes [42].*

*Recent Mendelian Randomization studies also provide evidence for the causal effect of lipids on risk for dementia [44] and Alzheimer’s disease [45].*

*Text added to the Discussion:*

*Our results are corroborated by a recent Mendelian Randomization meta-analysis study in the FinGen and UK Biobank cohorts, where lower total cholesterol and LDL-C levels were causally associated with reduced risk of cholelithiasis [69].*

*While most of our significant PheWAS findings could be confirmed via Mendelian Randomization studies, we cannot exclude the possibility of spurious associations due to pleiotropy.*

7. Given there a lot of results this study has produced, it may necessary to make a web paper for readers to quickly search or browse the prioritized genes and corresponding evidences.

*Response: We thank the reviewer for this suggestion. We have worked with the Accelerating Medicines’ Human Genetics Knowledge Portal to create a browser for our primary results that will be made publicly available upon acceptance of the manuscript. The reviewer can preview these pages by login to URL below using the provided username and password. https://hugeamp.org:8000/research.html?pageid=GLGC_149*

*User: GLGC*

*PW: GLGC_review*

*The following text has been added to the Data Availability section:*

*A web browser displaying the gene prioritization and PheWAS results is available at https://hugeamp.org:8000/research.html?pageid=GLGC_149.*

8. It is not clear what are the new major findings of this study compared with the previous studies particularly the [ref. 40].

*Response: The new major findings of this study not included in the Graham et al publication are: (1) The prioritization of genes for lipid GWAS variants; (2) The inclusion of results for the X chromosome; (3) the identification of sex-specific lipid alleles; and, finally, (4) our focus on lipid biology by prioritizing implicated genes, identifying novel phenotypes and diseases associated with lipid levels, and predicting candidate drug target genes.*

*We have clarified the text in the first paragraph of the Discussion section to highlight these new major findings.*

*Text edited in the Discussion: Here, we add X-chromosome and sex-specific discovery results. We also focus on lipid biology by prioritizing implicated genes, identifying novel phenotypes and diseases associated with genetically-predicted lipid levels, and predicting candidate drug target genes.*

9. A minor point is that Figure 2 has too much empty space.

*Response: We agree and the new Figure 2 has less empty space.*

Reviewer #2:

Kanoni et al. present a comprehensive genetic analysis of blood lipids in a multi-ancestry meta-analytic setting. They address three critical issues in lipids genetics: 1. Putative causal gene implication; 2. Utility of genetically-predicted lipid levels; and 3. sex-specific lipids genetic associations. Overall, I find the paper is most clearly written and addresses important issues in lipids genetics and genomics. However, I have a few concerns about some technical aspects of the presented analyses. I hope that the authors can address these issues in the revision.

*Response: We thank the reviewer for the positive feedback as well as the below suggestions to improve our manuscript.*

1. On putative causal gene implication. This is the main emphasis of the paper, and the authors present the results from the six available methods. My concern is that some of the claims in the article diverge to evaluating the various gene implication methods, and the data used in this paper is not designed for this purpose. Firstly, the authors construct a gold and silver set of genes and use them to benchmark the performance of various gene implication methods. I am concerned about how "precision" and "recall" are computed in this process. I don't think the terminologies are appropriate here. Particularly, the "recalls" (they are generally low across methods) are only valid if none of the genes outside the gold/silver set are causal genes, which is too strong an assumption. Secondly, even if we ignore the caveats of defining "precision" and "recall," the cutoffs used by different methods for prioritizing causal genes are seemingly very different and difficult to compare. E.g., the TWAS method controls FDR, the colocalization method controls local FDR, and other methods do not offer type I error control, and the threshold seems arbitrary. On this issue, instead of presenting Figure 1B, the complete precision-recall curve for each method seems more informative and appropriate. Thirdly, can the authors provide some reasoning for the superior performance of PoPS? Specifically, do the training weights (on genomic features) offer any biological insights? If only human_multiple from GTEx data is used, how is its performance compared to the TWAS and the colocalization analysis? How relevant are the mouse gene feature datasets concerning the gold and silver sets, respectively? In summary, I think the current presentation of this analysis opens the door for a series of technical questions like the above. Alternatively, I suggest the authors focus on the common set of causal genes identified by multiple methods and establish their biological relevance (instead of comparing gene implication methods).

*Response: We have substantially revised the gene prioritization section of the manuscript to address the reviewer’s constructive criticisms. First, we have moved the text on evaluating the various gene implication appropriates to the Methods as our approach for selecting lipid genes to follow-up on with text mining and Therapeutic Targets results. We include revised text in the Methods section to justify why we were taking a subset of the genes forward for further investigation. Figure 1B has been removed from the main text. Second, we no longer use the terms “precision” and “recall”. Instead we have reframed as “Proportion of prioritized genes in the gold standard set” vs “Proportion of correctly identified gold standard genes among all prioritized genes”. Third, we used the thresholds for each method consistent with suggestions on how to use these methods in prior publications. Based on changing the DEPICT threshold, there are minor differences in the resulting statistics.*

*Finally, we revised the Discussion to remove the notion that we are comparing methods. Our focus was on lipid loci, and simulations are necessary to compare the various prioritization approaches. The Discussion now states:*

*We first sought to identify methods for prioritization of functional genes at GWAS loci by performing six gene prioritization methods. Lipids are an excellent exemplar phenotype for gene prioritization algorithms because of a wealth of GWAS loci (~1000), Mendelian dyslipidemia genes (21), and mouse dyslipidemia phenotypes observed in gene knock-outs (740).*

2. On lipid PRS associations. The analysis presented in this section shares many common properties with the TWAS analysis. As pointed out by Barfield 2018 Genetic Epi, Mancuso 2019 Nature Genetics, and Hukku 2022 AJHG, some of the signals are simply due to some GWAS hits for lipids and other traits in modest LD. Do the authors also observe such phenomena. If so, do they impact the interpretations?

*Response: Most of our significant PheWAS findings are corroborated by previously published MR analyses, including a causal effect of blood lipids on coronary artery disease and other cardiovascular phenotypes [12], on dementia [13], on Alzheimer’s disease [14], on hemoglobin [15], and on cholelithiasis [16]. We are highlighting all this evidence of causality in our Results and Discussion. However, we cannot exclude the possibility of spurious pleiotropy within our PheWAS results and acknowledge this as a limitation in the Discussion. Please see response to Review #1, comment #6 for changes made to the text.*

3. On sex-specific lipids associations. The reasoning of p-values for the sex-stratified analysis is questionable and not convincing. The caveats of using p-values to classify the presence and absence of true associations have been well-known in applications like defining replication successes and identifying tissue-specific eQTLs. Presenting the estimated effects from the stratified analysis and computing some heterogeneity measures would be better evidence for this specific analysis.

*Response: We thank the Reviewer for a very valid comment about basing nonsignificance or the difference of association on the P-values. To address this, we have added the sex-heterogeneity P-value to Tables S11 & S12 and edited the manuscript to include this heterogeneity test information for all loci showing significant association in sex-stratified metaanalysis only.*

*Text edited in the Results: We identified twelve loci in females and four in males that reached genome-wide significance in the sex-stratified analysis (p < 5x10^-8^ ; Tables S10, S11 and S12 ) but not in the sex-combined meta-analysis. As variants may show association to a single sex for reasons unrelated to biological sex differences, including differences in sample sizes between groups, we additionally tested for heterogeneity by sex for these variants in GLGC participating cohorts with close to equal number of males and females. Of the sixteen loci, eight showed significant sexheterogeneity (p < 0.0031, Bonferroni for sixteen tests). For example, the non-synonymous variant rs34372369 (EPHA1, p.Pro582Leu) is associated with nonHDL only in females (male pvalues > 0.05) and shows significant sex-heterogeneity (p-value 0.0012). This variant has been previously found to be linked with expression levels of the sex hormone binding globulin gene (SHBG) more strongly in males than females [49], suggesting a possible reason for the difference in observed associations. We additionally sought to replicate the sex-heterogeneity results of these variants in 8 independent multi-ancestry cohorts (N=311,639, 77% nonEuropean ancestry, Table S13). However, we did not detect significant differences in effect sizes between sexes for these variants after accounting for the number of tests (p-value > 0.0031, Table S14), potentially due to the limited sample size or difference in ancestry makeup.*

4. Some minor details are also needed for the Method section. E.g., the coding variants method uses a 99% credible set. Are the credible sets constructed by single-SNP or fine-mapping analysis? In coloc analysis, are the fine-mapping results of GTEx eQTL used or just the simple single-SNP association results? These details will help the readers to interpret the results presented in the paper.

*Response: We added the following text to the Methods section to clarify the construction of the credible sets:*

*We defined regions for construction of the credible sets as ±500kb around each index variant. We used Bayes factors (BFs) for each variant from the MR-MEGA output and generated the credible sets within each region by ranking all variants by BF and calculating the number of variants required to reach a cumulative probability of at least 99%.*

*For the coloc analysis, we defined a window around each of the GWAS signals and used all independent genetic signals with the window. We made the following clarification to the Methods: We ran an eQTL colocalization using GTEx v8 eQTL summary statistics within each of our defined windows for all lipid traits.*

*REFERENCES 1. Yang J, Lee SH, Goddard ME, Visscher PM: GCTA: a tool for genome-wide complex trait analysis. Am J Hum Genet 2011, 88:76-82.*

*2. Liu DJ, Peloso GM, Zhan X, Holmen OL, Zawistowski M, Feng S, Nikpay M, Auer PL, Goel A, Zhang H, et al: Meta-analysis of gene-level tests for rare variant association. Nat Genet 2014, 46:200-204.*

*3. Ramdas S, Judd J, Graham SE, Kanoni S, Wang Y, Surakka I, Wenz B, Clarke SL, Chesi A, Wells A, et al: A multi-layer functional genomic analysis to understand noncoding genetic variation in lipids. Am J Hum Genet 2022, 109:1366-1387.*

*4. Graham SE, Clarke SL, Wu KH, Kanoni S, Zajac GJM, Ramdas S, Surakka I, Ntalla I, Vedantam S, Winkler TW, et al: The power of genetic diversity in genome-wide association studies of lipids. Nature 2021, 600:675-679.*

*5. Fauman EB, Hyde C: An optimal variant to gene distance window derived from an empirical definition of cis and trans protein QTLs. BMC Bioinformatics 2022, 23:169.*

*6. Cohen JC, Boerwinkle E, Mosley TH, Jr., Hobbs HH: Sequence variations in PCSK9, low LDL, and protection against coronary heart disease. N Engl J Med 2006, 354:1264-1272.*

*7. Khera AV, Won HH, Peloso GM, O'Dushlaine C, Liu D, Stitziel NO, Natarajan P, Nomura A, Emdin CA, Gupta N, et al: Association of Rare and Common Variation in the Lipoprotein Lipase Gene With Coronary Artery Disease. JAMA 2017, 317:937-946.*

*8. Peloso GM, Nomura A, Khera AV, Chaffin M, Won HH, Ardissino D, Danesh J, Schunkert H, Wilson JG, Samani N, et al: Rare Protein-Truncating Variants in APOB, Lower Low-Density Lipoprotein Cholesterol, and Protection Against Coronary Heart Disease. Circ Genom Precis Med 2019, 12:e002376.*

*9. Nomura A, Won HH, Khera AV, Takeuchi F, Ito K, McCarthy S, Emdin CA, Klarin D, Natarajan P, Zekavat SM, et al: Protein-Truncating Variants at the Cholesteryl Ester Transfer Protein Gene and Risk for Coronary Heart Disease. Circ Res 2017, 121:81- 88.*

*10. Natarajan P, Peloso GM, Zekavat SM, Montasser M, Ganna A, Chaffin M, Khera AV, Zhou W, Bloom JM, Engreitz JM, et al: Deep-coverage whole genome sequences and blood lipids among 16,324 individuals. Nat Commun 2018, 9:3391.*

*11. Global Lipids Genetics Consortium, Willer CJ, Schmidt EM, Sengupta S, Peloso GM, Gustafsson S, Kanoni S, Ganna A, Chen J, Buchkovich ML, et al: Discovery and refinement of loci associated with lipid levels. Nat Genet 2013, 45:1274-1283.*

*12. Allara E, Morani G, Carter P, Gkatzionis A, Zuber V, Foley CN, Rees JMB, Mason AM, Bell S, Gill D, et al: Genetic Determinants of Lipids and Cardiovascular Disease Outcomes: A Wide-Angled Mendelian Randomization Investigation. Circ Genom Precis Med 2019, 12:e002711. 13. Zhang X, Tian Q, Liu D, Geng T, Xu X, Ge S, Zheng D, Wu L, Song M, Hou H, et al: Causal association of circulating cholesterol levels with dementia: a mendelian randomization meta-analysis. Transl Psychiatry 2020, 10:145.*

*14. Tan JS, Hu MJ, Yang YM, Yang YJ: Genetic Predisposition to Low-Density Lipoprotein Cholesterol May Increase Risks of Both Individual and Familial Alzheimer's Disease. Front Med (Lausanne) 2021, 8:798334.*

*15. Leong A, Chen J, Wheeler E, Hivert MF, Liu CT, Merino J, Dupuis J, Tai ES, Rotter JI, Florez JC, et al: Mendelian Randomization Analysis of Hemoglobin A1c as a Risk Factor for Coronary Artery Disease. Diabetes Care 2019, 42:1202-1208.*

*16. Chen L, Yang H, Li H, He C, Yang L, Lv G: Insights into modifiable risk factors of cholelithiasis: A Mendelian randomization study. Hepatology 2022, 75:785-796.*

**Second round of review**

**Reviewer 1**

I appreciates the efforts of the authors to address my previous comments. I have no additional comments on the manuscript.

**Reviewer 2**

I appreciate the authors' efforts in carefully addressing the comments and revising the manuscript. I believe that the revised manuscript is now suitable for publication.
